# What are the effects of different elements of media on radicalization outcomes? A systematic review

**DOI:** 10.1002/cl2.1244

**Published:** 2022-06-08

**Authors:** Michael Wolfowicz, Badi Hasisi, David Weisburd

**Affiliations:** ^1^ Institute of Criminology, Faculty of Law Hebrew University of Jerusalem Mount Scopus Jerusalem 91905 Israel; ^2^ Department of Criminology, Law and Society George Mason University Fairfax VA USA

## Abstract

**Background:**

Most national counter‐radicalization strategies identify the media, and particularly the Internet as key sources of risk for radicalization. However, the magnitude of the relationships between different types of media usage and radicalization remains unknown. Additionally, whether Internet‐related risk factors do indeed have greater impacts than other forms of media remain another unknown. Overall, despite extensive research of media effects in criminology, the relationship between media and radicalization has not been systematically investigated.

**Objectives:**

This systematic review and meta‐analysis sought to (1) identify and synthesize the effects of different media‐related risk factors at the individual level, (2) identify the relative magnitudes of the effect sizes for the different risk factors, and (3) compare the effects between outcomes of cognitive and behavioral radicalization. The review also sought to examine sources of heterogeneity between different radicalizing ideologies.

**Search Methods:**

Electronic searches were carried out in several relevant databases and inclusion decisions were guided by a published review protocol. In addition to these searches, leading researchers were contacted to try and identify unpublished or unidentified research. Hand searches of previously published reviews and research were also used to supplement the database searches. Searches were carried out until August 2020.

**Selection Criteria:**

The review included quantitative studies that examined at least one media‐related risk factor (such as exposure to, or usage of a particular medium or mediated content) and its relationship to either cognitive or behavioral radicalization at the individual level.

**Data Collection and Analysis:**

Random‐effects meta‐analysis was used for each risk factor individually and risk factors were arranged in rank‐order. Heterogeneity was explored using a combination of moderator analysis, meta‐regression, and sub‐group analysis.

**Results:**

The review included 4 experimental and 49 observational studies. Most of the studies were judged to be of low quality and suffer from multiple, potential sources of bias. From the included studies, effect sizes pertaining to 23 media‐related risk factors were identified and analyzed for the outcome of cognitive radicalization, and two risk factors for the outcome of behavioral radicalization. Experimental evidence demonstrated that mere exposure to media theorized to increase cognitive radicalization was associated with a small increase in risk (*g* = 0.08, 95% confidence interval [CI] [−0.03, 19]). A slightly larger estimate was observed for those high in trait aggression (*g* = 0.13, 95% CI [0.01, 0.25]). Evidence from observational studies shows that for cognitive radicalization, risk factors such as television usage have no effect (*r* = 0.01, 95% CI [−0.06, 0.09]). However, passive (*r* = 0.24, 95% CI [0.18, 0.31]) and active (*r* = 0.22, 95% CI [0.15, 0.29]) forms of exposure to radical content online demonstrate small but potentially meaningful relationships. Similar sized estimates for passive (*r* = 0.23, 95% CI [0.12, 0.33]) and active (*r* = 0.28, 95% CI [0.21, 0.36]) forms of exposure to radical content online were found for the outcome of behavioral radicalization.

**Authors' Conclusions:**

Relative to other known risk factors for cognitive radicalization, even the most salient of the media‐related risk factors have comparatively small estimates. However, compared to other known risk factors for behavioral radicalization, passive and active forms of exposure to radical content online have relatively large and robust estimates. Overall, exposure to radical content online appears to have a larger relationship with radicalization than other media‐related risk factors, and the impact of this relationship is most pronounced for behavioral outcomes of radicalization. While these results may support policy‐makers' focus on the Internet in the context of combatting radicalization, the quality of the evidence is low and more robust study designs are needed to enable the drawing of firmer conclusions.

## 1 PLAIN LANGUAGE SUMMARY

### 1.1 Experimental evidence concerning media effects on radicalization is limited, inconclusive, and of low quality

Exposure to radical content over the Internet has a salient relationship with both cognitive and behavioral outcomes of radicalization, compared to other media‐related factors. However, the quality of this evidence is low and results should be interpreted in light of this.

### 1.2 What is this review about?

Exposure to and consumption of media has long been pointed to as a possible risk factor for radicalization. In recent years, the Internet has come under increasing scrutiny, with a sub‐field of research examining cyber‐radicalization. Yet little is known about the magnitude of individual‐level media‐effects as risk factors for radicalization. In addition, it is not known if, and to what degree the Internet has greater effects than other types of media, or whether and to what degree the nature of the content being consumed matters. There are therefore wide gaps in the body of knowledge concerning media effects and radicalization.

Media‐related factors pertain to human‐media interactions and relationships and therefore included a number of domains, including (1) the medium itself (e.g., TV, radio, Internet), (2) platforms (e.g., Western vs. non‐Western TV, Facebook, Twitter, etc.), (3) content (e.g., violent, pro‐social), (4) activities and behaviors (e.g., time spent engaged), and (5) attitudes (e.g., attachment). For the purposes of this review, all such media‐related factors were considered for inclusion.

This review looked at individual‐level media effects on two outcomes of radicalization, cognitive and behavioral, with cognitive radicalization being limited to support, justification of, or a willingness/intention toward the use of radical violence in the name of a cause or ideology, and behavioral radicalization pertaining to the actual involvement in such violence.

The aim of this review was to identify if, and to what degree, media‐effects can be identified as risk factors for radicalization.

This Campbell systematic review examines the relationships between media‐related factors and radicalization. The review summarizes evidence from 4 experimental and 49 observational studies and synthesizes the effects of 23 media‐related risk factors across the two outcomes of cognitive and behavioral radicalization.

### 1.3 What studies were included?

This review includes 53 studies spanning the period 2002–2020, with most published between 2016 and 2020. The studies mostly used samples of respondents from Europe and North America, and several from Middle Eastern and Asian countries. Of the included studies, 4 were experimental (12 samples) and 49 were observational (64 samples).

### 1.4 What are the main findings of this review?

Results from experimental studies indicate that simple, one‐time exposure to mediated content is associated with a small increase in the risk of radicalization. For individuals high in trait aggression, there is an equally small increase in risk.

The results from observational studies show that the magnitude of the relationship between simple usage of different media and radicalization is essentially inconsequential, with estimates close to zero. However, when usage of media was measured with reference to specific types of content or activities, such as the consumption of or posting of political content, small but potentially meaningful relationships are found.

For some attitudinal risk factors, such as perceptions of media bias, estimates indicate only small relationships with radicalization, whereas the estimates for others, such as attachment to online networks, point to more salient relationships. The largest estimates pertain to both passive and active forms of Internet‐based exposure to content defined as specifically radical.

The reliability of these results is tempered by multiple sources of bias inherent in the cross‐sectional studies, as well as the experimental studies that give rise to these results, including the inability to establish temporal ordering. Additionally, alternative theoretical explanations suggest that the results may suffer from confounding with factors such as age, gender, and self‐control.

### 1.5 What do the findings of this review mean?

Simple media consumption is unlikely to be associated with any significant risk of radicalization, including one‐time exposures to radical content. Despite alternative hypotheses that increases in risk are a function of certain psychological traits, such as aggression, differences in the magnitude of estimates are negligible.

On the other hand, Internet‐mediated exposure to radical content, whether passive or active, is associated with a significantly stronger relationship with radicalization than other types of media‐related risk factors. When compared to other known risk factors for cognitive radicalization (non‐media), the relative magnitudes of the estimates are moderate. However, when compared to other known risk factors for behavioral radicalization, the relative sizes of the estimates are considerable.

The ability to draw conclusions from the results is limited by the fact that studies suffer from multiple sources of bias.

### 1.6 How up‐to‐date is this review?

The review authors searched for studies up to mid‐2020.

## 2 BACKGROUND

### 2.1 The issue

#### 2.1.1 The media‐radicalization relationship

Since the early days of terrorism research, scholars have consistently emphasized the integral role of the media as a key factor in promoting terrorism. The media's reporting on and portrayal of terrorist attacks and groups provides them with “free advertising.” This exposure provides terrorists, groups, and their proponents with a vehicle for highlighting their grievances and spreading their message. In doing so, terrorists aim to attract new supporters and recruits, and strengthen their image among their current supporters (Weimann, 2005). The media also reports on and portrays a range of social issues that may highlight forms of discrimination or violence against different groups—both locally and abroad—which can increase feelings of anger, deprivation, and identification with the oppressed group. All these factors can increase the likelihood that an individual will support what they perceive to be the use of defensive violence, or even engage in it themselves (Wolfowicz et al., 2020).

These types of effects have received extensive attention in the ever‐growing literature on individual radicalization, which focuses on the factors that increase (or decrease) the likelihood of the development of attitudes that justify terrorism, and which could therefore underpin a turn to radical behaviors such as terrorism. In this literature, which has primarily developed only in the last 15 years, research on the role of traditional media has shifted to focusing on the role of new media, namely the Internet, and social media in particular. These medias have been successfully leveraged by radical groups and their supporters to spread their message, greatly increase their support base, and from this base generate new recruits (Scrivens & Conway, 2019).

There is little doubt that media exposure in general can and does impact a range of cognitive outcomes, from emotions to attitudes and perceptions, as well as behavioral outcomes, whether on account of cognitive changes, imitation, or other mechanisms. An extensive literature exists that demonstrates these effects with respect to a range of deviant, criminal, and violent cognitive and behavioral outcomes. However, despite what seems to be a significant amount of attention given to the topic of media effects on radicalization, which encompasses a form of violent cognition (a positive evaluation of violence) and behavioral outcomes (Wolfowicz et al., 2020), there is a worrying lack of quantitative research (Gill et al., 2017; LaFree, 2017; Whittaker, 2018).

### 2.2 Media‐related risk factors

Like the broader literature of media effects on violent cognitions and behaviors, there is a debate about the magnitude, and even the direction of the effects of media on radical cognitions and behaviors (Ferguson, 2016). Previous research has identified significant overlaps between radical cognitions and behaviors with violent cognitions and behaviors, as well as the risk and protective factors for these outcomes (Wolfowicz et al., 2020). Given these overlaps, it would be reasonable to expect that the media effects for general criminal and criminal‐analogous outcomes should be similar for radical outcomes. In this respect, it can be expected that there are differential effects for different mediums and content (Awan, 2007a, 2007b; Awan et al., 2011).

Some of the earliest literature on modern terrorism emphasized the potential role of television news‐broadcast media in fostering the phenomenon. As Social cognitive theorist Albert Bandura (1990) wrote, “They [*terrorists]* use television as the main instrument for gaining sympathy and supportive action” (p. 8). As a multidimensional media, providing news, entertainment, and broadcast, television can potentially affect sympathy and support (cognitive radicalization), or supportive action (behavioral radicalization) in multiple ways (Matusitz, 2013). First, as a source of exposure to violent imagery, either real or fictitious, television can activate aggression and increase desensitization. These images, as well as general news and political news about social issues and conflicts, can also serve as source of imitation, and grievance. Television can also serve as a medium for the broadcasting of both explicit and more subtly ideological messaging, from politicians and commentators to religious sermons and other programming (Matusitz, 2013).

Some studies have found that when terror attacks receive more television coverage, interest in associated groups on the Internet increases (Jetter, 2019). The interplay between news and broadcasting can also potentially contribute to radicalization. For example, certain populations may be exposed to elements of a materialistic western culture through television. These depictions may subsequently be referred to in a disparaging way by preachers (Mousseau, 2011). Television, news media, and selected propaganda items may expose a receiver to images, scenes, and messages that highlight attacks on a group with whom they identify. For example, items that highlight the acts of extremists representing an opposing group (e.g., right‐wing vs. left wing), or military or state attacks on in‐group members. Interviews with former terrorists and non‐violent radicals reveal that these forms of exposure can play a central role at different stages of the radicalization process (Baugut & Neumann, 2020a). Additionally, mainstream media reports of attacks against certain groups, both locally and abroad, may also be subjectively selected by propagandists and then re‐distributed through other mediums. This form of "hybrid propaganda" has been found to have played a key role in the radicalization of radical offenders in Europe (Baugut & Neumann, 2019a).

Another key factor is the potential for news‐media bias to increase feelings of hostility. In a laboratory experiment Neumann et al. (2018) exposed Muslim participants, who differed in their religious‐fundamentalist beliefs, to either positive or negative news stories about Muslims. The study found that negative presentations were associated with perceived media bias toward Muslims and increased participants' feelings of general anger. The effect was greater for participants who had higher scores on the religious‐fundamentalist beliefs scale. These results are in line with evidence concerning the “hostile media effect.” A meta‐analysis found that the relationship between increased media consumption and perceptions of a hostile media were quite modest, with an average effect of *r* = 0.296. The study found that the medium (e.g., newspapers, television etc.) did not have a statistically significant impact on this result. However, greater involvement with the issue depicted in the media had a statistically significant, positive impact (Hansen & Kim, 2011).

A recent study based on interviews with dozens of incarcerated terrorism offenders found that they believed that exposure to media did not have a significant impact on their beliefs or attitudes. However, they also believed that the media did have a significant impact on others and played a role in formulating negative attitudes toward their in‐group within the general population (Baugut & Neumann, 2020b, 2021). This type of media perception is known as the “third person effect,” which posits that individuals perceive the media to have greater sway and influence over others than over themselves (Paul et al., 2000). That the terrorists from the above noted study all displayed evidence of the third‐person effect provides an early indication that a potentially wide variety of media effects may be applicable to radicalization.

In recent years there has been a growing trend in which radical groups have used the medium of videogames to indoctrinate and to recruit. Different groups, such as ISIS, have created high‐quality games in which players operate as terrorists, killing opponents who represent the groups' real‐life adversaries. Beyond these tailor‐made games, the interactive features of mainstream games are rife with hate speech, racism, and threats of violence. It could be theorized that even these “ordinary” video games can increase the risk of radicalization, as they do for generalized violent cognitions and behaviors. However, as with respect to the research on video game effects more generally, there have been suggestions that video games could provide prosocial effects and thereby reduce the risk of radicalization (Menendez‐Ferreira et al., 2020).^1^

Just as in the broader field of media‐effects research, while television and the Internet have commanded the bulk of researchers' attention, the literature has also discussed the role of other forms of traditional media in radicalization. For example, the role of radio has been discussed in the context of right‐wing radicalization (Hackett & Soares, 2015; Hamm & Spaaij, 2017) and radicalization in certain parts of Africa (Yanagizawa‐Drott, 2014). Similarly, the role of music has figured prominently in research on right‐wing radicalization (Gaudette et al., 2020; Pieslak, 2015); and more recently regarding Islamist radicalization (Pieslak, 2015). Research has also focused on multimodal mediums, such as music videos, videogames, and short‐clip productions, with such content believed to have stronger effects on emotional and cognitive outcomes, in line with the objectives of radical groups (Winkler & Pieslak, 2019).

Despite the move to digital sources, print‐media remains an important source for news and for conveying different ideological positions in many places around the world. In Pakistan, the ideological slant local newspapers have given to the portrayal of drone strikes on terrorist targets has been referred to as a “major source” of radicalization (Raza & Awan, 2013). Additionally, across the Middle East, and even in places such as downtown London, books such as Adolf Hitler's Mein Kampf and the fictional Protocols of the Elders of Zion are sold alongside more recent radical publications (Patterson, 2016). These books and others, such as *The Turner Diaries* (1978) have been implicated in the radicalization of several terrorists involved in attacks, including the Oklahoma City Bombings (Berger, 2016; Mills et al., 2019).

Despite the importance of these “old media,” it cannot be denied that most of the attention in recent years has shifted to examining and understanding the role of the Internet as a media source for radicalization. However, just as Internet technology has undergone significant change over the last two decades, so too has the focus of research. Extremist groups were early adopters of the world wide web, with web pages created as early as 1996 which were used to promote their causes, provide access to content, and enable discussion between supporters (Erez et al., 2011). The move to discussion forums was a key innovation for radical groups as it not only provided a place for like‐minded individuals to communicate but for the curious to hear from the more well versed and experienced. Already in 1995, members of the neo‐Nazi movement had created *StormFront*, a platform which still operates today with more than 150,0000 active members (Bowman‐Grieve, 2009). Discussion forums continue to be viewed as a media‐related risk factor for radicalization (Moskalenko et al., 2022). They are even central to some ideologies and movements, such as *INCELS* (involuntary celibates), which are guided by a deeply misogynistic ideology. While INCEL discussions groups have many thousands of members, high‐profile attackers have often been found to have been highly active in their postings (Moskalenko et al., 2022).

Today, social media platforms, such as Facebook, Twitter, and YouTube, have garnered much of the attention in discussions about the potential for media to serve as a risk factor for radicalization. Together with discussion forums and chat rooms, social media provides opportunities for likeminded individuals to connect, to seek reinforcement for previously held beliefs, and share content. In this regard, these platforms provide for the sharing of multimodal content, such as digital images and videos. Multimodal content is likely to have stronger psychological effects than other unimodal or bimodal content (Conway & MacDonald, 2020). As with the study of media‐effects more generally, different platforms offer opportunities for both passive and active forms of consumptions. Traditional media platforms, such as print media and television, provide opportunities for passive exposure, in which a consumer is not required to participate or engage in any specific way. Internet‐based platforms however, whether discussion forums, social media, or mobile communication applications, in addition to opportunities for passive exposure, also provide opportunities for active exposure, in which the consumer can actively interact with the media, or with others who are also interacting with or through the media (Coyne et al., 2018). Radical groups and ideologies of all stripes have some sort of presence on these platforms (Holbrook, 2021; Weimann, 2016). These are also the type of platforms where users are most likely to seek out radical content produced by groups such as Al‐Qaeda and ISIS (Frissen, 2021).

### 2.3 The nature of the relationship between media‐related factors and radicalization

The potentially negative cognitive and behavioral effects of media have been analyzed for close to a century, with the first studies examining the effects of movies. With the proliferation of home TV sets in the 1950s, and several sensationalized cases of violence described in the media as having been the result of imitation of TV violence, this field of research expanded greatly (Sparks et al., 2009). In seeking to explain and test the ways in which media may potentially increase the risk of deviant or criminal cognitions and behaviors, researchers have drawn extensively on social learning and social cognitive perspectives. Social learning has also provided the basis for several other prominent, media‐specific theories (Allen et al., 2018; Phillips, 2017).

According to criminology's social learning theory, deviant attitudes and behaviors are learned through the same mechanisms as normative attitudes and behaviors, namely through *differential associations*, the *definition* that they provide, *imitation*, and *differential reinforcement*. Differential associations can be peers, family, role models or other individuals. Their opinions and positions on a given issue or behavior, as well as the behaviors they themselves engage in, provide an individual with a balance of definitions in favor or against the given behavior. The experiencing and observing of the behaviors of differential associations also provides a source of imitation of what those behaviors should look like. Additionally, through experiences and observations, an individual will gauge what sort of reinforcement—positive or negative—engagement in the behavior tends to garner. When an individual has a greater proportion of differential associations holding a certain attitude, or engaging in a behavior, when they are exposure to it more often, and when they see that it generates positive reinforcement, they are more likely to adopt the attitude or behavior. In this regard, learning can also be mediated, and pieces of media‐content, from books to television programs can also serve as differential associations. Even more so on the Internet, individuals can establish or maintain associations with, or follow the behaviors of actual individuals (Akers, 1998).

Differential associations are conditioned by frequency of interaction, duration of association, intensity of association, and the subjective priority (importance) ascribed to them. Sometimes the intensity of mediated differential associations can be even stronger than for non‐mediated ones. Additionally, mediated learning may provide for a greater source of imitation due to its visual nature—as in the case of television, movies, videogames and the Internet—and the frequency and repetitiveness of exposures to content and the definitions, or messages that they provide with respect to a given attitude or behavior (Akers, 1998).

Another variant of SLT that was put forth by Bandura (1978) provided the basis for his development of a more cognitively oriented theory, Social Cognitive Theory (SCT). While the theory operates according to the same mechanisms of observation, experience and learning, the way in which it can lead to deviant outcomes is through desensitization and moral disengagement. For example, repeated exposure to messages that demonize or dehumanize a particular individual, type of individual, group or thing, can lead to a moral disengagement in which violence becomes legitimate (Bandura, 1977, 1990, 2001, 2009).

Other variants of both SLT and SCT have been developed to focus on media‐effects specifically. For example, the General Aggression Model (GAM) holds that exposure to media depicting violence, or which includes pro‐violent messaging, primes aggressive thoughts and emotions, arousing and activating aggressive cognitions at the neural level. While arousal and activations may initially be measured in attitudinal expressions, such as in the case of learning theory, it is these cognitive effects operating at the level of neural pathways that may ultimately manifest in outward behaviors (Allen et al., 2018). Both social learning and cognitive perspectives also give weight to the role of reciprocal determinism. That is, just as “birds of a feather flock together,” and individuals tend to gravitate toward those with beliefs similar to their own, they also have a tendency to select bias‐congruent media. As such, pre‐existing biases shape media consumption, and consumption reinforces and confirms prior beliefs (Huesmann & Taylor, 2006). This process has been described as a reinforcing spiral (Slater, 2007), and can lead to the formation of ideologically homogenous “echo chambers,” insular networks dominated by one‐sided presentations of ideas, opinions, and attitudes (Stevens & Neumann, 2009; Sunstein, 2009).

The meta‐analytic contributions to the field of media‐effects provide strong evidence for the social learning and cognitive perspectives. On the one hand, it has been found that pro‐social media exposure improves pro‐social cognitions and behaviors, whilst anti‐social or deviant media exposure promoted deviant cognitions and behaviors. Similarly, pro‐social media has been found to reduce the likelihood of aggression, whereas anti‐social, deviant, and violent media has been found to increase it. Moreover, great consistency has been found between these positive and negative effects, which have been found to have nearly identical magnitudes (Coyne et al., 2018; Greitemeyer & Mügge, 2014; Mares & Woodard, 2005).

In opposition to the media socialization paradigm, in which media usage is seen as a predictor of the outcome, there is the media selectivity paradigm, in which media usage is an outcome of the theorized predictors (Slater, 2008). Due in large part to the designs of studies in the field of media‐effects, it is often difficult to disentangle whether certain types of media usage increase outcomes such as aggression, or whether those high in aggression are simply more prone to engage with problematic media. Similarly, failure to control for potential confounder, especially those known to be associated with the outcome, such as gender, mean that effects may simply represent the relationship between the third variable and the outcome. Some studies have found that when controlling for such factors, effect size estimates are as much as half of bivariate correlations. These findings provide support for the media‐selectivity perspective as they demonstrate that a large proportion of the variance is explained by individual characteristics (Anderson et al., 2010).

A related issue is likely to exist with respect to radicalization as well. One of the most important risk factors for radicalization is low self‐control, as well as related risk factors such as thrill/sensation seeking (Wolfowicz, Litmanovitz, et al., 2021). Some research indicates that individuals with low self‐control may be more likely to engage with problematic media in several ways. First, they may be more prone to spending more time engaging with media in general, as well as developing Internet addiction (Li et al., 2021). By spending more time using media, they may therefore be at a heightened risk of encountering content that could increase risk for radicalization. Indeed, there is already some evidence that low self‐control is a predictor of exposure to radical content (Pauwels et al., 2020; Hawdon et al., 2019). Additionally, an individual's self‐control mediates the influence that exposure to media has on them. Based on these issues, it has been suggested that self‐control perspectives inherently suggest that the relationship between media and deviance is a spurious one (Hermann, 2011).

#### 2.3.1 Meta‐analytic contributions to the study of media effects

While there are some exceptions (e.g., Ferguson et al., 2020), over the years there have been a slew of meta‐analyses that have produced fairly consistent results showing that exposure to violent television (and movies) has a modest effect on violent cognitions and behaviors (see Comstock et al., 2014 for a review of previous systematic reviews). When it comes to the Internet and social media as mediums, most meta‐analytic research point to small relationships (of about *r* = 0.10) with psychological outcomes—such as self‐esteem and depression (Appel et al., 2020). However, when it comes to delinquent behaviors, average effect sizes of *r* = 0.21 have been found (Vannucci et al., 2020). This effect size is almost identical in magnitude to the effect sizes that have been found for exposure to television violence (Comstock et al., 2014). Other forms of media exposure have also been found to have pooled effect sizes of a similar magnitude on analogous outcomes, such as the effects of music on anti‐social behaviors (Timmerman et al., 2008) and violent video games (e.g., Comstock et al., 2014; Ferguson et al., 2020).

The findings mirror those of other areas of investigation into risk factors for related cognitive and behavioral outcomes, (such as violent cognitions and behaviors, or suicidal thoughts and behaviors), in which effect sizes are almost always larger for the cognitive outcome—which is generally more prevalent. For factors where effect sizes are found to be larger for the behavioral outcomes, it has been suggested that this could be an indication of them serving as mediators of the continuity between the given cognitive and related behavior (Wolfowicz, Weisburd, et al., 2021a). Of all media‐related factors, such a finding has been made only with respect to violent video‐games (e.g., Comstock et al., 2014; Ferguson et al., 2020). This evidence points to a small but potentially important role for at least some mediums to play a role not only in the risk of development of deviant cognitions and behaviors but as a mediator, in which in increases the risk of deviant behavior for those who already possess the related deviant cognition (Ajzen & Fishbein, 1980; Bosco et al., 2015; Wolfowicz, Weisburd, et al., 2021a).

Several lessons can be learned from the collective results from the literature. First, the magnitudes of the effects between television and video games are highly similar, even though the mediums are fundamentally and qualitatively different. While television offers a one‐way, passive form of exposure, videogames offer a two‐way passive‐active form of exposure. Secondly, the magnitudes of the effects for most media‐related risk factors are considerably smaller than analogous risk factors from the offline domain, such as social learning factors relating to exposure to violence, and having deviant associations. Third, the magnitudes of the effects for media‐related risk factors do differ based on the nature of content. This indicates that mere exposure to or usage of a medium is not sufficient. Rather it is the mediated exposure to specific types of content across different mediums that increases risk of deviant outcomes to varying degrees (Anderson & Dill, 2000; Anderson et al., 2007; Coyne et al., 2018; Gentile et al., 2004; Martins & Weaver, 2019).

### 2.4 Why it is important to do the review

As per the above, despite an extensive literature on media effects on aggression and other deviant outcomes, and in particular the extensive meta‐analytic contribution to this literature, to date there has been little movement to integrate the study of media‐effects on radicalization. Specifically, there have been few attempts to apply the theoretical and methodological frameworks used in the literature on media effects to this relatively new area of study (Scrivens et al., 2020). In response to this issue, the current study had the objective of integrating media effects on radicalization into the broader media effects literature as it pertains to criminal and criminal analogous outcomes more generally.

## 3 OBJECTIVES

This systematic review sought to first identify what the different media‐related risk factors for radicalization are, for which quantitative estimates exist. By organizing the identified factors in a rank‐order according to their pooled effect sizes, we sought to identify the relative magnitude of the estimates and contrast them between the different outcomes of radicalization. We also sought to explore whether heterogeneity can be explained by the different context and settings in which studies are carried out. The review addressed these objectives by seeking to answer four central research questions:

1) What are the different media‐related risk factors for cognitive radicalization?
2) What are the different media‐related risk factors for behavioral radicalization?
3) What are the relative magnitudes of the effect sizes for the different risk factors across the different outcomes?
4) Can heterogeneity within the strength of relationships be explained by differences between ideologies (e.g., right‐wing and Islamist inspired), and regions (e.g., EU and the US)?

## 4 METHODS

The methods for this review were pre‐determined in a systematic review protocol published in the Campbell Collaboration journal (Wolfowicz et al., 2021a). Below we re‐iterate the inclusion and exclusion criteria and the methods used in this review.

### 4.1 Criteria for considering studies for this review

#### 4.1.1 Types of studies

The review sought to identify, collate, and synthesize observational studies, namely cross‐sectional, longitudinal, and case‐control designs. The outcomes from these studies were expected to be derived primarily from self‐reports. However, the review also sought to include studies based on clinical reports (e.g., practitioner coded data), administrative data (e.g., from security services), and secondary databases constructed from multiple open sources (e.g., Profiles of Individual Radicalization in the United States [PIRUS]).

Experimental studies were included when the experimental manipulation involved some variation (e.g., at least two conditions) of some form of human‐media interaction, such as exposure to news‐media, videos. That is, the experimental condition differed in terms of the type of media that participants were exposure to, or the contents of the media that participants were exposed to assess differences in one of the eligible outcomes. This included lab‐based experiments, Internet‐based experiments, and experiments using vignette designs. For these types of studies to be included, the treatment or control must have included at least one condition in which the exposure was theorized to increase the likelihood of radicalization. These criteria also served to distinguish the studies and this review from research (including systematic review) on counter‐narratives (e.g., Carthy et al., 2020). While these studies' outcomes were also expected to be derived primarily from self‐reports, they were also open to inclusion if based on clinical reports or observations (e.g., practitioner coded data). These studies were eligible for inclusion irrespective of the method of assignment (e.g., random, random blocked).

For all study types, it was necessary for them to display variation on the dependent variable, (e.g., a proportion of the sample displayed, or didn't display the outcome of interest in the case of dichotomous measures or displayed it to varying degrees in the case of ordinal, discrete or continuous measures).

Cross‐sectional and longitudinal studies were included without any limitations on the nature of the sample (e.g., gender or age composition) or sampling method (e.g., Random, Representative, Quota, Convenience, Purposive, Snowball etc.).

For case‐control designs, studies were included when the “case” sample was made up of a group of individuals who displayed one or more of the outcomes of interest (see below “Types of outcome measures”) as assessed by self‐reports, clinical reports, or administrative decisions (such as a clear violation of the law in line with the outcomes of interest described below). For these studies to have been included, it was necessary that the control sample would be made up of one of the following types of individuals:

1) Individuals assessed for or who were suspected of having displayed one of the outcomes of interest but who were found not to, or who were found to display it to a lesser degree than the “case” sample or some chosen threshold.
2) Individuals from the general population not displaying the outcome of interest or displaying the outcome of interest to varying degrees that is representative of the natural distribution in the general population.
3) Individuals not displaying the outcome of interest but who display a related, excluded outcome (see below “Types of outcome measures”).

For these studies, controls could be chosen based on having been part of a single cohort (as in the case of prospective studies) or derived from a relevant data set (such as administrative records). Studies were also eligible for inclusion if the matched controls were derived from a separate, related data set, as is often used in retrospective studies. Studies were also eligible for inclusion when the control sample was derived from the general population and included individuals considered to be at risk for displaying the outcome of interest specifically, or a distribution of individuals displaying or not displaying (or displaying to varying degrees) the outcome of interest.

For longitudinal studies, we included both prospective or retrospective studies, as well as whether they are cohort or panel studies, that measured the indicator and outcome of interest at least two different time points. We set no limits on the time between measurement but expected it to be a period of at least a few months for a study to be considered to be a true longitudinal design. For cohort studies, whether prospective or retrospective, we included studies in which the cohort had some shared characteristic, such as being from a specific age group, geographic setting, or social setting at the time of selection. For panel studies, whether prospective or retrospective, we included studies irrespective of their sampling (e.g., Random, Representative etc.).

Observational studies and experimental studies will be treated separately in the analysis.

#### 4.1.2 Types of participants

Previous reviews of the literature have highlighted the dearth of research concerning the media‐radicalization relationship at the individual level. It has been found that most of the research focuses on the Internet, and specifically on platforms, content, groups, and networks. Whilst important, this type of research represents a distinct line of inquiry and is better described as the analysis of “online extremism” rather than “radicalization” (Odag et al., 2019). This type of research does not provide the type of information—due to the units of analysis—that can inform us about the media effects on radicalization (Winter et al., 2020).

As such, given that previous reviews have already dealt with this distinct line of inquiry, the current review sought to address the gap in the literature concerning the individual level. The review therefore included studies in which the participants, or units of analysis were individuals. The review therefore excluded studies where the unit of analysis was some form of media content, network(s), group(s), or other units of analysis that were unable to provide information pertaining to this review's research questions and objectives.

The review placed no restrictions on inclusion based on the individual characteristics of the participants. The review included all samples regardless of the participants' age, gender, ethnicities, religion, or the type of radicalizing doctrine being investigated (e.g., right‐wing, left‐wing, religious etc.). Previous systematic reviews have found that such factors rarely impact the pooled estimates in meta‐analyses of indicators pertaining to the same radicalization outcomes taken up by the current review (Wolfowicz et al., 2020). As such, while we combined studies examining the same indicators into a single analysis regardless of conceptual differences between the participants, where possible we explored participant population factors as possible sources of heterogeneity using meta‐regression, and moderator analysis (see section on “Assessment of Heterogeneity”).

#### 4.1.3 Types of factors

The review sought to include studies that examine at least one media‐related factor as an independent variable that provides an indication of correlation with one of the outcomes of interest. A media‐related factor was considered to be an exposure or experiential based indicator whose source is in some form of media. For example, a known risk factor for radicalization is exposure to violence (Wolfowicz et al., 2020). This review considered in‐person exposure to violence to be excluded but mediated exposure to violence (through television, social media, or other forms of media) would be an eligible indicator. In line with the review's objectives and research questions, the review made no predeterminations as to what media‐related factors may exist. Nevertheless, in Table 1 we provide some examples of the types of factors that were expected to be identified.

**Table 1:** Examples of types of factors expected.

Variable	Description
Passive consumption	Watching/reading/listening of mediated content
Active consumption	Posting/creating/engaging in mediated content
Frequency of use	Television/radio/music/print media/Internet/social media
Network characteristics	Individual (ego) network size/tie‐strength/density etc.
Differential associations	Online deviant peers/network members
User‐level behaviors	Likes/comments/shares/types of posts

#### 4.1.4 Types of outcome measures

**Figure 1:**
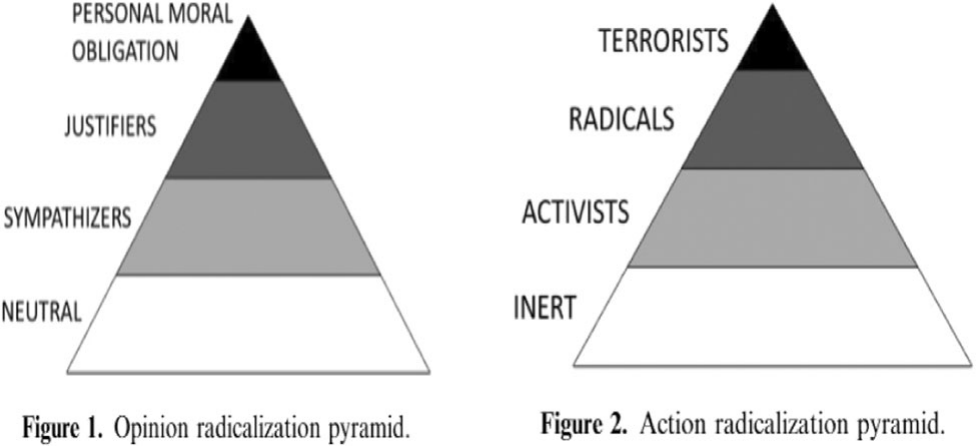
Two‐pyramid model (McCauley & Moskalenko, 2017).

In line with the literature and previous systematic reviews (e.g., Wolfowicz, Weisburd, et al., 2021b), this review examined two distinct, albeit interrelated outcomes, namely the cognitive and behavioral outcomes of radicalization. To ensure that the review included studies with comparable outcomes, the review limited its inclusion to studies whose dependent variables were assessed to be in line with the outcomes of McCauley and Moskalenko's (2017) Two‐Pyramid Model of radicalization (TPM). Broadly speaking, the TPM was selected because rather than being a traditional process models of radicalization, it is an outcome‐based typological model. The TPM differentiates between the cognitive (attitudinal) and behavioral outcomes of radicalization, and also distinguishes between legitimate, legal forms of activism that are often incorrectly conflated with radicalization, and the types of illegal, radical behaviors that are of concern to society and authorities (Moskalenko & McCauley, 2009). The TPM's typologies provide a clear set of criteria for determining if an outcome falls in to one of its categories, as well as for the categorization of the outcome (Wolfowicz et al., 2020). Moreover, when studying related cognitive and behavioral outcomes in parallel, the cognitive outcome should have a high level of specificity with reference to the behavioral outcome (Fishbein & Ajzen, 1975). This criterion is met by the TPM's typologies as described below (Figure 1).

In line with the TPM, studies were classified as examining either cognitive or behavioral radicalization outcomes, with each of these outcomes including two sub‐categories. With respect to cognitive radicalization the review included studies that examined radical attitudes or intentions. Studies examining radical attitudes were included when the dependent variable assessed support for, or justification of the use of radical violence, which is the use of violence toward persons in the name of a cause. This definition strikes a good balance between specificity and sensitivity, leaving room for the inclusion of violence motivated by a range of ideologies, and excluding non‐ideological forms of violence. Studies examining radical intentions were included when the dependent variable assessed intentions toward engaging in, or an expression of a willingness or desire toward the commission of radical violence. In both cases, studies were included irrespective of their use of single or multiple item outcome measures, if they included specific references to radical violence generally, or specific instances of radical violence. That is, while the review placed no specific restrictions on which measures were used, inclusion was limited to studies whose measures included such explicit reference to the use of some form of radical violence in the name of a cause.

While some reviews examining a broad, and large number of studies have previously assessed the relationship between individual factors radical attitudes and intentions separately (Wolfowicz, Weisburd, et al., 2021a), others have found it useful to combine them as a single measure of cognitive radicalization (Emmelkamp et al., 2020). Given the high degree of similarity in measurements used, and the fact that we did not expect as large a number of studies as has been found in previous reviews (e.g., Wolfowicz, Weisburd, et al., 2021a), we grouped studies examining radical attitudes and intentions together as a single outcome for cognitive radicalization. When a study reported on two separate outcomes for cognitive radicalization (e.g., radical attitudes and radical intentions), two separate analyses were conducted in which alternative effect sizes were used. Alternatively, when two or more studies contributing to a particular analysis reported on two or more outcomes, sub‐group analysis was conducted. Where the analysis for any particular factor included effect sizes derived from at least two studies for each of the outcome categories (attitudes and intentions), moderator analysis was performed to assess the degree of between outcome measurement heterogeneity (see “Sub‐group analyses and investigation of heterogeneity”).

With respect to behavioral radicalization, this review included studies that examined engagement in radical behaviors, which are sub‐terroristic forms of illegal, radical violence (to be distinguished from legal, non‐violent forms of activism), as well as studies that examined involvement in terrorism. Regarding radical violence, the review included studies that assessed self‐reports of prior engagements in radical violence (e.g., Pauwels & Schils, 2016), as well as studies based on administrative data, or databases derived from both official and open sources. Regarding terrorism, the review considered terrorism to be any form of terrorism offending (planned or successful terrorism offending) as defined by the law of the country in which the offence occurred. The review also included studies whose samples were drawn from data from official and open sources (e.g., PIRUS). Studies examining both self‐reported forms of radical violence and terrorism involvement were combined as a single outcome of behavioral radicalization. It was determined that in a case in which there were more than two studies from each of these categories in a single analysis, moderator analysis would be used to assess whether any between‐outcome measurement heterogeneity exists (see “Sub‐group analyses and investigation of heterogeneity”).

#### 4.1.5 Duration of follow‐up

While no limitations were placed on the duration of follow‐up, it was determined that if a sufficient number of longitudinal studies would be identified, they would be grouped together by the time interval to follow up. We considered the length of time between two time points from 0 to 12 months as a short‐term follow up period, 12–24 months as a midterm follow‐up period, and 24> months as a long‐term follow up period. It was determined that if a sufficient number of such studies were identified, a separate analysis would be conducted for each of the time point categories.

#### 4.1.6 Types of settings

No limitations were placed on the settings from which the samples of studies originated. Where possible, moderator analysis was used to identify differences in effects between different countries or regional settings.

### 4.2 Search methods for identification of studies

#### 4.2.1 Electronic searches

Pilot searches were conducted to identify the most appropriate combination of search terms and strings that would achieve the optimal balance between sensitivity and specificity. To be able to properly address our first research question, we excluded from the search terms any reference to specific indicators/independent variables (e.g., television), so as to not to pre‐determine or bias what factors would figure in the review (Shenderovich et al., 2016; Wolfowicz et al., 2020). Rather, in their place, we utilized general terms to capture all forms of media. At the recommendation of the editors from the Campbell Collaboration, who provided their input on the search strategy, we did include direct references to the Internet, as researchers are less likely to use terms like “media” to refer to the Internet. The search string also sought to ensure that the search results would be limited, as much as possible, to quantitative studies. During the revision process, searches were re‐conducted using the revised search string:

*Abstract/Title/Keywords* = (radical* OR extrem* OR terror* OR action OR “politically motivated*” OR ideological*) AND (media OR technology OR internet OR online) AND (quantitative OR empirical OR survey OR regression OR multivariate OR correlation OR experiment* OR manipulat* OR coefficient* OR covariat* OR longitudinal OR paramet* OR predict* OR questionnaire* OR sampl* OR standard deviation* OR statistic* OR variable* OR variance)

Searches were carried out in the following databases in EbscoHost: ERIC, Criminal justice abstracts, Political Science Complete, Violence and Abuse abstract, and Open dissertations. In addition, searches were carried out in the following databases: PsychInfo (PsychNet), Worldwide Political Science Abstracts (ProQuest), Social Sciences Citation Index (Web of Science), and PubMed.

While the original protocol stated that searches would be carried out in the International Security & Counter‐Terrorism Reference Center database as well as International Political Science Abstracts (IPSA), we were unable to access these databases. We opted to include the Worldwide Political Science Abstracts database, which was not included in the original protocol, given that it covers the IPSA.

Searches were carried out in August 2020 and were run again in March 2022 before publication of the review as part of the review process.

#### 4.2.2 Searching other resources

Given that the topic of radicalization is known to be addressed by a range of disciplines (Wolfowicz et al., 2020), searches were carried out in several different sources. An initial search was carried out on the Campbell Collaboration and Cochrane libraries. We also carried out searches to identify existing reviews and screen their reference lists to identify possible studies that met the inclusion criteria. While the searches were being carried out, we also contacted several recognized experts (researchers and organizations) and presented them with our review topic, criteria, and initial results, with a request that they direct us to any missing studies that they may be familiar with that could meet the inclusion criteria. For included studies, which were subjected to full‐text screening, we also reviewed their literature reviews and reference lists to identify whether they include any additional studies that may meet the inclusion criteria.

### 4.3 Data collection and analysis

#### 4.3.1 Selection of studies

After searches were performed, all search results were downloaded into Endnote X9 and stored in a shared library that all reviewers and research assistants had access to. A double screening process was implemented in which two reviewers screened the titles and abstracts of retrieved items to assess whether they related in any way to the topics of interest. The screening was performed directly in Endnote, where the reference window displayed the titles and abstracts. All studies considered to potentially meet the inclusion criteria were copied to a separate Endnote folder entitled “First screening.” The second stage of this process included a more thorough reading of the abstracts of selected studies to identify whether they were likely to provide quantitative information, and whether they assessed an outcome that is in line with the topic of interest. Studies selected at this stage were copied to a separate Endnote folder entitled “Second screening.” We then identified and downloaded the full text PDFs for each of the items in this folder and attached them to their respective references within Endnote. Subsequently, the full‐text PDFs were accessed and assessed for inclusion based on readings of the methods sections, with particular attention given to the sections describing the measurement of the outcomes and indicators, as well as the nature of the sample and methods used. Only studies meeting all inclusion criteria were copied to a sub‐folder entitled “Final inclusion.” Screening decisions were made by two reviewers. Final inclusion decisions were compared, and any discrepancies were reconciled in a joint meeting between the reviewers.

#### 4.3.2 Data extraction and management

In addition to effect sizes, we also coded several study‐level characteristics that are known to potentially impact heterogeneity and thereby affect the results or the way in which they should be interpreted. Where possible, we examined the effects of study level characteristics using meta‐regression and moderator analysis. Since there is no consensus as to the minimum number of studies needed for conducting such analysis, we adopted a minimalist's approach in which the minimum number of studies needed for examining a continuous variable is 6 (Fu et al., 2011) and 2 studies per category for categorical variables (Marino et al., 2018). Rather than using meta‐regression to justify the use of moderator analysis as originally determined in the protocol, we carried out moderator analysis for all factors for which a sufficient number of effect sizes existed. Table 2 includes the different characteristics that were coded.

**Table 2:** Study‐level characteristics.

Variable	Description
Effect size derivation	Categorical: Effect size from bivariate or partial effect size.
Age	Continuous: Mean age of the sample
Gender composition	Continuous: Proportion of the sample that is male
Year	Continuous: Last year of data collection
Ideology	Categorical: Right‐wing, left‐wing, religious etc.
Region	Categorical: US, EU, other.
Measurement	Continuous: The number of components in a measurement
Publication status	Dichotomous: Published/unpublished
Sample type	Categorical: Random/representative/convenience etc.
Language	Categorical: Language that the publication was written in
Study design	Categorical: Cross‐sectional, longitudinal, case‐control etc.

All coding was performed by two coders, one of whom is the first author. Coding was carried out using Excel. At the end of the coding stage, coder inter‐reliability was assessed. In all instances in which the coding of effect sizes differed between the coders, the coders re‐coded the data and conducted the analysis jointly. Similarly, the coding of study‐level characteristics was coded by the same two coders. Identified differences were rectified through a joint re‐coding.

#### 4.3.3 Assessment of risk of bias in included studies

While the review sets few limitations on inclusion based on study quality, several measures of study quality will be coded as study‐level characteristics to assess risk of bias. Where possible, we will attempt to analyze how these factors may impact the results using meta‐regression. Where this is not possible, the risk of bias elements of studies will be raised in the discussion of the results. For observational studies, we will carry out the assessment of risk based on the most relevant items derived from the ROBINS‐E (Risk‐of‐bias in non‐randomized studies of exposure) assessment tool. The selected risk of bias items that will be assessed are listed below in Table 3.

**Table 3:** Risk of bias measures for observational studies.

Variable	Description
Dependent variable(s)	Was the dependent variable measured using a validated instrument?
Independent variable(s)	Was exposure to the independent variable measured using a validated instrument?
Measurement source	Was the source of the measurement for the independent/dependent variable based on self‐reported, clinician reported, administrative reported, or other type of data?
Sampling methodology	Did the study use Systematic, stratified, convenience, quota, purposive, snowball, or another type of sampling?
Temporal ordering	Can temporal ordering be established between the independent and dependent variable(s)?
Analytical rigor	Did the statistical models used adequately control for key confounding variables?
Missing data	Was missing data an issue in the study, and if so, how was it dealt with?
Results	Were results reported in a non‐biased way (e.g., were non‐significant results also reported)?
Reporting bias	Does the study indicate that it has the data to report on a key relationship but fails to do so?
Outcome bias	Does the study indicate that it has the data to report on a relationship with an alternative outcome but fails to do so?

In the ROBINS‐E guidance, overall ratings for risk of bias are determined by the highest risk of bias rating in each individual domain. This means that all domains essentially contribute equally to the overall judgment of a study. It has been noted that one of the limitations of this approach is that studies with high risk of bias in only one domain may be assessed to be of equally low quality as studies with high risk of bias in multiple domains. As such, we report the full risk of bias assessment so that users can easily see the different sources of bias that were identified in the included studies (Bero et al., 2018).

For randomized experimental studies, we used an adapted version of Cochrane's Risk of Bias tool for randomized trials (RoB2). As with the observational studies, risk of bias relates to both the study and outcome levels, but there are two additional risk domains that differentiate experimental from non‐experimental studies, namely: Domain 1 (risk of bias arising from the randomization or other selection process) and Domain 2 (risk of bias due to deviations from the intended interventions). The other domains of the RoB2 tool are analogous to the domains for the observational studies, namely: Domain 3: Risk of bias due to missing outcome data; Domain 4: Risk of bias in measurement of the outcome, and Domain 5 (risk of bias in the selection of the reported result). Given the nature of the literature, certain items in the tool were not relevant and as such, a more compact version of the tool was adopted (e.g. Lum et al., 2020). The following items from RoB2 were included (Table 4).

**Table 4:** Risk of Bias measures for experimental studies.

Item	Description
1.1	Was the allocation sequence random?
1.3	Did baseline differences between intervention groups suggest a problem with the randomization process?
2.6	Was an appropriate analysis used to estimate the effect of assignment to intervention?
2.7	Was there potential for a substantial impact (on the result) of the failure to analyze participants in the group to which they were randomized?
5.1	Were the data that produced this result [the results for this study] analyzed in accordance with a pre‐specified analysis plan that was finalized before unblinded outcome data were available for analysis?
5.2	Is (Are) the numerical result [results] being assessed likely to have been selected, on the basis of the results, from multiple outcome measurements?
5.3	Is (Are) the numerical result [results] being assessed likely to have been selected, on the basis of the results, from multiple analyses of the data?

Similar to the observational studies, experimental studies were assessed as being of low, high, or unclear risk of bias overall according to the highest assessment from any single domain. That is, if a study was assessed to have high risk of bias in one domain, we classify the entire study as being at high risk of bias.

#### 4.3.4 Measures of effects

There is a lack of consensus as to whether meta‐analysis based on observational studies should give preference to effect sizes derived from bivariate correlations, or correlations derived from the results of multivariate regression models that have controlled for confounders. While standardized coefficients derived from multivariate models may produce estimates closer to the “true” value, there is significant between‐study variations in model specifications, and studies are often not comparable (Hanushek & Jackson, 1977; Hunter & Schmidt, 2004). For this reason, many researchers give preference to bivariate correlations, which offer a consistent and uncontaminated measure across studies (Pratt et al., 2014). The decision whether to use bivariate, multivariate, or a combination of these effect sizes also depends on a study's objectives (Aloe et al., 2016). One of our objectives was to identify the relative magnitude of the effects for different media‐related factors, and bivariate correlations provide more stable estimates for developing a rank‐order of estimates among multiple factors (Hedges & Olkin, 1985). As such, we follow the approach that gives preference to bivariate correlations but allows for the inclusion of effect sizes standardized from regression models as supplementary effect sizes when that is all that is available. We will use moderator analyses to assess and report the effects of this approach (Aloe et al., 2016). We believe that this approach is the one most suited to the current review. It is also in line with related works that have been conducted (e.g., Najaka et al., 2001; Wong et al., 2010), and serves to enable the inclusion of the greatest possible number of effect sizes (Borenstein et al., 2009).

All effect sizes were standardized as Fisher's *Z* transformed variables (Borenstein et al., 2009; Rosenthal, 1984), which was also used for reporting of results. Effect sizes were derived from bivariate correlations, primarily from correlation matrices, or calculated from descriptive data such as means and standard deviations, *t* tests, *χ* ^2^, analysis of variances (ANOVAs), and other classical hypothesis tests. The calculation of bivariate effect sizes from descriptive data was carried out using the formulas and conventions of Lipsey and Wilson (2001), and the “Practical Meta‐Analysis Effect Size Calculator” available through the Campbell Collaboration website.

For effect sizes derived from bivariate sources, standard errors were calculated based on the variance of the *z* transformed variable (1/*n* − 3), in which the standard error is calculated as being the square root of the variance.

Where a study reported only the results from linear regression models where the independent variable (IV) and dependent variable (DV) are both continuous we calculated *r* as

$$r=\frac{SD_{x}B}{SD_{y}}.$$

In situations in which standard deviations (SD) were not reported, or when the IV was dichotomous and the DV continuous, or where the IV was ordinal or continuous and the DV was dichotomous, *r* was calculated based on the *t* ratio (*B*/SE):

$$r=t/\sqrt{t^{2}\;+\;}n-2.$$

In instances in which both IV and DV were dichotomous, and only *B* was reported we first calculated Cohen's *d* and then converted this to *r* as follows:

$$d=B\left(\frac{\sqrt{3}}{\pi }\right),$$

$$r=\frac{d}{\sqrt{4+d^{2}}}.$$

For these conversions, standard errors were calculated, whenever possible, based on a rescaling of the model‐based standard error, which is calculated as

$${se}_{r}=\frac{r\times SE_{B}}{B}.$$

When the SE was not reported, it was calculated from reported confidence intervals (CIs). In the case of 95% CIs, the SE was calculated as

$$SE=\frac{{\mathrm{CI}}_{\mathrm{upper}}-{\mathrm{CI}}_{\mathrm{lower}}}{1.96}.$$

For experimental studies, while it is common to calculate the standard mean difference, Cohen's *d*, there is a tendency for overestimation of effects in small samples (Hedges, 1981). As such, we determined to use Hedge's *g*, which corrects for this bias. To calculate Hedge's *g*, we would first calculate the correction factor, *j* as

$$J=1-\frac{3}{4\mathrm{df}-1}.$$

With Cohen's *d* already having been calculated based on the means and standard deviations of the groups or other summary statistics, we calculate *g *=* J *× *d*. Subsequently we calculate the standard error as the square root of *V*_*g*_, with *V_g_* calculated as

$$V_{g}=J^{2}\times V_{d}.$$

#### 4.3.5 Dealing with missing data

When a study was missing information pertaining to study level characteristics or effect sizes, the following actions were taken in the order in which they appear:

1. Search for supplementary materials.
2. Search for access to the original data and replicate the model.
3. Search for other studies by the authors that may use the same data.
4. Search for studies by other authors that may use the same data.
5. Contact the authors with a request to provide the missing data.

It was originally determined that in a case in which a study reported on the effect of a particular factor as not having been statistically significant and no additional information was given or had been acquired through the above steps, an effect size of zero would be entered into the meta‐analysis (Durlak & Lipsey, 1991). Additionally, it was determined that if a study presented quantitative findings that were unable to be synthesized in the meta‐analysis, because the statistic presented does not enable conversion or standardization, or there is not enough information available, the above steps would be followed.

#### 4.3.6 Assessment of heterogeneity

Heterogeneity was assessed using Cochran's *Q* and its associated *p* value, as well as the *I* ^2^ statistics. *I* ^2^ scores of >75 indicate high, >50 moderate, >25 medium heterogeneity, and >0 low heterogeneity. When *I* ^2^ = 0 it indicates an absence of heterogeneity.

#### 4.3.7 Assessment of reporting biases

##### 4.3.7.1 Between studies

Reporting bias is a known issue with meta‐analytic studies. Commonly referred to as the 'file‐drawer problem', it is widely understood that researchers tend to avoid publishing non‐significant results, or occasionally results with depicting exceptionally small effects. As a result, the identifiable literature may not be representative of all the studies that have been conducted on a given issue, and the results from pooling these studies may overestimate the true effect size (Rosenthal, 1979). In this review two methods were used to assess reporting bias.

While the published protocol stated that we would use Rosenthal's (1979) Fail‐Safe *N* test, based on comments received, as well as the approach taken in previous reviews (Wolfowicz, Weisburd, et al., 2021a), we replaced this approach with Egger's regression test (Egger et al., 1997). In Egger's regression test, the standardized effect sizes are regressed on their precisions. This is the equivalent of a weighted regression of the effect sizes on their standard errors, in which weighting is on the inverse variance. In the absence of publication bias, the intercept is expected to be zero (Rothstein et al., 2006). A statistically significant intercept above zero indicates the presence of publication bias.

The second approach noted in the protocol was maintained, namely the Trim‐and‐fill method (Duval, 2005; Duval & Tweedie, 2000a, 2000b). This method estimates the number of studies missing on the extremities of a funnel plot and augments the observed data to create a more symmetric distribution. Based on this, adjusted estimates and heterogeneity statistics are generated and enable the assessment of the degree to which the results are sensitive to reporting bias.

As both methods suffer from their own limitations (Egger et al., 2001; Sterne et al., 2000), they have often been used complimentarily, including both in risk factor related research (e.g., Assink et al., 2015; Vazsonyi et al., 2017; Wolfowicz, Weisburd, et al., 2021a).

##### 4.3.7.2 Within studies

A common issue in different literatures concerns outcome reporting bias, in which studies may not report on the relationship between variables and certain outcomes even when it is clear that they have enough information to report on such relationships. In some cases, a study may make no mention of possible alternative outcomes, even though the researchers have collected data pertaining to it. To assess and account for outcome reporting bias, we will search for online supplementary materials and open access data for all included studies to identify any additional sources of reporting bias as they pertain to unreported outcomes. For studies for which supplementary materials or original data is found, we will code and report as to whether such materials provide evidence to support the identification of either of the two types of reporting bias described above.

#### 4.3.8 Data synthesis

Meta‐analysis was conducted for each factor for which at least two unique effect sizes were found. All analyses were carried out using Biostat's Comprehensive Meta‐Analysis (CMA) software (Borenstein et al., 2009). Random effects models were used for all analyses to account for heterogeneity. CMA V3 uses the Method of Moments approach of DerSimonian and Laird (1986) as the random effects estimator for *τ* ^2^. Results were arranged in rank order according to the size of the pooled estimates for the different factors analyzed.

#### 4.3.9 Sub‐group analysis and investigation of heterogeneity

To further investigate and understand sources of heterogeneity, and to identify ways in which estimates for different factors may fluctuate between different contexts and conditions, a number of additional analyses were planned.

As described above, meta‐regression was used to assess the effects of continuous variables, and moderator analysis was used to assess the effects of categorical variables. The following variables were examined:

- Region from which the sample was derived (EU, US, and Other).
- Ideological strain examined by the study (non‐specific/mixed ideologies, Right‐wing, Left‐wing, Islamist, and Other (which included separatist and ethno‐nationalist).
- Year of data collection.
- Mean age of study sample.
- Proportion of males in study sample.

We adopted a minimalist approach in which a minimum of two studies from each category is needed to perform meta‐regressions for categorical variables, and a minimum of six studies is needed for continuous variables. For categorical variables, wherever the meta‐regression provides a statistically significant effect (*p* < 0.10), moderator analyses will be used to assess the degree of between category heterogeneity and differences in the estimates.

As our approach was to identify and utilize bivariate correlations as our preferred effect size, and only utilize effect sizes derived from multivariate models when bivariate calculations were not possible, we did not assess differences in the effect sizes from different studies based on the other (both included and excluded) covariates in a study. However, for any analysis for any factor that included at least two effect sizes derived from different sources (Bivariate or standardized partial effect sizes), we assessed the impact of combining the effect sizes through moderator analysis. The moderator analysis was used to identify the degree of between category heterogeneity and differences in the estimates across the categories. As per the above, we did not carry out meta‐regressions as a justification for this analysis as was originally considered in the protocol, as it was determined that this was an unnecessary step.

#### 4.3.10 Sensitivity analysis

Sensitivity analysis was carried out for each analysis that included 3 or more studies by using the “leave‐one‐out” method. In this method the meta‐analysis is iteratively repeated *k* times (*k* = the number of studies), with a different study excluded at each iteration and the estimates and heterogeneity statistics re‐calculated. The results provide for the ability to examine whether a specific study is responsible for greatly influencing the results (Viechtbauer & Cheung, 2010). We report on those analyses in which the removal of a single study was found to reduce heterogeneity by at least one level, with the levels being low (*I* ^2^ < 25), moderate (*I* ^2^ < 50), high (*I* ^2^ < 75), and very high (*I* ^2^ > 75).

## 5 RESULTS

### 5.1 Description of studies

#### 5.1.1 Results of the search

Our initial systematic searches were carried out in August 2020 and resulted in the retrieval of 36,732 unique items which were subsequently transferred to an EndNote library. Once imported to the library and fully updated, an initial screen was carried out based on their titles and abstracts. The initial screening sought to remove all studies that were clearly not related to the general topic of radicalization (or associated terms) in any way (such as studies from medicine examining “free radicals,” environmental studies examining “extreme” weather, and studies examining more closely related topics such as fear of terrorism, or the impact of terrorism on different markets). In addition, all duplicates were removed at this stage. Following this process, we were left with 569 items, which together with 4 items we received from experts, left 573 items to be reviewed. The second screening stage included a more careful reading of the abstracts to identify whether studies dealt with a topic related to the current review. In some cases, where the abstracts were not clear as to what was being examined, a scanning of the methods sections of the papers was carried out. This process led to the removal of a further 469 items, as most of these studies were found to be examining group, platform, or content level dynamics, or using proxies for radicalization that fell outside of the inclusion criteria, such as voting for populist right‐wing parties in democratic elections. For the remaining set of 104 studies, we downloaded the full‐text PDF for each item and attached them to their respective items in the EndNote library. From here, we reviewed the methodology sections of each item and identified whether the dependent variable(s) was in line with the inclusion criteria, and whether any of the independent variables measured a media‐related factor. The final inclusion was 41 studies. In addition to these studies, we included 1 study that was sent to us by an expert (others that were received did not meet the inclusion criteria), 4 studies that we were familiar with, and which did not appear in our searches, and 1 unpublished study by the review's authors, for a total of 47 included studies.^2^

In March 2022, following consultations with the editorial team of Campbell Collaboration's Crime and Justice Group, we re‐ran the searches using a slightly modified search syntax, limiting search results to those published before August 2020 when the initial searches were carried out. These updated searches resulted in the retrieval of an additional 11,191 unique items. Following the same process as the initial screening, 87 potentially relevant items were identified in the first screening. However, upon a more careful inspection of the titles and abstracts, 20 of these items were removed. Among the remaining 67 studies, we excluded *N *= 43 based on their outcome not meeting the inclusion criteria, *N = *4 for not having a relevant indicator variable, *N =* 6 for their unit of analysis (not individual level), and *N = *5 for being qualitative, and *N *= 1 for having an invalid control group.^3^ There were a total of four studies for which we were unable to locate a copy of the study online. After contacting the authors, two of these studies were provided to us, one of the authors failed to respond to our request, and another author (who was the sole author of the study) passed away some year ago.

While *N *= 6 studies met the inclusion criteria, *N* = 1 of them was the dissertation that preceded an already included study and did not include any new information (Luchsinger, 2018), and *N* = 2 others were published by the same author of an already included study (Pauwels & Schils, 2016) and did not provide any new information to be synthesized.

**Figure 2:**
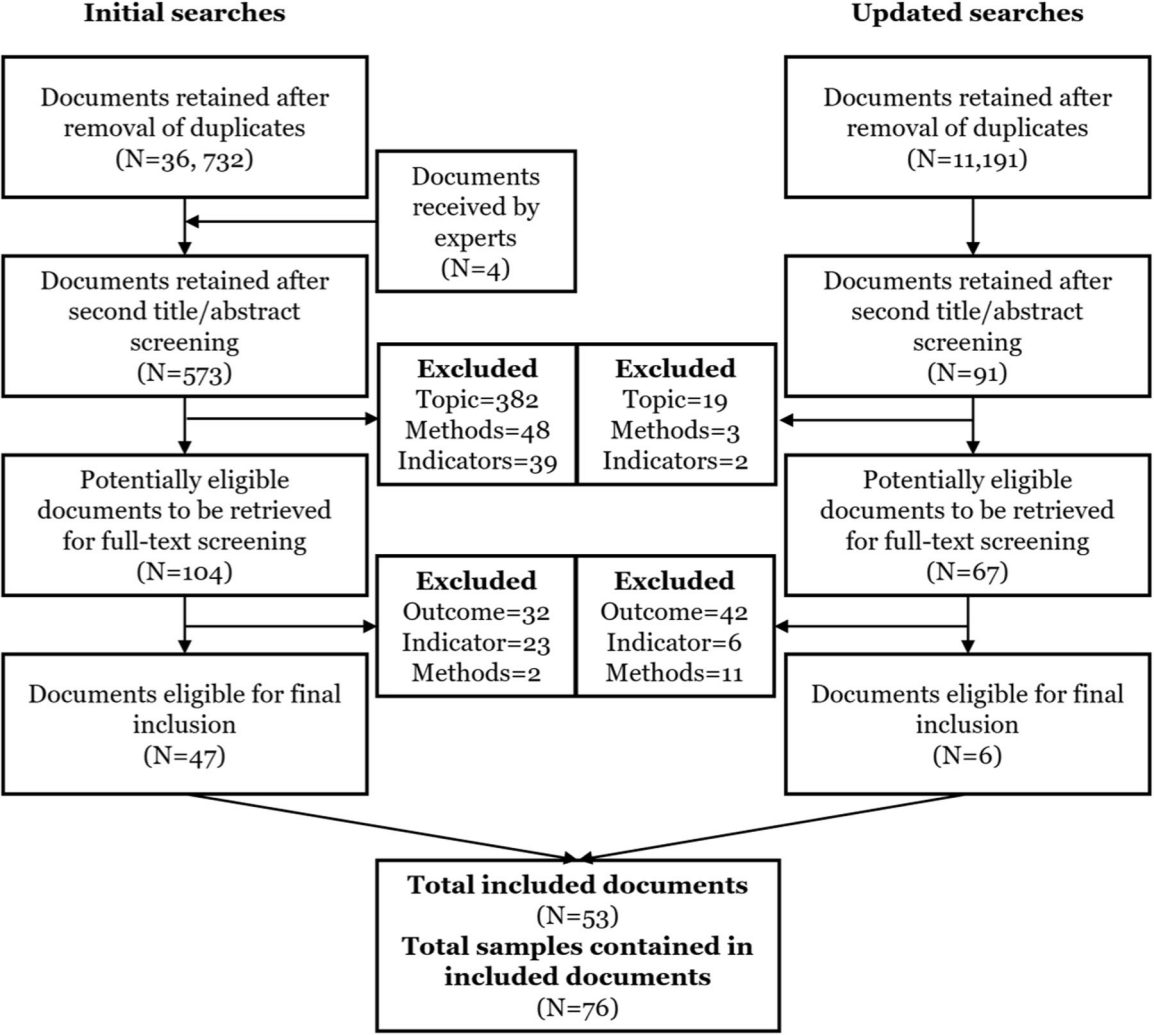
PRISMA flowchart.

In combining the results from the two sets of searches, the review had a final inclusion of *N = *53 publications containing *N =* 76 samples. The PRISMA flowchart below depicts the different stages of the searches and screening process (Figure 2).

#### 5.1.2 Included studies

Most studies were published from 2016 to 2020, with only a maximum of two studies appearing in years before this, going back to two studies published in 2002. The year with the largest number of studies was 2019, followed by 2020. Most of the studies were published in journals; however, the included items also included published reports, one book chapter, three theses, and one unpublished paper. Among the 76 samples included in 53 items, most were derived from European countries (32.9%), followed by North America (26.3%), Middle Eastern and African countries (25%), and East‐Asian countries (7.9%). There was an additional study which had a mixed sample of Europe and North America, as well as for North America and Asia. One study focused on a South American sample, and another on Australia. Table 5 provides a description of the included studies (more details are available in the Supporting Information Appendix).

**Table 5:** Descriptive information on included studies.

Study	*N*	Age	% Male	Ideology	Country	Outcome	Year of collection	Publication status
Azeem et al. (2019)		22.99	42.55					
Sample 1	200			Isl.	USA	C	NR	P
Sample 2	122			Isl.	Pakistan	C		
Baier (2010)	18631	NR	50	RW	Germany	C	2007	R
Berger (2016)								P
Sample 1	275	33	52	Isl.	UK	C	2006	"
Sample 2	374	32.97	49	Isl.	France	"	"	"
Sample 3	295	37.08	46	Isl.	Germany	"	"	"
Sample 4	270	32.93	77	Isl.	Spain	"	"	"
Bhatia and Ghanem (2017)	6979	NR	NR	Isl.	Mixed ME	C	2012	R
Bhui et al. (2016)	608	30	54	Isl.	UK	C	2013	P
Brunsting and Postmes (2002)	539	42	30.2	LW	Netherlands	C	NR	P
Calenda and Mosca (2007)						B		P
Sample 1	197	NR	37	LW	Italy	"	2001	"
Sample 2	197	NR	52	"	"	"	2002	"
Charkawi et al. (2020)	187	NR	47.59	Isl.	Australia	C	2017	P
Clemmow et al. (2020)	2233	30.23	48	Mix	Mixed OECD	B	NR	P
Ellis et al. (2016)	374	21.3	62.3	Isl.	US/Canada	C	2014	P
Ellis et al. (2019)	213	NA	100	Isl.	US/Canada	C	2015	P
Eyal et al. (2006)	446	21	31.6	Mix	US	C	NR	P
Fair and Patel (2019)	23361	37	51	Isl.	Mixed ME	C	2012	P
Fair and Savla (2019)								P
Sample 1	245	NR	67.35	Isl.	Cameroon	C	2009	"
Sample 2	339	"	58.11	"	Ghana	C	"	"
Sample 3	373	"	50	"	Guinea Bissau	C	"	"
Sample 4	279	"	48.53	"	Liberia	C	"	"
Frissen (2019)								T
Sample 1	2014	NR	51.9	Mix	Belgium	C	2018	"
Sample 2	1364	NR	28.7	Mix	Canada	C	2017	"
Frissen (2020)	1872	17.14	52.2	Mix	Belgium	C	NR	P
Frissen et al. (2019)	317	18.14	40.7	Mix	Belgium	C	NR	P
Gentzkow and Shapiro (2004)	1004	NR	NR	Isl.	Mixed ME	C	2002	P
Goede et al. (2019)	6715	14.7	47.4	Isl.	Germany	C	2018	R
Gvirsman et al. (2016)	1501	NR	NR	Mix	Israel	C	2010	P
Hawthorne (2016)								T
Sample 1	196	35.67	57.14	Mix	US	C	2016	
Sample 2	270	20.66	35.56	Mix	US	C	2016	
Holt and Kilger (2012)	353	22.29	60	Mix	US	C	2010	P
Holt et al. (2017)	779	NR	48	Mix	US/Taiwan	C	2011	P
Jones & Paris (2019)								P
Study 1	187	NR	34.8	Mix	US	C	2015	
Study 2	181	NR	56.4	Mix	US	C	2015	
Study 3	270	NR	38.15	Mix	US	C	2016	
Study 4	302	NR	37.75	Mix	US	C	2016	
Study 5	194	NR	42.5	Mix	US	C	2016	
Study 6	199	NR	47.74	Mix	US	C	2016	
Kalmoe (2014)								P
Study 1	412	NR	NR	Mix	US	C	2010	
Study 2	512	NR	NR	Mix	US	C	2010	
Study 3	384	27.3	49	Mix	US	C	2010	
Kaltenthaler et al. (2018)	1500	NR	50.3	Isl.	Iraq	C	2015	P
Kremerman et al. (2012)	284	20.23	45.4	Mix	Israel	C	NR	P
LaFree and Morris (2012)	3645	NR	54.7	Isl.	Mixed ME	C	2007	P
Lee (2018)	1012	NR	NR	Mix	Hong‐Kong	C	2016	P
Luchsinger (2018)	396	32.26	69.3	Isl.	US	C	2017	T
Luchsinger (2020)	396	32.26	69.3	Isl.	US	C	2017	P
Manzoni et al. (2019)		NR	49.7		Switzerland	C	2017	R
Sample 1	2932	"	NR	RW	"	"	"	"
Sample 2	5697	"	NR	LW	"	"	"	"
Sample 3	476	"	NR	Isl.	"	"	"	"
Mock (2014)		37	50					
Sample 1		NR	NR	Isl.	Egypt	C	2011	T
Sample 2		"	"	"	Indonesia	"	"	"
Sample 3		"	"	"	Jordan	"	"	"
Sample 4		"	"	"	Lebanon	"	"	"
Sample 5		"	"	"	Turkey	"	"	"
Mourão et al. (2016)	37102	37.84	49.6	Mix	Mixed SA	C	2012	P
Mulyana (2020)	100	NR	NR	Il.	Indonesia	C	NR	P
Nivette et al. (2017)	1214	17	50	Mix	Switzerland	C	2015	P
Pauwels and Schils (2016)	6020	20	35	Mix	Belgium	C/B	2013	P
Pauwels and Hardyns (2018)	6020	20	35	Mix	Belgium	C/B	2013	P
Pauwels et al. (2020)	6020	20	35	Mix	Belgium	C/B	2013	P
Pedersen et al. (2018)	7659	17.05	45	Mix	Norway	C	2015	P
Piazza and Guler (2019)	8400	39.97	49.9	Isl.	Mixed ME	C	2017	P
Sahani (2018)	347	33.48	92.2	Mix	US	B	2017	T
Schbley (2004)	2619	NR	NR	Isl.	Mixed EU	C	2003	P
Schröder et al. (2020)	4835	24.2	41.9	Mix	Germany	C	2019	R
Schumann et al. (2021)	880	NR	53.1	Isl.	UK	C	2015	P
Shortland et al. (2020)	1112	NR	82.46	Mix	US	C	NR	P
Sirgy et al. (2019)	32604	37.2	49.2	Isl.	Mixed ME	C	2012	P
Storm et al. (2020)	20439	17.17	47	Mix	Norway	C	2015	R
Tang et al. (2020)	290	NR	40.7	Mix	Hong‐Kong	C	2019	P
Wojcieszak (2010)	114	33	86	RW	US	C	2005	P
Wolfowicz et al. (2021b)	96	18.67	16	Isl.	Israel	C	2019	NP
Wong et al. (2019)	454	NR	NR	Mix	China	C	2016	P
Zhu et al. (2020)	216	20.03	56.9	Mix	Hong‐Kong	B	2016	P

*Note*: For Age, % Male, and year of publication NR = not reported. For ideology, Isl. = islamist, LW = left‐wing, RW = right‐wing, Mix = mixed or non‐specific ideology. For countries, Mixed SA = South America, Mixed ME = Middle East, Mixed EU = European, Mixed OECD = Democratic countries, US = United States of America, UK = United Kingdom. For outcome, C = cognitive radicalization, B = behavioral radicalization. For publication status, NP = not published, P = published journal article, R = report, T = thesis.

From experimental studies, we extracted 12 effect sizes that pertained to manipulated exposure to mediated content theorized to increase radicalization. We also extracted an additional 4 effect sizes pertaining to relevant interactions between treatment exposure and trait aggression. From observational studies, we identified 23 media‐related factors for which at least two effect sizes were identified, with a total of 110 effect sizes extracted for the primary analyses, and an additional 10 effect sizes extracted pertaining to sub‐groups. Table 6 provides a description of these factors, which are listed in alphabetical order.

**Table 6:** Description of media‐related factors included in meta‐analysis.

Factor	Description
Active exposure	Active engagement with/seeking out radical content
Cyber‐attack	Willingness to carry out ideologically motivated cyber attack
Cyberbullying	Being a victim of cyber‐bullying
Discussion forums	Participation in online discussion forums
Facebook	Frequency of Facebook usage
General follow politics	General Internet usage to follow politics
General media exposure	Exposure to mixed forms of media for news/violent content
Hacking	Engaged in ideologically motivated, unauthorized access to systems
Internet access	Access to the Internet
ISIS/Al‐Qaeda news	Follow ISIS/Al‐Qaeda new sources
Network attachment	Level of attachment/connectedness to the Internet/online network
Newspaper	Printed/online newspaper
Passive exposure	Passive exposure to radical content
Piracy	Illegal downloading of content from the Internet
Posting views	Posting political views/opinions on the Internet and social media
Radio	Listening to radio
Self‐censorship	Holding back sharing opinions
Tech‐skills	Technical and digital literacy
Television	Watching television
Time online	Time spent on the Internet
Trust in media	Trust in media/view media as biased
Twitter	Frequency of Twitter usage

While the descriptions for many of the factors are quite straightforward, some are deserving of further elaboration, particularly what we refer to as “radical content.” We consider “radical content” to be mediated content that is produced by individuals or groups with the goal of, or potential of increasing support or justification of terrorism (the use of violence in the name of a cause or ideology), or terrorist groups. This content usually makes use of violent images or messages which seek to generate sympathy or justification for the use of violence in defense or furtherance of an ideology or cause. For example, Frissen, (2019, 2020) examines passive exposure to, and the active seeking out of Jihadist information, which is measured by different types of materials, including: Dabiq (Islamic State's magazine), Inspire (Al‐Qaeda's magazine), jihadist videos, beheading videos, Salafi‐jihadist fora, and Salafi‐jihadist Facebook groups. Similarly, Manzoni et al's (2019) study assessed whether participants had visited websites, or viewed videos produced by well‐known radical groups, such as the Neo‐Nazi Blood & Honor, as well as Al‐Qaeda and ISIS. Wolfowicz's (2021b) study took a slightly different approach and examined whether participants had been exposed to violent content that they felt promoted violence against civilians and security forces. In the review, we distinguish between passive exposure to and the active seeking out of this type of radical content.

#### 5.1.3 Excluded studies

##### 5.1.3.1 Observational studies

Most of the observational studies that were excluded during the screening process failed to report on the relationship between a media‐related factor and an included outcome of radicalization. In most instances, studies were excluded for failing to meet both criteria, and not just one.

##### 5.1.3.2 Experimental studies

The search results identified several studies that referred to themselves explicitly as “experimental” or otherwise described themselves in a way that indicated that an experiment was carried out. However, following full‐text screenings it was identified that these studies could not be properly considered to be experimental due to the lack of a control group. For example, studies by Rieger et al. (2017, 2020) examined the effects of exposure to radical right‐wing and Islamist videos with a single sample pre/posttest design. Additionally, Frischlich et al. (2018) compared exposure to extremist content with exposure to counter‐narratives, thus lacking an acceptable control group. Glaser et al. (2002) compared exposure to different scenarios intended to induce out‐group anger. However, there was no neutral condition, and all participants were exposed to each of the conditions.

Other studies were excluded as it was identified that a media‐related factor was not in fact the treatment component of the study. For example, Reeve (2019) used a fictional extremist website as the exposure condition, in which all participants accessed the website and the treatment was a mortality salience prime, which was assessed for its effects on participants' interactions with the site. Lemieux and Asal (2010) used a vignette design in which participants were asked to take a first‐person perspective as a member of a fictional ethnic minority. Four different vignettes manipulated the degree of grievances associated with the different conditions. As per the study, the main independent variable was “grievances,” and no group was exposed to a neutral condition. As such, the study's treatment does not represent a form of included media‐exposure since it did not include a neutral condition. A similar approach was taken by Lemieux et al. (2017), and as such, this study was excluded on the same basis.

Additionally, some studies that otherwise would have met the inclusion criteria for being experimental studies examining an appropriate media‐related factor were excluded based on examining an outcome that failed to meet the inclusion criteria. For example, Shortland et al. (2021) examined a theoretical aggressive response to a car accident.

### 5.2 Risk of bias in included studies

The risk of bias assessment is contained in Table 7 (Justifications for risk of bias decisions can be found in the Supporting Information Appendix). As detailed below, the studies were generally of low quality and suffer from multiple sources of bias. As per the ROBINS‐E guidance, overall assessments of risk of bias are made based on the risk of bias rating for each individual domain. Therefore, while many studies are considered of low quality due to high risk of bias in a single domain, others are of even lower quality due to high risk of bias in multiple domains.

**Table 7:** Risk of bias assessment.

**Study**	*Dependent variable*	*Independent variable*	*Measurement source*	*Sampling method*	*Temporal ordering*	*Analytic rigor*	*Missing data*	*Results*	*Reporting bias*	*Outcome bias*	EXPERIMENTAL	*Random allocation*	*Baseline issues*	*Analytic rigor*	*Substantial impact*	*Pre‐determined analysis*	*Multiple outcomes*	*Multiple analyses*
Azeem et al. (2019)	N	N	Self	NR	N	Y	N	Y	N	N		NR	N	Y	Y	NR	N	N
Baier (2010)	N	N	Self	Stratified	N	Y	N	Y	N	N								
Berger (2016)	N	N	Self	Representative	N	Y	N	Y	N	N								
Bhatia and Ghanem (2017)	N	N	Self	Representative	N	Y	N	Y	N	N								
Bhui et al. (2016)	Y	N	Self	Representative	N	Y	N	Y	N	N								
Brunsting and Postmes (2002)	N	N	Self	Purposive	N	Y	Y	Y	N	N								
Calenda and Mosca (2007)	N	N	Self	Non‐probabilistic	N	Y	N	N	Y	N								
Charkawi et al. (2020)	N	N	Self	Random	N	Y	N	Y	N	N								
Clemmow et al. (2020)	Y	N	Self	Random	N	Y	N	Y	N	N								
Ellis et al. (2016)	Y	Y	Self	Snowball	N	Y	N	Y	N	N								
Ellis et al. (2019)	Y	Y	Self	Snowball	N	Y	N	Y	N	N								
Eyal et al. (2006)	N	N	Self	Random	N	Y	N	Y	N	N								
Fair and Patel (2019)	N	N	Self	Random	N	Y	N	Y	N	N								
Fair and Savla (2019)	N	N	Self	Random	N	Y	N	Y	N	N								
Frissen (2019)	Y	N	Self	Stratified	N	Y	N	Y	N	N								
Frissen (2020)	Y	N	Self	Stratified	N	Y	N	Y	N	N								
Frissen et al. (2019)	Y	N	Self	Stratified	N	Y	N	Y	N	N								
Gentzkow and Shapiro (2004)	N	N	Self	Representative	N	Y	N	Y	N	N								
Goede et al. (2019)	Y	N	Self	Random	N	Y	N	Y	N	N								
Gvirsman et al. (2016)	N	N	Self	Mixed	Y	Y	N	Y	N	N								
Hawthorne (2016)	N	N	Self	Convenience	N	Y	N	Y	N	N								
Holt and Kilger (2012)	N	N	Self	Random	N	Y	N	Y	N	N								
Holt et al. (2017)	N	N	Self	Stratified	N	Y	N	Y	N	N								
Jones and Paris (2018)	N	N	Self	Random	N	Y	N	Y	N	N		Y	N	Y	Y	NR	N	N
Kalmoe (2014)																		
Study 1	N	N	Self	Random	N	Y	N	Y	N	N		Y	NR	Y	Y	NR	N	N
Study 2	N	N	Self	Random	N	Y	N	Y	N	N		Y	NR	Y	Y	NR	N	N
Study 3	N	N	Self	Convenience	N	Y	N	Y	N	N		Y	NR	Y	Y	NR	N	N
Kaltenthaler et al. (2018)	N	N	Self	Representative	N	Y	N	Y	N	N								
Kremerman et al. (2012)	Y	N	Self	Convenience	N	Y	N	Y	N	N								
LaFree and Morris (2012)	N	N	Self	Probability	N	Y	N	Y	N	N								
Lee (2018)	N	N	Self	Random	N	Y	N	Y	N	N								
Luchsinger (2018)	N	N	Self	Purposive	N	Y	N	Y	Y	N								
Luchsinger (2020)	N	N	Self	Purposive	N	Y	N	Y	Y	N								
Manzoni et al. (2019)	Y	N	Self	Stratified	N	Y	N	Y	N	N								
Mock (2014)	N	N	Self	Representative	N	Y	N	Y	N	N								
Mourão et al. (2016)	N	N	Self	Probability	N	Y	N	Y	N	N								
Mulyana (2020)	N	N	Self	Random	N	Y	N	N	Y	Y								
Nivette et al. (2017)	N	N	Self	Stratified	N	Y	N	Y	Y	Y								
Pauwels and Schils (2016)	N	N	Self	Random	N	Y	N	Y	Y	Y								
Pauwels and Hardyns (2018)	N	N	Self	Random	N	Y	N	Y	Y	Y								
Pauwels et al. (2020)	N	N	Self	Random	N	Y	N	Y	Y	Y								
Pedersen et al. (2018)	N	N	Self	Representative	N	Y	N	Y	Y	Y								
Piazza and Guler (2019)	N	N	Self	Representative	N	Y	N	Y	Y	Y								
Sahani (2018)	Y	N	Open	Convenience	N	Y	N	Y	Y	Y								
Schbley (2004)	N	N	Self	Convenience	N	Y	N	Y	N	N								
Schröder et al. (2020)	Y	N	Self	Random	N	Y	N	Y	Y	Y								
Schumann et al. (2021)	N	N	Self	Purposive	N	Y	N	Y	Y	Y								
Shortland et al. (2020)	Y	N	Self	Random	N	Y	N	Y	Y	Y		Y	NR	Y	N	N	N	N
Sirgy et al. (2019)	N	N	Self	Stratified	N	Y	N	Y	Y	Y								
Storm et al. (2020)	N	N	Self	Representative	N	Y	N	Y	Y	Y								
Tang et al. (2020)	N	N	Self	Purposive	N	Y	N	Y	Y	Y								
Wojcieszak (2010)	N	N	Self	Purposive	N	Y	N	Y	N	N								
Wolfowicz et al. (FC)	N	N	Self	Random	N	Y	N	Y	N	N								
Wong et al. (2019)	Y	N	Self	Random	N	Y	N	Y	N	N								
Zhu et al. (2020)	N	Y	Self	Random	Y	Y	N	Y	N	N								

Abbreviations: N, no; NR, not reported/insufficient information; Self, self‐reported; Y, yes.

#### 5.2.1 Outcomes

For the experimental studies, one study used a validated measure of radicalization (Shortland et al., 2020), whereas the other studies used a variety of common, yet unvalidated measures assessing justification of a variety of radical behaviors.

For observational studies, the most common measures of cognitive radicalization were single item measures of justification of suicide bombings/terrorism. While this is a widely used and accepted measure of cognitive radicalization (see Schmid, 2017 for a discussion on this), it is not a validated measure. A few other studies used adapted versions of validated instruments to measure analogous outcomes, however these adapted instruments have either not been validated or otherwise lack evidence that they are valid measures for the outcomes of interest (e.g., Eyal et al., 2006; Gvirsman et al., 2016; Kremerman et al., 2012).

Only three groups of studies used fully validated measures, including the Sympathies for Radicalization scales (SyFor; Bhui et al., 2016; Frissen, 2019, 2020) the Activism‐Radicalism‐Intentions Scales (ARIS; Ellis et al., 2016, 2019; Frissen, 2019, 2020), and studies comparing violent with non‐violent radicals (Clemmow et al., 2020; Sahani, 2018).

#### 5.2.2 Independent variables

Among the experimental studies, treatment included manipulated exposure to a piece of mediated content that was theorized to increase the risk of radicalization compared to a neutral piece of content, in line with standard practices in media‐effects research (Ferguson & Savage, 2012). For two studies (Azeem et al., 2019; Kalmoe, 2014), treatment included exposure to manipulated news items. Another study included comparisons of exposure to highlight reels from different action films against a control group, as well exposure to video concerning a political protest against a control group (Jones & Paris, 2018). Only one study included a treatment of exposure to explicitly radical content in the form of an ISIS propaganda video (Shortland et al., 2020).

None of the included studies relied upon a validated measure of media‐usage/exposure. Rather, they drew on self‐reported measures of media usage and engagement with certain types of activities or content. Self‐reports such as these have the potential for both over and underestimation of actual usage. Nevertheless, they are the most common way in which the effects of different types of media exposure are examined in observational studies examining related outcomes (even if only distantly) such as aggression and general delinquency (Ferguson & Savage, 2012). When self‐reports are used to measure exposure to specific types of content, there is an added source of potential bias, as specific interest in the topic of the content (e.g., politics) may influence memory recall of exposure to it (Donohew et al., 1998; Slater, 2004). This added source of potential bias is relevant to those risk factors included in the review which measure exposure to specific types of content (e.g., political, radical, or violent).

#### 5.2.3 Report type

The studies were primarily based on self‐reported measures of both the dependent and independent variables. One of the studies measuring behavioral radicalization was based on a database derived from open sources that included a group of violent radicals and a control group of non‐violent radicals (Sahani, 2018). Another study measuring behavioral radicalization was also based on an open‐source database of convicted terrorists and compared it with a general population survey relying on self‐reported measures (Clemmow et al., 2020).

While the broader radicalization literature includes some examples of clinical assessments of radicalization, these are usually made as part of risk assessments for individuals that have been referred to various authorities (e.g., Pfundmair et al., 2022). Self‐reports of cognitive radicalization remain the dominant report type in the literature and are even common for measuring sub‐terroristic forms of radical behaviors (see Wolfowicz, Weisburd, et al., 2021a). The studies included in this review are therefore representative of the types of studies found in the broader literature on radicalization.

#### 5.2.4 Sampling strategy

Of the included studies, the most common strategy was to employ random sampling (*N = *17), followed by representative sampling (*N =* 9) and stratified sampling (*N = *7). Purposive sampling was used in *N = *4 studies, Convenience sampling in *N* = 5 studies, snowball sampling in *N* = 2 studies, probability sampling in *N *= 2 studies, non‐probability in *N = *1 study, and mixed sampling strategy in another *N *= 1 study.

#### 5.2.5 Temporal ordering

In experimental studies, it was not evident that Time 1 measures of the outcome were controlled for, nor did any of the studies report on the collection of such measures.

Regarding observational studies, the review included only two longitudinal studies. These studies did not report on the same set of factors and as such it was not possible to conduct a separate meta‐analysis for longitudinal studies (Gvirsman et al., 2016; Zhu et al., 2020).

For two studies that examined radical behaviors (Clemmow et al., 2020; Sahani, 2018), exposure to the risk factors inherently preceded the outcome of radical offending behaviors.

It is not possible to determine that exposure to the independent variable preceded the onset of the dependent variable for the remainder of the studies (*N *= 45) as they are cross‐sectional. Additionally, in the two experimental studies, pre‐test measures of the outcome were not taken.

The issue of temporal ordering precludes the ability to draw causal inferences from the results, and all interpretations should be weighed against this limitation.

#### 5.2.6 Statistical controls

As noted earlier, we use a risk‐factor approach in which the main outcome measures are based on uncontrolled correlations. Statistical controls in the multiple regression models were appropriate, most commonly accounting for basic socio‐demographic variables such as age and gender. Most studies provided correlation matrices that included other variables, however, the combination of these variables differed considerably across studies. As such, it was not feasible to conduct a separate analysis on partial effect sizes controlling for similar sets of confounding variables.

#### 5.2.7 Missing data

Few of the studies noted any issues with missing data. However, this is not necessarily an indication that missing data did not exist as it is very common for publications to fail to note this issue. A few studies that noted the presence of missing data noted that it was handled either through exclusion from the analysis or using estimation techniques.

#### 5.2.8 Non‐significant results

There was no clear indication of non‐significant result reporting bias in all but one of the studies (Calenda & Mosca, 2007), which stated that certain relationships existed but failed to report on them.

#### 5.2.9 Excluded variables

There were *N =* 3 studies that explicitly mentioned variables that were apparently available in the data but then absent in reporting of the results. There were also several studies that relied on secondary data which are known to contain other potentially relevant variables that were not included (*N *= 6). There were *N *= 5 studies that had the ability to report on different types of media‐related factors separately but only reported on them as an aggregate measure. For those studies for which such bias was not evident, we are still unable to fully discount the possibility that variables were omitted as studies were not pre‐registered.^4^

#### 5.2.10 Excluded outcomes

Two studies (Frissen, 2019, 2020) relied on a combined measure of cognitive radicalization, made up of both the SyFor and Activism‐Radicalism‐Intentions‐Scale (ARIS). While other radicalization studies not included in this review have taken a similar approach (e.g., Rousseau et al., 2019), these scales may represent distinct constructs (Wolfowicz et al., 2020) and it thus would have been informative to have had the ability to assess effect sizes as they pertain to each of the scales separately.

In one study (Calenda & Mosca, 2007) correlations between different versions of the dependent variable and independent variables are reported on. In some cases, this study reported on individual items that made up the dependent variable, whereas in other cases they report on correlations with the index dependent variable.

In another study (Mulyana, 2020), insufficient information is given about the dependent variable. However, as it is described, it appears that an additional measure of radical intentions is available but not reported on.

In Pauwels and Schils (2016) study (see also Pauwels & Hardyns, 2018; Pauwels et al., 2020), only one version of the dependent variable is reported. However, in data supplied by the authors multiple versions of the dependent variable were available.

There was no evidence of pre‐published or registered protocols for any of the studies. As such, even where there was no evidence of excluded outcome bias, we are still unable to discount the possibility that they employed more than one outcome measure but only reported on one. We are also unable to discount the possibility that additional models were used in which the dependent variable was measured differently (e.g., dichotomized).^5^

#### 5.2.11 Additional items for experimental studies

##### 5.2.11.1 Allocation sequence

For three of the experimental studies, allocation sequence was reported as having been made at random. Allocation sequence was not explicitly mentioned in *n *= 1 study (Azeem et al., 2019).

##### 5.2.11.2 Baseline differences

Experimental studies differed significantly with respect to the reporting of baseline differences between groups. Azeem et al. (2019) reported some basic characteristics and checked for differences using ANOVA. Another study included extensive baseline characteristics in the supplemental materials (Jones & Paris, 2018). Two other studies make no mention of assessing baseline characteristics, nor were such characteristics detailed. However, these studies did include age and gender as controls in their statistical models (Kalmoe, 2014; Shortland et al., 2020).

##### 5.2.11.3 Appropriate analysis

Two of the experimental studies used OLS regression to analyze the data and report results (Kalmoe, 2014; Shortland et al., 2020), and another used a one‐way ANOVA (Azeem et al., 2019). Another study compared groups using *t* tests (Jones & Paris, 2018). For Kalmoe (2014), as well as Jones and Paris (2018), supplementary materials were also available that provided additional information.

##### 5.2.11.4 Failure to analyze participants

For experimental studies there was no indication of a potential for a substantial impact (on the result) due to a failure to analyze participants in the group to which they were randomized.

##### 5.2.11.5 Pre‐specified analysis plan

For experimental studies, there is no evidence of a pre‐specified analysis plan having been in place before data were available for analysis.

##### 5.2.11.6 Multiple outcomes

For experimental studies there was no evidence that results were selected on the basis of the results from multiple outcome measurements.

##### 5.2.11.7 Multiple analyses

For experimental studies, given the choice of analysis (OLS) over a simpler comparison of means (such as *t* tests), we cannot discount the possibility that results were selected based on results stemming from multiple analyses. However, it is of note that the studies also reported on results that were not statistically significant.

In the case of the Shortland et al. (2020) study, the OLS regression models may be open to the influence over misspecification by analyzing multiple interaction effects, and multiple overlapping comparisons in a single model.

### 5.3 Synthesis of results

#### 5.3.1 Observational studies

A total of 23 factors were identified for the outcome of cognitive radicalization, consisting of 3 factors with negative associations and 20 factors with positive associations with the outcome. Only two factors were identified for the outcome of behavioral radicalization (see Table 6 for a description of these factors). Following the classification of very small, small, moderate, and large estimates, most of the factors had very small‐small estimates (Hopkins, 2002).

Among the factors with negative associations were Internet Access, Newspaper reading, and Radio listening. In addition to the effect sizes being exceptionally small, they were also not statistically significant. Among factors with positive associations, 12 had very small estimates ranging from *r = *0.01 to 0.08. These included, in rank order: Television consumption, Facebook usage, using the Internet to consume political news, engaging in content piracy, time spent online, trust in media, Twitter usage, posting of political content online, general media consumption, online network participation, technical skills, and being a victim of cyberbullying. The next tier included factors with small to moderate estimates (*r = *0.10 to 0.26). Following the rank order, similar estimates were found for factors pertaining to general components of Internet and media usage. This included: Self‐censorship online, hacking, usage of online discussion forums, attachment to online networks, active seeking of radical content online, passive consumption of radical content online, and consumption of ISIS news. An estimate that can be categorized as large was found for a single factor, namely willingness to engage in ideologically motivated cyber‐attacks (*r* = 0.57).

The results show that certain attitudinal and experiential factors, as they pertain to the Internet, have effect sizes that are not substantively different from general Internet usage. They also show that more specific forms of online activity, as well as some attitudinal factors, in particular a willingness to engage in ideologically motivated cyber‐attacks, demonstrate more robust relationships with radicalization than general forms of consumption.

As opposed to cognitive radicalization, for behavioral radicalization the included studies only provided effect sizes pertaining to two factors for which meta‐analysis was possible, namely passive and active forms of consumption and engagement with radical content. The pooled estimate for active engagement with radical Internet‐based content (*r* = 0.28 [95% CI = 0.21, 0.36], *k* = 5, *I* ^2^ = 78.49) was slightly larger than the estimate for passive exposure to such content (*r* = 0.23 [95% CI = 0.12, 0.33], *k *= 4, *I* ^2^ = 91.10; see Table 8).

**Table 8:** Meta‐analysis.

	Factor	*r*	LCI‐UCI	*Q*	*I* ^2^	*τ* ^2^	*N* (k)
	*Cognitive radicalization*						
	Negative effects						
*Very small*	Internet access	−0.02†	−0.03, 0.00	23.50*	53.19	0.000	67,693 (12)
	Radio	−0.07	−0.17, 0.03	113.03***	96.46	0.012	15,402 (5)
	Newspaper	−0.08†	−0.17, 0.01	77.61***	94.85	0.009	15,047 (5)
	Positive effects						
*Very small*	Television	0.01	−0.06, 0.09	435.013***	97.01	0.019	27,707 (14)
	Facebook	0.02	−0.07, 0.11	139.66***	96.42	0.011	44,200 (6)
	Internet politics	0.02*	0.00, 0.04	7.332	45.41	0.000	48,348 (5)
	Piracy	0.04	−0.07, 0.14	187.790***	97.34	0.016	16,761 (6)
	Time online	0.05***	0.03, 0.06	4.805	0.00	0.000	16,987 (7)
	Trust media	0.05†	−0.01, 0.11	.564	0.00	0.000	1116 (3)
	Twitter	0.06	−0.03, 0.15	120.759***	95.86	0.010	43,458 (6)
	Posting political	0.09***	0.06, 0.12	16.602**	69.88	0.001	20,094 (6)
	General media	0.08***	0.04, 0.12	153.430	93.48	0.004	47,386 (11)
	Participation	0.08***	0.06, 0.100	0.261	0.00	0.000	10,958 (2)
	Tech skills	0.08**	0.03, 0.14	0.446	0.00	0.000	1132 (2)
	Cyberbullying	0.08***	0.07, 0.10	6.079	34.20	0.000	30,120 (5)
*Small*	Self‐censoring	0.12***	0.06, 0.18	.093	0.00	0.000	1108 (2)
	Hacking	0.13	−0.06, 0.33	12.554***	92.04	0.018	1318 (2)
	Forums	0.17***	0.080, 0.26	.755	0.00	0.000	429 (2)
	Attachment	0.20***	0.09, 0.30	131.58***	96.20	0.016	14,225 (6)
	Active seeking	0.22***	0.15, 0.29	11.07**	81.93	0.003	6554 (3)
	Passive Internet	0.24***	0.18, 0.31	116.671***	93.14	0.009	21,422 (9)
	ISIS news	0.26	−0.07, 0.54	227.90***	98.68	0.119	2766 (4)
Lrg.	Cyber‐attack (intent)	0.57***	0.35, 0.78	38.36***	94.79	0.035	1671 (3)
	*Behavioral radicalization*						
*Small*	Passive	0.23***	0.12, 0.33	33.688***	91.10	0.012	6031 (4)
	Active	0.28***	0.21, 0.36	18.599**	78.49	0.006	5882 (5)

Abbreviations: 95% CI, 95% lower and upper confidence intervals; *I* ^2^, heterogeneity statistic; *k*, number of studies; *N*, total sample size; *Q*, Cochran's *Q* heterogeneity statistic and *χ* ^2^ test *p* value; *r*, correlation coefficient.

† <0.10.

*** <0.000.

** <0.01.

* <0.05.

#### 5.3.2 Experimental studies

The experimental studies included in this review were combined in a single meta‐analysis to examine the effects of 'one‐off exposure to radicalizing content'. However, the nature of the exposure differed between the studies. Kalmoe (2014) used a vignette design, exposing the treatment group to violent or neutral political messages, whereas Azeem et al. (2019) manipulated exposure to either positive or negative news stories concerning political events. Jones and Paris (2018) used exposure to clips from action‐drama films, as well as from broadcasts concerning political protests. Shortland et al. (2020) was the only study to expose the treatment group to an explicitly radical video, being an approximately 6‐min‐long propaganda video produced by the ISIS terrorist group. Two of the studies, which included a total of four samples (Kalmoe, 2014; Shortland et al., 2020), also examined interactions between exposure to the respective experimental conditions and trait aggression (Table 9).

**Table 9:** Meta‐analysis of experimental studies.

Factor	*g*	LCI‐UCI	*Q*	*I* ^2^	*τ* ^2^	*N* (*k*)
*Cognitive radicalization*						
Exposure	0.08	−0.03, 0.19	26.243**	58.09	0.021	3196 (12)
Exposure × Aggression	0.13*	0.01, 0.25	4.346	30.97	0.005	1541 (4)

Abbreviations: 95% CI, 95% lower and upper confidence intervals; *g*, Hedge's *g* (bias corrected Cohen's *d*); *I* ^2^, heterogeneity statistic; *k*, number of studies; *N*, total sample size; *Q*, Cochran's *Q* heterogeneity statistic and *χ* ^2^ test *p* value; *r*, correlation coefficient.

*** <0.000.

^+^<0.10.

** <0.01.

* <0.05.

**Figure 3:**
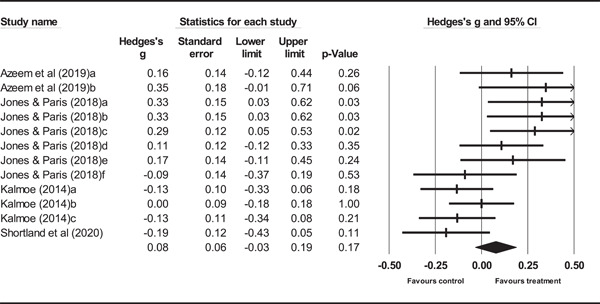
Exposure on cognitive radicalization.

The results of the random effects meta‐analysis (*k* = 12, *n *= 3196) found a pooled estimate of *g *= 0.08 [95% CI = −0.03, 0.19], which presented with more than a moderate degree of heterogeneity (*Q* = 26.643, *p* = 0.003, *I* ^2^ = 58.09).^6^ The pooled results are reflective of what was found in the primary studies, namely that simple exposure to the theorized risk‐inducing condition was associated with a small reduction in risk (Figure 3).

**Figure 4:**
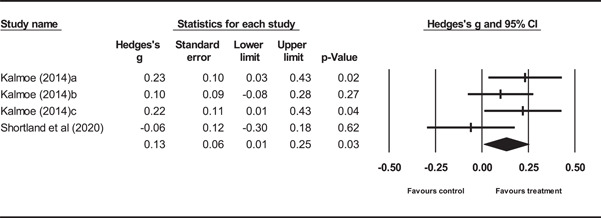
Exposure × Trait aggression interaction.

When looking at the interactions with trait aggression, exposure to the experimental conditions had a pooled estimate of *g =* 0.13 [95% CI = 0.01, 0.25]. Heterogeneity was found to be moderate (*Q *= 4.346, *p* = 0.226, *I* ^2^ *= *30.97). The results show that in the presence of high trait aggression, simple exposure to media theorized to radicalize was associated with a small increase in risk, larger than exposure on its own (Figure 4).

#### 5.3.3 Heterogeneity

As per Table 3, heterogeneity was found to be very high (*I* ^2^ > 75) for 13/23 factors derived from observational studies for cognitive radicalization, and both the factors pertaining to behavioral radicalization. Heterogeneity was high for one additional factor, moderate for two factors, and absent for seven factors, of which four factors were made up of only two effect sizes. Heterogeneity was moderate for both analyses of experimental studies. As such, and following the protocol, we sought to investigate a range of potential sources of heterogeneity.

##### 5.3.3.1 Study‐level characteristics

Univariate meta‐regression analysis was used to assess the effects of three continuous factors: year of data collection of samples, the mean age of studies' samples, and the proportion of males in samples. The analyses were carried out for factors for which information pertaining to these variables were available for at least 6 studies. The analyses for each factor were carried out separately, with separate analyses conducted for each of the study‐level characteristics as they pertain to each of the risk factors. In total, the analysis for year of data collection was carried out for six factors. However, year of data collection was found to only have a marginally significant impact on the estimate for passive exposure to radical content online. For all other factors the estimate was not statistically significant. For mean age of samples, the analysis was only possible for one factor, Twitter usage, where it was found to have a statistically significant, negative impact on the pooled estimate. A marginally significant effect was found for experimental exposure as well. For proportion of males in a sample, the analysis was carried out on six factors. The only statistically significant effect was found for experimental exposure (Table 10).

**Table 10:** Meta‐regressions for study‐level characteristics.

Factor	*k*	*B* (SE)	95% CI	*p*
*Cognitive*				
Internet access				
Year of data collection	12	−0.003 (0.012)	−0.03, 0.02	0.821
% Males in sample	11	−0.000 (0.003)	−0.01, 0.01	0.859
Facebook				
Year of data collection	6	0.011 (0.027)	−0.04, 0.06	0.700
% Males in sample	6	−0.000 (0.003)	−0.01, 0.01	0.859
Piracy				
Year of data collection	6	0.010 (0.018)	−0.03, 0.04	0.587
% Males in sample	6	0.002 (0.014)	−0.02, 0.03	0.881
Time online				
Year of data collection	6	−0.002 (0.002)	−0.00, 0.00	0.363
Twitter				
% Males in sample	6	0.000 (0.004)	−0.01, 0.01	0.920
Mean age of sample	6	−0.005 (0.002)	−0.01, 0.00	0.014
Passive exposure				
Year of data collection	8	0.037 (0.021)	−0.00, 0.08	0.080
% Males in sample	7	0.008 (0.005)	−0.00, 0.02	0.100
Network attachment				
Year of data collection	6	0.034 (0.035)	−0.04, 0.10	0.331
% Males in sample	6	−0.000 (0.002)	−0.01, 0.00	0.856
General media				
Year of data collection	11	0.004 (0.005)	−0.01, 0.01	0.437
% Males in sample	6	0.000 (0.003)	−0.01, 0.01	0.998
*Experimental*				
Year of data collection	12	0.015 (0.016)	−0.0, 0.06	0.345
Age	11	−0.008 (0.004)	−0.02, −0.00	0.030
% Males in sample	12	−0.007 (0.004)	−0.02, 0.00	0.084

##### 5.3.3.2 Region

There were an insufficient number of studies to enable examining heterogeneity between countries. As such, studies were pooled together by region or country type to facilitate a meaningful analysis. The analysis was carried out for ten of the factors pertaining to cognitive radicalization, and one factor pertaining to behavioral radicalization (see Table 11).

**Table 11:** Moderator analysis for region.

**Factor**	**Region**	***k***	***r(g)***	**95% CI**	***Q***_**Between**_	***p***
*Cognitive*						
ISIS News	EU	2	0.38*	0.05, 0.64	0.904	0.342
	Canada	3	0.13	−0.28, 0.51		
Radio	EU	2	−0.02	−0.19. 0.16	0.328	0.567
	Other (mixed)	3	−0.10	−0.31, 0.12		
Facebook	EU	2	0.03	−0.26, 0.31	0.048	0.827
	Other (mixed)	4	−0.00	−0.07, 0.06		
Posting political	EU	4	0.09***	0.05, 0.14	0.014	0.907
	Other (mixed)	2	0.09***	0.07, 0.11		
Piracy	EU	4	0.06	−0.07, 0.19	1.379	0.240
	Other (mixed)	2	−0.03	−0.10, 0.05		
Time online	Western	3	0.08*	0.00, 0.17	0.816	0.366
	Non‐Western	4	0.05***	0.03, 0.06		
Twitter	EU	2	0.13***	0.06, 0.21	5.068	0.079
	US	2	0.04	−0.08, 0.14		
	Other (mixed)	2	0.02	−0.07, 0.10		
Passive exposure	EU	6	0.25***	0.17, 0.33	0.043	0.836
	Other (mixed)	3	0.23**	0.10, 0.35		
Network attachment	Western	3	0.14	−0.17, 0.41	0.433	0.510
	Non‐Western	3	0.27†	−0.03, 0.53		
General media	EU	6	0.12***	0.07, 0.16	6.788	0.009
	Other (mixed)	5	0.03	−0.03, 0.08		
*Behavioral*						
Active exposure	EU	2	0.29***	0.26, 0.31	5.537	0.019
	US	2	0.21***	0.15, 0.27		

For cognitive radicalization, statistically significant differences were found for only one factor, and marginally significant differences were found for two additional factors. For General media use, the estimate for studies from EU countries (*k *= 6) of *r =* 0.12 was significantly larger (*Q*_between_ = 6.788, *p* = 0.009) than the estimate for studies from other countries (*k* = 5) of .03. For Posting of political opinions online, there was marginally significant between‐group heterogeneity (*Q*_between_ = 2.743, *p* = 0.098) for EU studies (*k* = 3), which had a pooled estimate of *r =* 0.06 and other countries (*k* = 2), which had a pooled estimate of *r =* 0.09. For Twitter usage, the estimate for EU‐based studies (*k* = 2) of *r = *0.13, was larger (*Q*_between_ = 5.068, *p* = 0.079) than the estimate for studies from the US (*k* = 2) of *r = *0.04 and from other countries (*k* = 2) of *r = *0.02.

##### 5.3.3.3 Ideological strain

Due to the small number of studies identified in the review, it was only possible to analyze heterogeneity based on the ideological strain examined for *N *= 7 risk factors, among which there were no cases of statistically significant differences between‐ideology heterogeneity. For experimental exposure, while studies examining Islamist radicalization had a noticeably larger estimate, the between‐group heterogeneity was not statistically significant (Table 12).

**Table 12:** Moderator analysis for ideological strain.

**Factor**	**Ideology**	***k***	***r***	**95% CI**	***Q***_**Between**_	***p***
*Cognitive*						
Cyberbullying	Right‐wing	2	0.07***	0.05, 0.09	0.582	0.446
	Islamist	2	0.08***	0.06, 0.11		
Radio	Islamist	2	−0.16	−0.44, 0.15	0.810	0.368
	Other (mixed)	3	−0.04	−0.19, 0.12		
Newspaper	Islamist	3	−0.10†	−0.21, 0.02	0.601	0.348
	Other (mixed)	2	−0.05	−0.12, 0.03		
Posting political	Islamist	2	0.08***	0.06, 0.11	0.439	0.508
	Other (mixed)	3	0.10***	0.05, 0.15		
Time online	Islamist	5	0.05***	0.03, 0.07	0.233	0.629
	Other (mixed)	2	0.04***	0.02, 0.07		
Passive exposure	Islamist	3	0.24**	0.11, 0.37	4.127	0.127
	Left‐wing	2	0.35***	0.20, 0.47		
	Other (mixed)	3	0.16**	0.04, 0.27		
General media	Right‐wing	3	0.12**	0.03, 0.21	1.249	0.536
	Islamist	3	0.05	−0.03, 0.14		
	Other (mixed)	5	0.07*	0.01, 0.14		
*Experimental*						
Exposure	Islamist	2	0.23*	0.01, 0.45	1.954	0.162
	Other (mixed)	10	0.05	−0.07, 0.17		

##### 5.3.3.4 Other analyses

###### 5.3.3.4.1 Effect size derivation

For experimental studies, there were from the standardization of regression coefficients and eight effect sizes that were calculated from reported means and standard deviations (or standard errors). For the former group of studies (*k *= 4) the pooled estimate was a negative *g = *−0.10 (95% CI [−0.20, 0.00]), whereas for the latter group (*k* = 8) the pooled estimate was *g = *0.19 (95% CI [0.09, 0.29]). The between‐group heterogeneity was statistically significant (*Q*_between_ = 16.341, *p* = 0.000), indicating that the combination of effect sizes derived from different sources is a significant source of heterogeneity. In conducting a new analysis that excluded studies which only provide results from regression models, heterogeneity was found to be very low (*Q* = 7.425, *p* = 0.386, *I* ^2^ *=* 5.72).

For observational studies, with respect to the factor General media use, the pooled estimate for effect sizes derived from bivariate sources (*k *= 8) was *r = *0.11 (95% CI [0.07, 0.15]), and for standardized partial effect sizes (*k* = 3) the pooled estimate was *r = *−0.00 (95% CI [−0.05, 0.04]). The between‐group heterogeneity was statistically significant (*Q*_between_ = 13.806, *p* = 0.000).

###### 5.3.3.4.2 Type of content

For general media use, studies examining exposure to news media (*k* = 4) had a pooled estimate of 0.04 (95% CI [−0.00, 0.08]) and studies examining exposure to violent media (*k *= 7) had a pooled estimate of 0.10 (95% CI [0.05, 0.15]). The between‐group heterogeneity was found to be marginally significant (*Q*_between_ = 3.062, *p* = 0.080).

For passive exposure to online content, the pooled estimate for studies measuring exposure to radical content (*N* = 7) was *r =* 0.28 (95% CI [0.21, 0.34]). and for studies measuring exposure to violent content (*N* = 2) it was *r =* 0.10 (95% CI [0.04, 0.16]). Between‐group heterogeneity was found to be statistically significant (*Q*_between_ = 8.607, *p* = 0.003), suggesting that the estimate for exposure to radical content is significantly larger than the estimate for exposure to violent content. Similarly, for Television usage, an additional analysis was carried out based on studies that measured exposure to violent television and following the news or politics through television. The pooled estimate for violent television was 0.03 (95% CI [−0.09, 0.15]) and for news/politics television was 0.01 (95% CI [−0.09, 0.11]. This small difference was not statistically significant (*Q*_between_ = 0.053, *p* = 0.818).

For active exposure to online radical content, three studies measured actively seeking out radical content or connections online. Two of these studies (by the same group of authors) provided information for different types of content seeking, covering general information, radical videos, beheading videos, and *Dabiq* and *Inspire*, the publications of ISIS/ISIL and Al‐Qaeda respectively. The results of the analyses are provided below in Table 8. The findings show that seeking information about radical groups has the largest effect size among these factors, followed by seeking out of both ISIS and Al‐Qaeda publications, seeking out of radical videos, and seeking out beheading videos. The effect sizes range from small (beheading, *r* = 0.16, 95% CI [0.11, 0.21]) to moderate (info‐seeking, *r* = 0.31, 95% CI [0.27, 0.36]). Table 13 presents these results.

**Table 13:** Sub‐group analysis for different types of active seeking (online).

Factor	*r*	95% CI	*Q*	*I* ^*2*^	*N* (*k*)
Dabiq	0.27**	0.07, 0.47	11.519**	91.319	2189 (2)
Inspire	0.29**	0.07, 0.51	13.489***	92.587	2189 (2)
Info‐seeking	0.31***	0.27, 0.36	0.699	0.000	2189 (2)
Video‐seeking	0.19**	0.08, 0.30	3.743†	73.285	2189 (2)
Beheading vid	0.16***	0.11, 0.21	1.101	9.186	2189 (2)

Abbreviations: 95% CI, 95% lower and upper confidence intervals; *I* ^2^, heterogeneity statistic; *k*, number of studies; *N*, total sample size; *Q*, Cochran's *Q* heterogeneity statistic and *χ* ^2^ test *p* value; *r*, correlation coefficient.

*<0.05.

† <0.10.

*** <0.000.

** <0.01.

For the outcome of behavioral radicalization and the factor of passive exposure, two effect sizes pertained to exposure to radical content and two to non‐specific measures of social media usage. Moderator analysis was carried out and the pooled estimate for the former was 0.17 (95% CI [0.08, 0.26]) and for the latter was 0.29 (95% CI [−0.01, 0.54]). However, between‐group heterogeneity was not statistically significant (*Q*_between_ = 0.559, *p* = 0.455), indicating that the estimates did not significantly differ from each other. A second moderator analysis was carried out for studies comparing terrorists with non‐terrorists, and those examining self‐reported radical violence. The pooled estimate for the former group was 0.13 (95% CI [0.07, 0.18]) and for the latter was 0.32 (95% CI [0.10, 0.51]). While the differences in the size of the estimates is apparent, the difference was only marginally significant as indicated by the extent of between‐group heterogeneity (*Q*_between_ = 2.755, *p* = 0.097).

##### 5.3.3.5 Leave‐one‐out analysis

Leave‐one‐out analyses were carried out for all analyses that included >3 effect sizes and for which the *I* ^2^ statistic indicated any heterogeneity (>0). The analyses are presented for all factors for which the removal of a single study led to a significant reduction of heterogeneity. Significant reductions in heterogeneity were found for 9/14 factors analyzed. Heterogeneity was reduced from very high to high for two factors. Heterogeneity was reduced to 0 for four of the factors, although for one of these factors the removal of the one study left only two effect sizes in the analysis. For one factor, ISIS news, although heterogeneity was not significantly reduced, there was a meaningful impact on the pooled estimate, increasing from a non‐significant *r = *0.26 (95% CI [−0.07, 0.54]) to a statistically significant *r =* 0.37 (95% CI [0.17, 0.54]). Another meaningful change in pooled estimates was found for Network attachment, with a reduction from *r = *0.20 to *r =* 0.07 (95% CI [0.03, 0.11]). These results indicate that the pooled estimates for at least some of the factors are sensitive to outlier effects. This means that caution is warranted in accepting the rank order of the summary results in Table 3 as representing the true rank order of the relative magnitude of effects. At the same time, most of the factors with small, and essentially inconsequential effects, display little or no difference in this analysis, indicating that they are more robust to the impacts of outlier bias (Table 14).

**Table 14:** Leave‐one‐out analysis.

Factor	*r/g*	^Adjusted^ *r*/g	LCI‐UCI	*Q*	*I* ^2^	*N* (k)
*Cognitive*						
Access	−0.02	−0.02*	‐0.03, 0.00	16.07†	37.77	67,142 (11)
Active	0.22	0.25***	0.21, 0.29	0.053	0.00	2189 (2)
Attachment	0.20	0.07**	0.03, 0.11	12.83*	68.81	13,935 (5)
Cyberbullying	0.08	0.08***	0.07, 0.09	0.925	0.00	24,423 (4)
Cyber‐attacks	0.57	0.58***	0.49, 0.66	3.469†	71.17	892 (2)
Facebook	0.02	−0.02	−0.09, 0.04	13.85**	71.11	38,723 (5)
Internet politics	0.02	0.01**	0.00, 0.02	2.31	0.00	41,761 (4)
ISIS news	0.26	0.37**	0.17, 0.54	36.61***	94.54	1664 (3)
Piracy	0.04	−0.010	−0.04, 0.03	8.48†	52.83	11,064 (5)
Twitter	0.06	0.01**	0.00, 0.02	0.797	0.000	37,981 (4)
*Behaviors*						
Active	0.23	0.25***	0.19, 0.30	6.41†	53.19	5666 (4)
Passive	0.28	0.17***	0.10, 0.23	7.12*	71.89	5577 (3)
*Experimental*						
*Exposure *× *Aggression*	0.13*	0.18**	0.07, 0.29	1.202	0.000	1263 (3)

*Note*: The estimates for factors pertaining to factors under the categories of “cognitive” and “behaviors” are reported as *r* correlations and for “experimental” as Hedge's *g*.

#### 5.3.4 Single effect sizes

The systematic review identified several other media‐related factors for which only a single effect size was found, and thus meta‐analysis was not possible. However, it is important to consider the nature of these factors as part of the broader scope of the review.

##### 5.3.4.1 Other mediums

Manzoni et al.'s (2019) measure of radical content exposure included an item relating to listening to extremist music and attending concerts. The descriptive statistics show that the means for the music related variables were larger than for other items that make up the variable, which had small to moderate correlations with both right‐wing radical attitudes (*r* = 0.30, 95% CI [0.27, 0.33]) and left‐wing radical attitude (*r* = 0.24, 95% CI [0.22, 0.26]).

##### 5.3.4.2 Norms and rules

Holt et al. (2017) examined several variables that measured perceptions concerning online norms and rules and provided correlations with radical attitudes. The study found statistically significant negative correlations for the following variables: (1) Rules, which measured the extent to which respondents felt that there “are clear rules on what is acceptable, ethical behavior online” (*r =* −0.06, 95% CI [−0.13, 0.01]; *r = *0.004, 95% CI [−0.07, 0.07]), (2) Use, which measured the extent to which respondents felt that “People should be allowed to use computers they do not own in any way they see fit” (*r =* −0.14, 95% CI [−0.21, 0.07]; *r =* −0.07, 95% CI [−0.14, 0.00]), and (3) Perceptions that law enforcement was able to recognize cybercrimes quickly (*r =* −0.21, 95% CI [−0.27, −0.14]; *r =* −0.12, 95% CI [−0.18, −0.05]). The study also found statistically significant positive correlations between measures of radical attitudes and respondents' beliefs that cybercrimes (unauthorized access to computer systems) were less serious than other crimes (*r = *0.11, 95% CI [0.04, 0.17]; *r = *0.06, 95% CI [−0.01, 0.13]). Finally, the study also examined the extent to which respondents felt that illegally accessing computer systems was beneficial for society, which was also associated with a very small and non‐significant correlation (*r = *0.02, 95% CI [−0.05, 0.09]*; r = *0.03, 95% CI [−0.04, 0.10]).

##### 5.3.4.3 Efficacy of online activities

Brunsting and Postmes (2002) examined several variables that measured perceptions of the efficacy of soft and hard forms of online activities, which pertained to online activism and hacking respectively. Perceptions of overall efficacy of online activism had a significantly smaller estimate (*r = *0.09, 95% CI [0.01, 0.17]) than perceptions of the efficacy of hacking (*r = *0.37, 95% CI [0.29, 0.44]). Estimates for perceptions of self‐efficacy of online activism (*r = *0.11, 95% CI [0.03, 0.19]) and hacking (*r = *0.15, 95% CI [0.07, 0.23]) were much more similar, however. Another factor that was examined was to what degree participants expected others to engage in these actions. In this case, the estimate for hacking (*r = *0.25, 95% CI [0.17, 0.33]) was greater than for online activism (*r = *.06, 95% CI [−0.02, 0.14]).

##### 5.3.4.4 Parental awareness of Internet activities

Goede et al. (2019) found that parental awareness of Internet activities had an overall negative correlation with radical attitudes for right‐wing radicalization (*r =* −0.07, 95% CI [−0.09, −0.05]) but a small, positive correlation with Islamist radicalization (*r* = 0.03, 95% CI [0.01, 0.05]). It is of note that increased parental involvement/control has been found to be a protective factor against radicalization, albeit with somewhat larger effect sizes (Wolfowicz, Weisburd, et al., 2021a).

##### 5.3.4.5 Other online platforms

While the review only found enough studies to meta‐analyze Facebook and Twitter usage, there are other online platforms that individuals may consume content through. Frissen et al. (2019) examined YouTube usage, although the correlation with measures of radical attitudes and intentions was not statistically significant. Goede et al. (2019) found a statistically significant but very small positive correlation (*r* = 0.04, 95% CI [0.02, 0.06]) between heavy use of online messengers and right‐wing radical attitudes. However, there was no significant correlation with Islamist radical attitudes (*r =* 0.00, 95% CI [−0.002, 0.02]).

##### 5.3.4.6 Online behaviors

Goede et al. (2019) found that giving more “likes” on social media had statistically significant positive correlations with both right‐wing (*r = *0.10, 95% CI [0.08, 0.12]) and Islamist (*r =* 0.09, 95% CI [0.07, 0.11]) cognitive radicalization. It is of note that in a case‐control study by Wolfowicz, Litmanovitz, et al. (2021) comparing terrorists with non‐violent radicals, there were no differences in the number of Facebook likes received by members of the two groups (*r = *0.01, 95% CI [−0.15, 0.17]).

##### 5.3.4.7 Differential associations

Goede et al. (2019) found that using the Internet to find friends had statistically significant positive correlations with both right‐wing (*r* = 0.14, 95% CI [0.12, 0.16]) and Islamist cognitive radicalization (*r* = 0.05, 95% CI [0.03, 0.07]).

##### 5.3.4.8 Network characteristics

In a longitudinal study, Zhu et al. (2020) found highly significant effects for the role of network heterogeneity as a mediator of social learning variables and a predictor of self‐reported radical behaviors (*r* = 0.30, 95% CI [0.17, 0.42]).

#### 5.3.5 Publication bias

Publication bias analysis was carried out for all factors that included a minimum of three effect sizes. Egger's regression was found to be marginally significant for only one factor, Network attachment, and was not statistically significant for all other factors. The Trim‐and‐Fill method identified missing effect sizes for nine factors for cognitive radicalization, one of which was Network attachment, and both factors pertaining to behavioral radicalization. In all such cases, between 1 and 2 missing studies were identified, and the maximum difference between the original estimate and the adjusted estimate did not exceed *r =* 0.09 (network attachment). Thus, most of the factors analyzed were robust against publication bias and the differences emerging from the trim‐and‐fill analysis do not change the interpretation of the results (see Table 15).

**Table 15:** Publication bias.

Factor	*r*	*k*	T&F	*r*_adjusted_	95% CI	*Q*	Egger's test *β*1 (*p* value)
*Cognitive*							
ISIS News	−0.00	4	‐	‐	‐	‐	5.43 (0.408)
Radio	−0.07	5	1	−0.10	−0.19, 0.00	128.84	−1.94 (0.363)
Cyberbullying	0.08	5	1	0.08	0.07, 0.10	6.41	0.468 (0.388)
Newspaper	−0.08	5	1	−0.11	−0.23, 0.01	306.41	−4.26 (0.144)
Facebook	0.02	6	‐	‐	‐	‐	0.621 (0.428)
Posting political	0.07	5	‐	‐	‐	‐	0.650 (0.290)
Internet politics	0.02	5	2	0.02	0.00, 0.04	8.97	1.20 (0.111)
Piracy	0.04	6	1	0.06	−0.04, 0.15	196.11	−2.03 (0.372)
Time online	0.05	7	1	0.05	0.03, 0.06	5.62	0.228 (0.364)
Twitter	0.06	6	2	0.09	0.01, 0.17	496.18	1.80 (0.275)
Passive exposure	0.24	9	1	0.25	0.19, 0.32	121.21	1.22 (0.321)
Network attachment	0.20	6	2	0.29	0.09, 0.47	1891.34	5.01 (0.065)
General media	0.08	11	‐	‐	‐	‐	−1.46 (0.274)
Television	0.01	14	‐	‐	‐	‐	0.985 (0.365)
Cyber‐attack (intent)	0.57	3	‐	‐	‐	‐	0.827 (0.560)
*Behavioral*							
Passive exposure	0.23	4	1	0.26	0.15, 0.36	61.57	0.859 (0.427)
Active exposure	0.28	5	1	0.31	0.23, 0.38	30.97	−0.007 (0.499)

Experimental							
	***g***	***k***	**T&F**	***g***_**adjusted**_	**95% CI**	***Q***	**Egger's test *β*1 (** ***p*** **value)**
Exposure	0.08	12	2	0.04	−0.07, 0.15	32.88	2.64 (0.025)
Exposure × Aggression	0.13	4	‐	*‐*	‐	‐	0.621 (0.598)

Abbreviations: 95% CI, 95% lower and upper confidence intervals; Egger's test, test statistics and associated *p* value; *g*, Hedge's *g*; *Q*, Cochran's *Q* heterogeneity statistic; *r*, correlation coefficient; *r*_adjusted_ */g*_adjusted_ *, * correlation/Hedge's *g* adjusted after trim‐and‐fill; T&F, Trim‐and‐Fill.

## 6 DISCUSSION

### 6.1 Summary of main results

Despite a robust literature on media effects and criminological outcomes (e.g., violent cognitions and behaviors), the literature on media effects and radicalization is quite underdeveloped. The bulk of the research has been devoted to examining radical content itself, or the way in which different platforms are used as part of the strategies of radical groups to spread their messages and ideologies. Relatively little research has been carried out to examine the effects of exposure to these platforms and content on individuals' radicalization (Scrivens et al., 2020). What individual level research has been conducted, has mostly been limited to the analysis of mere passive exposure to radical content (Richards & Wood, 2020; Wolfowicz, Weisburd, et al., 2021a). It remains that little is known about differences in the effects of media‐related factors on radicalization, including with regard to mediums (e.g., television and Internet) and types of content (e.g., political, violent, and radical; Scrivens & Conway, 2019).

In this systematic review we sought to identify the different media‐related factors for which there exists quantitative estimates concerning their relationship with the two primary outcomes of radicalization: cognitive and behavioral. The primary objective of the review was to identify which factors had the strongest relative relationships with these outcomes, and to integrate the findings within the broader literature on media effects and criminological outcomes. The review identified 53 studies that met the inclusion criteria. From these studies, sufficient information was available to analyze the effects of experimentally manipulated exposure to content theorized to increase radicalization, as well as 23 different media‐related risk factors. All but two of the factors were limited to the outcome of cognitive radicalization. The factors included those pertaining to (1) mediums (Television, newspapers, radio, Internet), (2) platforms (Facebook, Twitter), (3) content (Violent, radical, political, general), (4) activities (Usage, posting, consuming), (5) attitudes (perceptions of bias and network attachment), and (6) other individual factors (e.g., technical skills and experiences of cyberbullying).

The review identified limited experimental evidence. This is not surprising given that there are serious ethical concerns with exposing participants to content that is theorized to radicalize or otherwise cause significant distress (Hassan et al., 2018). Indeed, only one of the experimental studies' treatment conditions involved exposing participants to an explicitly radicalizing piece of content in the form of an Islamic State in Iraq and Syria (ISIS) propaganda video. The other studies primarily involved exposing participants in treatment groups to news stories concerning politically charged issues, and one study included exposure to clips from action‐drama films. The results of the meta‐analysis indicate a small effect for exposure to such conditions (*g = *0.10). The results of the analysis of experimental studies indicated the presence of moderate heterogeneity. It was found that a significant source of heterogeneity was the source from which effect sizes were derived. There were significant differences in the estimates for studies which reported group means and standard deviations (or standard errors) and those which only reported results from regression models. The results of this analysis point to an underestimation, as the pooled estimate, when excluding this latter group of studies (*k *= 8) was more salient (*g = *0.19 [95% CI = 0.09, 0.29]).

A sub‐set of experimental studies examined the interaction between treatment and trait‐aggression, which is theorized to (a) increase the effects of exposure or (b) confound the effects of exposure. Indeed, the pooled estimate derived from four samples was somewhat larger than the pooled estimate for simple exposure described above. Due to the small number of effect sizes, it was not possible to investigate possible sources of heterogeneity. However, a leave‐one‐out analysis found that the removal of one study reduced heterogeneity to zero. In doing so, the size of the estimate increased, indicating a possible downward bias of the pooled results.

The results from the analysis of observational studies provide a degree of corroboration to the findings from the experimental studies. The results showed that for factors measuring exposure to mediums, irrespective of the medium, the magnitude of the effects is exceptionally small. Even when factors measure exposure to specific types of content (e.g., political or violent), irrespective of the medium, estimates remained exceptionally small. The relative magnitude of the effects for these factors were virtually indistinguishable from those that were not content‐specific. In moving down the rank‐order, only the effect sizes for seven factors were larger than *r =* 0.10, and only five of these were larger than *r =* 0.20. What distinguishes these factors from those noted above is that they were all Internet‐related factors. Among these factors, the smallest effect sizes were for online self‐censorship and posting on forums. These were followed by larger estimates for online network attachment, active seeking of radical content online, passive exposure to radical content online, consumption of ISIS news, and willingness to engage in ideologically motivated cyber‐attacks.

While caution is certainly warranted in interpreting the results, they do point to similarities with evidence from the broader field of media‐effects research on deviant outcomes which has also found that the more specific content type is to the cognition being measured, the larger the statistical relationship (Anderson & Dill, 2000; Anderson et al., 2007; Coyne et al., 2018; Gentile et al., 2004; Martins & Weaver, 2019). They also support the idea that as a medium, the Internet can have greater impact on cognitions and attitudes than television for example (Shah et al., 2001). In this regard, it has previously been found that the active posting of political content online has a greater impact on outcomes such as civic engagement than exposure to political content through other mediums (e.g., Shah et al., 2005). The potentially greater impact of the Internet over other mediums may also be explained by the various types of attitudes, emotions, and behaviors that individuals can develop toward it. For some, attachment to their online networks can be even stronger than their attachment to offline networks (Neumann, 2013). In this regard we found that a tendency toward self‐censorship, and feelings of attachment to online networks, have—relatively speaking—robust relationships with cognitive radicalization. These results demonstrate that like with offline associations and networks, individuals are susceptible to social control online as well (Wolfowicz, Weisburd, et al., 2021a). However, the analysis demonstrated that attachment to online networks is sensitive to outlier bias. As such, the true magnitude of the effect for this factor may be smaller than what was found in the primary analysis.

In this regard, in a larger systematic review and meta‐analysis of general risk and protective factors for radicalization, it was found that different forms of offline social bonds have relatively robust relationships with radicalization (Wolfowicz et al., 2020). The review found that while certain social bonds can have risk effects, others have protective effects. It has long been found that increased Internet usage can correspond with a decreased engagement with offline social bonds, especially those that can have a protective effect such as parents (Kraut et al., 1998). On the other hand, increased involvement with online networks may serve as a replacement for weak offline bonds. Weak offline social bonds, as well as stronger online network attachment have been found to increase the likelihood of exposure to online radical content (Oksanen et al., 2014). Additionally, greater online network attachment increases the likelihood of agreement with the messages of such content (Bernatzky et al., 2021).

Similar to what has been found in the broader literature on media effects and deviant outcomes (both cognitive and behavioral), even for the most important factors, effect sizes are objectively small (Valkenburg et al., 2016). However, with reference to the meta‐analysis of Wolfowicz, Litmanovitz, et al. (2021), the estimates for passive and active exposure as they pertain to cognitive outcomes would be situated at approximately the halfway point of the rank‐order of known risk factors. More striking is that for behavioral radicalization, the effect sizes would rank much higher, with estimates being quite close to those of some of the most important criminogenic factors, including thrill seeking (*r =* 0.19), low self‐control (*r = *0.28), deviant peers (*r *= 0.30), and radical cognitions themselves (*r = *0.30).

While the results indicate that traditional forms of media (even those including general political or violent content) may have essentially insignificant relationships with radicalization, they also serve to allay the minimalist perspective that Internet usage has little relationship with radicalization (Awan, 2007a). However, given the nature of the data we are unable to say what the nature of this relationship is. It is certainly possible that through social learning processes, exposure to certain types of content and associations increases radicalization. This process can be accelerated through the formation of stronger social bonds, and the adoption of group‐based norms and values. On the other hand, it is perhaps equally possible that nature of the relationship between Internet usage and radicalization is more one of amplification. That is, in the context of radicalization, Internet usage's most significant effect is in providing already cognitive radicalized or radicalizing individuals with the opportunity to engage with content and associations who can provide confirmation for their beliefs, and thereby increase the potential for engaging in radical behaviors. This assumption is analogous to evidence on media effects in which it has been found that individuals with aggressive tendencies preferentially search out violent media content, which in turn reinforces, or increases their aggression, and thereby increase the potential for engaging in aggressive behaviors (Slater et al., 2003). Similar to evidence that engagement in radical behaviors may be the biggest risk factor for radical cognitions (Moskalenko & McCauley, 2020), it is necessary to consider the possibility that radicalization is as likely to be a risk factor for exposure to radical content as exposure to radical content is for radicalization. Previously held radical and violent beliefs have been found to be predictive of engagement with radical content (Hawdon & Costello, 2020; Pauwels et al., 2020; Hawdon et al., 2019). These two possibilities are analogous to the competing paradigms of media socialization (where problematic media usage is a predictor of deviant outcomes) and media selectivity paradigm (where problematic media usage is an outcome of deviance (Slater, 2007). A third possibility that must be considered it that other risk factors for radicalization may draw individuals to engagement with radical content. These factors may be socio‐demographic, in particular age and gender, or may related to certain propensities, such as low self‐control and sensation seeking (Pauwels et al., 2020; Hawdon et al., 2019). As Felson (1996) argues, it is possible that media‐effects on violent outcomes (both cognitions and behaviors) represent imitations of the self‐control displayed by the characters that feature in the content, rather than violence in and of itself. More research is needed in order for the nature of the media‐radicalization relationship to be better understood.

The role of media effects in criminality (and radicalization) has traditionally been conceptualized, like other risk factors, as being a moderator between attitudes and behavior (Kanz, 2016). While meta‐analysis is not suitable for examining this type of theorized path model, meta‐analytic results can sometimes point to the existence of such a relationship. Generally, when analyzing related cognitive and behavioral outcomes, effect sizes for risk factors are almost always larger for the cognitive outcomes. In those instances where effects are found to be larger for the behavioral outcome, it is suggestive of the variable being a moderator of the continuity between the cognition and behavior (Ajzen & Fishbein, 1980; Bosco et al., 2015; Wolfowicz, Weisburd, et al., 2021a). In this regard, our review found that the effect sizes for passive and active exposure to online radical content on behavioral radicalization were effectively identical to those for cognitive radicalization. While in and of itself this does not tell us much about the nature of the relationship, it does provide indication that it is worthwhile to further explore the potential moderating effects of these factors.

Overall, a degree of consistency can be seen between our findings and those concerning media‐effects for violent cognitions and behaviors more generally. However, the current state of the body of evidence still has some way to go before we can make any claims of causality, which can currently not be made. Taken together, the results of this review should serve as a source of direction for research. In recent years there has been significant growth in criminological applications to the study of radicalization and terrorism with respect to a wide range of issues. To a large degree it can be said that the degree of overlap between radicalization/terrorism and other forms of deviance is greater than the degree of divergence (LaFree et al., 2020). With this being the case, we hope that this review will serve as a source of inspiration for better integrating the rich study of media effects into the radicalization research agenda.

### 6.2 Overall completeness and applicability of evidence

When assessing the results of the current review against the broader media‐effects literature, the evidence is only partially complete. As is often the case with systematic reviews, our results also highlight some of the gaps in the body of knowledge. Perhaps most glaring is the lack of experimental studies. While the results from our analysis on four experimental studies (which included twelve individual samples) generally conform to findings from the broader literature that laboratory experiments have small, and inconsistent effects on violent cognitions, more studies are required before any firm conclusions can be drawn. Similarly, the literature demonstrates a lack of longitudinal studies. Especially given competing hypotheses of media socialization and selectivity, establishing temporal ordering of the exposure factor, and controlling for previous levels of the cognitive or behavioral outcome of interest, is essential for establishing estimates that are more robust to biases (Hopf et al., 2008). Moreover, the results of the review highlight the lack of case‐control studies comparing violent and non‐violent extremists (cognitive and behavioral radicalization). These types of studies are needed to identify what types of media‐related factors, activities, and behaviors may differentiate between these two groups. The absence of evidence derived from this type of inquiry represents a significant hindrance to the development of more evidence‐based policies and practices (Freilich & LaFree, 2016; Scrivens et al., 2020).

In addition, our review identified several factors for which only a single effect size was found, and as such no meta‐analysis was conducted. With regard to mediums, single effect sizes were found for Video‐game play (Eyal et al., 2006), Music (Manzoni et al., 2019), and YouTube usage (Frissen et al., 2019). We also found single effect sizes for specific aspects of Internet usage, namely network structure characteristics such as network heterogeneity (Zhu et al., 2020), and user‐profile level activity such as receiving or giving of “likes” (Goede et al., 2019). Regarding network structure characteristics, one of the most frequently referred to mechanisms of online radicalization is that of the echo chamber, which describes an insular, isolated, and homogenous network. There is now an extensive literature that has found statistically significant relationships between several different network‐level characteristics (such as density, homogeneity, tie strength etc.) and a range of cognitions and behaviors (Chancellor & De Choudhury, 2020). However, as demonstrated by our results, there is little evidence concerning echo‐chamber related factors and individual radicalization outcomes (Macdonald & Whittaker, 2019). Regarding specific aspects of online behaviors, there is also a growing body of research that demonstrates that factors such as posting frequency, sharing, and the receiving of likes and comments, may be highly predictive of a range of cognitive and behavioral outcomes (Chancellor & De Choudhury, 2020). While some studies have demonstrated the usefulness of such factors in improving automated detection of radicalization online (e.g., Nouh et al., 2019; Wolfowicz, Perry, et al., 2021), our review highlights a lack of evidence concerning their relationship with radicalization itself.

Overall, it is evident that there are additional media‐related risk factors for radicalization for which there is currently insufficient evidence to enable a meaningful synthesis. Given the potential importance of these factors, as well as those that were analyzed These factors represent areas that demand further research.

### 6.3 Quality of the evidence

Like other research on risk factors that relies primarily on cross‐sectional studies, the nature of the relationships examined in the study can best be classified as putative risk factors. That is, whilst the effects demonstrate correlations in the theorized direction, they lack confirmation of the temporal ordering needed for classification as risk or protective factors (Wolfowicz, Weisburd, et al., 2021a). As a reflection of the quality of the studies in the literature, this represents a limitation on the types of inferences that can be made considering the current state of the evidence. Indeed, the review included one longitudinal study that did control for previously held beliefs and mediated exposure to violence and found that in doing so, there was no relationship between exposure and later radical attitudes (Gvirsman et al., 2016). Whilst conclusions cannot be drawn from a single longitudinal study, previous research on media effects in criminology has found that when controlling for such factors, media effects are substantially reduced when compared to cross‐sectional studies (Anderson et al., 2010).

Relatedly, the included studies rely on self‐reports of media exposure and usage. It has often been noted that individuals have difficulties in accurately recalling their actual media use and consumption, which can often mean significant over and under reporting (Prior, 2009a, 2009b, 2012). While these are sometimes the best measures of individual exposure that are available (Slater, 2004), they do introduce a source of potential bias.

As with all studies dealing with observational data, the most important quality criteria pertain to measures of the dependent and independent variables, appropriate sampling, and appropriate comparisons made statistically (Murray et al., 2009).

#### 6.3.1 Dependent variables

With respect to outcome measures, most studies did not use validated measures of cognitive radicalization. However, validated measures of cognitive radicalization are relatively recent developments in the literature, and the types of measure used in the included studies are widely considered to be appropriate (Schmid, 2017). Measures of behavioral radicalization were measured dichotomously and primarily based on self‐reports. While self‐reports provide an interesting perspective on radical behaviors, there is considerable distance between these behaviors and "terrorism” (McCauley & Moskalenko, 2017).

#### 6.3.2 Independent variables

The nature of the independent variables examined in this review are like those that are examined in the broader study of media‐effects. That is, the independent variables capture self‐reports pertaining to media usage and consumption. While these types of measures are quite standard in media‐effects research, they are sensitive to under and over estimations of exposure time (Prior, 2009a, 2009b, 2012). Additionally, self‐reports are sensitive to memory recall bias, especially when the reports pertain to topical content toward which respondents possess differential levels of interest (Donohew et al., 1998; Slater, 2004).

#### 6.3.3 Sampling

The included studies employed appropriate sampling procedures for observational research. However, given a lack of nationally representative surveys, we were unable to assess differences in effect sizes based on sampling strategies.

#### 6.3.4 Statistical approaches

Studies generally employed appropriate statistical techniques for identifying the strength of the relationship between measures. In this regard, another important consideration is whether the factors have a theoretically plausible relationship with the outcome (Murray et al., 2009). Indeed, most factors analyzed in this study, especially those with the largest estimates (as per the above discussion), are theoretically derived factors which have plausible relationships with radicalization outcomes. However, the studies did not always control for important confounding factors, especially those known to be especially relevant for media‐effects such as self‐control, trait aggression, and gender. It has previously been suggested that bivariate correlations between violent media consumption and violent outcomes may simply represent gender effects, or the effects of trait aggression, which is also highly correlated to gender (Ferguson, 2015). Additionally, these and other factors may impact differential exposure to media (Savage & Yancey, 2008). Given the nature of the data, we were unable to analyze effects adjusted for such factors and we were unable to account for this potential source of bias. As such, whilst the effects identified in this review certainly follow the theorized direction, it is also entirely possible that more radical individuals are simply more likely to engage in the types of media behaviors analyzed.

#### 6.3.5 Publication bias and sensitivity of the results

Publication bias and sensitivity analysis were limited to factors for which there were more than three effect sizes. For most factors, there was no evidence of significant publication bias or sensitivity to outliers. For those factors for which these analyses indicated changes to the pooled estimates, the differences were exceptionally small and do not change the substantive findings. However, for one factor, network attachment, the results are inconclusive. The leave‐one‐out analysis indicated that one study significantly contributed to an inflated estimate. In removing this study, the pooled estimate was reduced from *r = *0.20 to *r = *0.07. On the other hand, the Trim‐and‐Fill analysis imputed two missing effect sizes to the right of the pooled estimate, increasing the estimate to *r = *0.29. These results indicate that until additional effect sizes can be synthesized, the results for this factor should be interpreted cautiously. The only other factor for which there was significant evidence of publication bias was for exposure to radicalizing exposure in experimental studies. Here, the Trim‐and‐Fill analysis indicated a small reduction in the effect size.

### 6.4 Limitations and potential biases in the review process

#### 6.4.1 Review process

We acknowledge that there are studies that may provide important evidence concerning the risk and protective factors for radicalization that were not included in this review on account of their outcome measures failing to meet the review's inclusion criteria. For example, some studies are known to assess “willingness to die for a cause/group.” However, as described above, such studies were excluded since a willingness to die does not necessarily indicate a willingness to use violence against others (e.g., Bélanger et al., 2014). Other studies have examined the effects of different forms of media consumption on outcomes such as racism and hate‐speech (Soral et al., 2020). Yet others, such as Machackova and Šerek (2017), analyzed how online political activity predicted the acceptance of “non‐conventional” activism, which included a vague reference to “run‐ins with authorities.” We do not believe that the review's results are biased because of having excluded such studies. Rather it means that our results are based on a more homogenous set of outcomes and should therefore be more robust from a meta‐analysis perspective. However, we can note here that the findings from these studies demonstrate a significant degree of overlap with our results.

It is also important to highlight the fact that several of the meta‐analyses performed in this review are based on a very small number of effect sizes (as few as two) and some of those with a greater number of effect sizes demonstrate significant heterogeneity. As such, caution must be used in drawing inferences from our analyses and interpreting our results.

Another potential limitation of this review is language. While it is common to assume that the most important studies are at least indexed in databases in English, we cannot discount the possibility that studies in other languages exist that we were unable to identify. Indeed, as our results found a handful of studies examining samples in Asia, we suspect that additional studies from these countries may exist in local languages. Future attempts to synthesize the body of evidence may consider dedicating more significant resources to attempting to identify studies published in other languages.

Whilst we encourage replication, we acknowledge that the authors' familiarity with the literature may have impacted the number of studies that passed through the different screening stages. As such, while a replication will likely reach similar substantive findings, differences in inclusions and exclusions at different stages would be expected.

### 6.5 Agreements and disagreements with other studies or reviews

To date, only one other review has specifically focused on the media‐radicalization relationship, placing a specific focus on the Internet. That review was unable to provide any sort of quantitative synthesis however, and it also included studies that were not individual‐centric. Nevertheless, the conclusions of that review were that Internet usage can be a source of risk for radicalization (Hassan et al., 2018), which receives support by the results of the current review. In a larger review of risk and protective factors for radicalization in OECD countries, Wolfowicz, Litmanovitz, et al. (2021) analyzed three factors relevant to the current study: Time spent on the Internet, positing of political opinions on the Internet, and exposure to radical content on the Internet. The factors were situated approximately in the middle of the rank order of risk factors and the size of the estimates identified for those factors are comparable to those identified in the current review for similar factors. In this regard, the effect sizes of violent media on violent outcomes more generally, usually ranks similarly, around the middle of a rank order of other risk factors (Saleem & Anderson, 2012).

**Figure 5:**
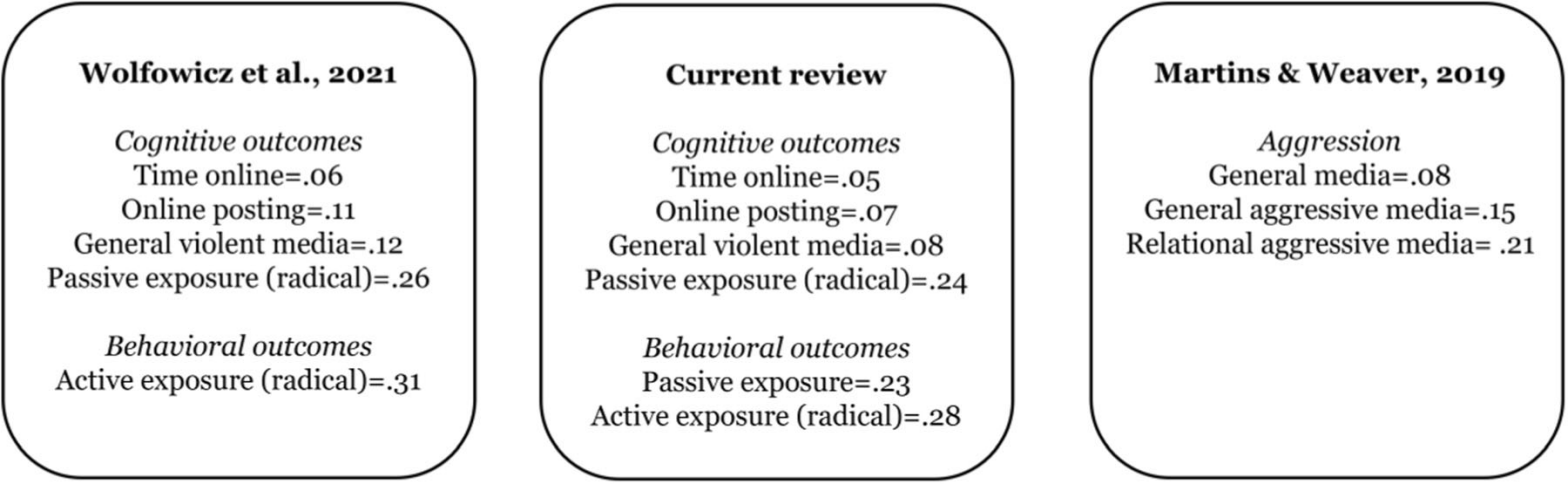
A comparison of the current review with Wolfowicz, Litmanovitz, et al. (2021) and Martins and Weaver (2019)

Evidence from the broader field of research on media effects for outcomes related to violent cognitions and behaviors demonstrates some degrees of agreement with the results of the current review. First, Martins and Weaver (2019) find that the degree of specificity between the type of content and aggression has a significant impact on effect sizes, similar to the results of our review. Here, they found that while exposure to general media had an effect of *r = *0.08, exposure to general violent media had an effect of *r = *0.15, and relational violent media (specific to aggression outcomes measured) had an effect of *r = *0.21. Figure 5 provides a juxtaposition of the results from Wolfowicz, Litmanovitz, et al. (2021) and Martins and Weaver (2019) with the current review.

Moreover, in recent years, been primarily focused on the effects of video games. While the current review was unable to examine the effects of video games, one similar finding is that the effects of violent video games are stronger for behavioral than cognitive outcomes of aggression (Ferguson et al., 2020). Another area of overlap relates to the relative magnitude of media effects compared to other known risk factors. In this regard, whether relating to aggressive cognitions or behaviors, media effects generally rank at about the halfway mark in rank orders of other commonly examined risk factors (Comstock et al., 2014).

## 7 AUTHORS' CONCLUSIONS

### 7.1 Implications for practice and policy

The review only identified two factors for behavioral radicalization, passive and active exposure to radical content, and the quality of the evidence is poor. Nevertheless, the relative magnitude of the effects suggests that relationships for these factors are relatively robust. The findings therefore support a policy focus on the Internet that views it as a potential source of risk. In practice, problematic Internet usage, and engagement with radical content online in particular, should be investigated as being added as a risk factor item in risk assessment. It would seem intuitive for interventions to aim at reducing the availability of radical content online. This is especially important given that a sizable proportion of individuals, especially youth and young adults, may actively seek out, engage with, or accidentally come to be exposed to radicalizing content (e.g., Frissen, 2021; Grizzle & Tornero, 2016). Simultaneously, whether through counter‐narratives or other means, it is important to seek ways to reduce the influence of radical content. However, in developing strategies it is important to weigh other considerations. In this regard, it is possible that by permitting individuals engagement with radical content online, it may provide them with a non‐violent outlet to voice their grievances. Additionally, the monitoring of online activities is an important aspect of counter‐terrorism efforts and may provide an opportunity for early prevention (Wolfowicz, Weisburd, et al., 2021a). Whether it pertains to the Internet or other forms of media, policy efforts to control access and availability to content, as well as the leveraging of the Internet for surveillance and prevention efforts, should be carefully considered against possible impingements on free speech and civil liberties (Hasisi et al., 2019). It is therefore important for policy makers to support research than can underpin the development of more evidence‐based policies and practices, especially case control research that can serve to identify factors that differentiate between violent and non‐violent radicals (e.g., Scrivens et al., 2021; Wolfowicz, Weisburd, et al., 2021a).

Currently, the most common strategies to combat the risks of Internet‐based radicalization are (1) content removal, (2) counter‐narratives, and (3) digital literacy. However, the evidence based for each of these approaches is lacking (Zeiger & Gyte, 2020). Additionally, in some cases there is evidence of the potential for iatrogenic effects. Below we discuss these issues in brief and highlight how the results of our analysis relate to them, as well as potential new areas that should potentially be considered.

With respect to the “takedown” of content, profiles, and pages, government agencies actively remove radicalizing content or compel IT companies to carry out such removal. In recent years, companies such as Twitter have taken an active approach to the removal of radical content and profiles. However, this approach has been criticized for failing to reduce access, as new profiles quickly replace those removed (Conway et al., 2019). Additionally, radical groups may use content removal as being demonstrative of their claims against governments and societies as being discriminatory toward their group (Neumann, 2013). Moreover, by removing radicals from more open platforms (such as Twitter), they may move to platforms that are more difficult to monitor (such as Telegram). Furthermore, content filtering and removal is usually driven by algorithms, and inevitably leads to the removal of legitimate content and may impinge on civil liberties (Hasisi et al., 2019). There is indeed evidence that following a crackdown on ISIS‐related Twitter accounts, the group has migrated to Telegram. And while their reach through Telegram may be significantly smaller than it was on Twitter, it is believed that the move to the platform has led to a more radicalized and committed following (Conway et al., 2019). Given that the magnitude of the estimates for radical content exposure are objectively only small to moderate, and the potential for confounding is more likely to be associated with overestimation rather than underestimation, policy makers should carefully consider the costs and benefits of takedown policies.

With respect to counter‐narratives, these are meant to balance out. According to social learning theory, it is the balance of definitions favoring or dissenting from a particular belief or behavior that determine whether an individual will adopt the given belief or behavior (Akers, 1998). However, as identified by a recent Campbell Collaboration systematic review, the evidence on the effects of counter‐messaging and counter‐narratives is still underdeveloped (Carthy et al., 2020). There is currently mixed evidence concerning potential iatrogenic effects as well. While the results of a recent experimental study found that counter‐messaging can increase radicalization for those who already hold radical attitudes (Bélanger et al., 2020), a much larger study carried out in cooperation with Facebook found no evidence of counter‐messaging increasing radicalization (e.g., Saltman et al., 2021). As such, further research on developing counter‐narratives is needed. One possible direction that the results of this review would support, is that like counter‐radicalization strategies that target underlying risk factors as opposed to radical beliefs themselves (Wolfowicz et al., 2020), counter‐narratives should target alternative, risk‐related topics. In addition, counter‐narrative strategies need to take into consideration how elements of the Internet itself, such as personalization algorithms, may be leveraged to reduce access to radicalizing content, and facilitate the distribution of counter‐messaging (Rieger et al., 2019). Another potential avenue is to take a more positive‐oriented approach. Recent meta‐analytic evidence shows that exposure to pro‐social media can serve as a protective factor against a range of negative outcomes, including aggressive cognitions and behaviors (Coyne et al., 2018).

With respect to digital literacy, which figures prominently in the literature, there is less evidence. Despite reason to believe that digital literacy can reduce susceptibility to radical content online (Grizzle & Tornero, 2016), the little quantitative evidence that exists is not promising. For example, Alfida et al. (2019) study of Indonesia students found no correlation between digital literacy, understanding about the nature and dangers of Internet‐facilitated radicalization, and radicalization itself. We note that our results find a small risk effect for technological skills and radicalization. Of course, this result must be approached with caution given the quality of the evidence from which it is derived. Like other risk factors that pertain to the development of individual skills, further investigation into the effects of digital literacy is needed, especially given its focus in practice and policy (Wolfowicz et al., 2022).

The results of the review point to additional areas that may be considered relevant for practice and policy, in particular combatting Internet addiction. In this regard, the review found that attachment to online networks, which is related to Internet addiction, is associated with increased risk for cognitive radicalization. While it is important to interpret these results with caution in light of the low quality of the studies, Internet addiction has been found to be associated with a range of negative psychological consequences (Lopez‐Fernandez & Kuss, 2020). As policy makers are already seeking to combat Internet addiction as a general societal problem (Lopez‐Fernandez & Kuss, 2020), there may be cross‐cutting benefits in countering radicalization as well. So too, the review found that cyberbullying victimization, and self‐censorship online are associated with small risk estimates. While again it is important to temper interpretations of the results against the low quality of the evidence, issues of cyberbullying are already part of the broader agenda of social issues for many countries and may represent an area with cross‐cutting implications.

### 7.2 Implications for research

Overall, the results of this review serve to integrate the study of media effects and radicalization into the broader meta‐analytic contributions to the study of media effects on other criminal and criminal‐analogous outcomes. The findings demonstrate a significant degree of overlap with research on outcomes such as violent and aggressive cognitions and behaviors, as well as cognitions and behaviors pertaining to specific types of crime. However, unlike other comparisons between related cognitive and behavioral outcomes, the magnitude of the effects between cognitive and behavioral radicalization for the most relevant factors are almost identical. As such, further research into the role of the Internet in radicalization is certainly justified and needed.

At the same time, the review highlights a relative lack of quantitative research in general and identifies a relatively limited scope of factors examined to date. There is a need to broaden the scope of the types of media‐related factors that are analyzed, and the methods used in their research. This is demonstrated by several single effect sizes identified in the review. Research also needs to consider other research designs which can perhaps be more informative. In particular, case‐control studies comparing radicalized and non‐radicalized individuals, or behaviorally radicalized with cognitively radicalized individuals may serve to identify what types of media effects may differentiate between these groups. This is the type of research that may serve to improve the effectiveness of online surveillance technologies and prevention efforts more generally. Unfortunately, to date, there are a very limited number of studies that have taken this approach (e.g., Scrivens et al., 2021; Wolfowicz, Hasisi, et al., 2021).

Additionally, social network analysis is a promising methodology for examining a range of factors, including those pertaining to the theorized effects of echo chambers. In this regard, a recent study found that certain types of network structure characteristics, such as network insularity, may be predictors of radicalization. While there are a number of studies that employ network analysis at the group level, more research is needed at the individual level (Wolfowicz, Weisburd, et al., 2021a).

There are of course a number of systematic issues that should lead us to question the strength and direction of the factors examined in this review. As stated above, differential engagement with different types of media usage, and its influence, may reflect confounding factors such as age, gender, and self‐control. As has been demonstrated by inquiries into media‐effects for outcomes some as aggression, accounting for these confounders can significantly reduce the magnitude of observed bivariate relationships. Indeed, the likelihood of exposure to radical content online has been found to be predicted by such factors (Hawdon et al., 2019). That the studies included in this review generally did not do so, should serve as a call to researchers to ensure that future work accounts for such issues.

Another issue that researchers should consider is how radicalization is measured. While it is true that more validated instruments have been developed only recently, using these instruments will help to ensure a greater degree of comparability. It is likely that the different types of measures used by the included studies serve to account for at least some of the observed heterogeneity, which could stem both from differences in the constructs being captured and in measurement scaling. Whilst we do not recommend any particular instrument over another, examples from studies included in this review are SyFor (Bhui et al., 2014) and ARIS (Moskalenko & McCauley, 2009).

It is also of note that whilst only small, there are statistically significant differences in the effects for both general media usage (for cognitive radicalization) and radical content consumption (for behavioral radicalization) between regions, with the effects for both factors being larger for European studies. Previous research has found that only a small number of risk factors for radicalization display regional heterogeneity (Wolfowicz, Hasisi, et al., 2021). Future research may find benefit in examining the issue from a cultural perspective, which may consider how different cultures relate to media usage more generally or use media differently. It may also be the case that the differences simply reflect different frequencies of media usage, which can also be attached to culture. These are issues that future research should take into consideration or otherwise could serve as important lines of inquiry in and of themselves.

Relatedly, Internet usage continues to increase, in part due to the accessibility offered by mobile devices. Increased usage may increase the likelihood for exposure to radical content, a function of routine activities (Hawdon et al., 2019), whereas more frequent exposure may increase the potential influence that such content has on its consumers, a function of social learning. Our analysis found a marginally significant effect for year of data collection on passive exposure to radical content, in which more recent studies are associated with larger effects. Future research should consider how the year of data collection may influence results, and how results may reflect trends in media usage more generally.

Lastly, it is important to consider how the development of longitudinal research may impact our understanding of media effects in radicalization. In one longitudinal study, by Gvirsman et al. (2016), mediated exposure to violence did not influence radicalization when controlling for prior exposure and previously held radical attitudes. However, in another study, by Zhu et al. (2020), online political/radical activity as well as online network heterogeneity did predict radical behaviors, even when controlling for previous online activity and radical behaviors. More longitudinal studies are needed to clarify the nature and magnitude of the effects of these factors. In this respect, the current state of the literature is like the body of evidence concerning risk factors for radicalization more generally, which is still reliant primarily on cross‐sectional designs (Wolfowicz, Weisburd, et al., 2021a).

We believe that the current evidence concerning media effects in radicalization is quite clear in at least one respect: That media exposure alone is unlikely to lead to radicalization. Indeed, there are very few cases of terrorists who were wholly radicalized by the media (Meleagrou‐Hitchens et al., 2017). Even in cases where the Internet has apparently played an important role in radicalization processes, offline processes have been found to be more important (von Behr et al., 2013). Indeed, there are over a hundred different risk factors for radicalization that are not related to media (Wolfowicz, Weisburd, et al., 2021a). It is therefore important for research to consider the interplay between online and offline exposures to radicalizing messages. That is, exposure to radicalizing content, especially in instances where it contributes to risk, may not occur in a vacuum. It may be related to an individual's predispositions, or other aspects of their lives, including meeting new peers who themselves may hold radical attitudes or share such content with them.

The current evidence suggests that the field could benefit greatly from recent contributions from criminology to the field of radicalization and terrorism research, including the application of both theoretical and methodological frameworks (LaFree et al., 2020). As demonstrated by our results, theories such as Social Learning Theory and Social Control Theory may provide prisms through which to test hypotheses. Through these and other prisms, researchers may seek to identify if media effects follow the theorized paths to radicalization. For example, does exposure to certain types of content impact radicalization through its effects on identity? Does exposure to certain types of content impact radicalization through its effects on grievances? Does engagement with certain types of media impact social bonds? While such factors may sometimes only have small relationships with radicalization (Wolfowicz, Weisburd, et al., 2021a), they may operate as key moderators of the media‐radicalization relationship. Similarly, researchers should also consider what the key outcomes are of media's effects on radicalization. As stated above, cognitive radicalization rarely leads to behavioral radicalization (McCauley & Moskalenko, 2017). However, it could be associated with other negative outcomes. In this regard, exposure to violent media has been seen to increase the risk of violent cognitions, which can negatively affect psychological well‐being, including factors such as life‐satisfaction and attachment (Huesmann, 2007; Jahangir et al., 2014; Reinecke & Oliver, 2016). It is of note that such factors have robust relationships with radicalization as well (Wolfowicz, Weisburd, et al., 2021a).

We believe that the results of this review, which provide modest estimates for media effects on radicalization, but which suffer from numerous potential sources of bias, supports the assertion that “The power of radical messages online and offline and the powerful discourses brought about by recruiters should neither be overestimated or underestimated” (Pauwels & Hardyns, 2018, p. 36).

## ROLES AND RESPONSIBILITIES

- Content: Michael Wolfowicz, Badi Hasisi, David Weisburd
- Systematic review methods: Michael Wolfowicz, David Weisburd
- Statistical analysis: Michael Wolfowicz
- Information retrieval: Michael Wolfowicz, Badi Hasisi

## SOURCES OF SUPPORT

United States Department of Homeland Security (Grant Number: 140D0418C0011). The Federmann Cyber Security Research Center.

## DECLARATIONS OF INTEREST

The review's authors authored one presently unpublished study that was included in the current review.

## PLANS FOR UPDATING THE REVIEW

The lead author will be responsible for ensuring that the review is updated in 3–5 years from the date of its first publication if no comparable review is published by that time.

## DIFFERENCES BETWEEN PROTOCOL AND REVIEW

The review deviated from the protocol with respect to the databases that were included in the final searches. In particular, instead of search in International Political Science Abstracts (ISPA), searches were performed in Worldwide Political Science Abstracts, which includes ISPA.

The review also deviated from the protocol with respect to the publication bias analysis. While the protocol stated the Fail‐Safe *N* test would be conducted, this approach was changed to Egger's regression following comments from the reviewers.

Another deviation from the protocol pertained to the way in which meta‐regression and moderator analyses were applied. In the protocol it was stated that for categorical variables, meta‐regression would be used, and where found to be statistically significant, this would be followed up with moderator analysis. Following comments from a Campbell Collaboration editor, this approach was abandoned. Instead, wherever there was sufficient data, all categorical variables were investigated using moderator analysis.

In addition, the review included some ad‐hoc analyses that were not noted in the protocol. Relatedly, it was not possible to assess the impact of some of the study level characteristics listed in the protocol due to an insufficient number of studies/effect sizes for each category. This was the case for “Different measures of outcomes” and “Different types of studies.”

## Supporting information

Supporting information.

## Footnotes

1 A video‐game tournament using the FIFA soccer game was conducted in Malmo, Sweden by the NGO Kommon‐Ground as a pilot CVE intervention. The goal of the intervention was to promote intergroup cooperation and other prosocial factors.

2 In the review protocol we acknowledged the potential for studies authored by the review's authors to be included.

3 As with the initial screenings, some studies failed to meet multiple inclusion criteria.

4 Pre‐registration of studies, such as in the form of a research protocol, are rare in these fields. However, pre‐registration helps to reduce this form of bias as researchers would prespecify which factors are to be investigated. Pre‐registration of observational research has increasingly received encouragement to promote more robust and open science (Williams et al., 2010).

5 Alternative operationalizations of the dependent variable can potentially lead to different results.

6 As per the protocol, results from experimental studies were to be calculated and reported as Hedge's *g*. However, four of the effect sizes were calculated from unstandardized regression coefficients. On account of this, we also report here that the reported estimate is equivalent to *r* = 0.04 [95% CI = −0.02, 0.09]. Similarly, the estimate for the interaction between exposure and trait aggression was *r* = 0.06.
