# Supplementary material for: What are the effects of different elements of media on radicalization outcomes? A systematic review
Source: Campbell Syst Rev. 2022 Jun 8;18(2):e1244. doi: 10.1002/cl2.1244 (PMC9175065; doi:10.1002/cl2.1244)
Supplement: Supplementary file 1 — Supporting information. [file CL2-18-e1244-s001.docx]

# Online supplements

**Worldwide Political Science Abstracts, ProQuest [1973-]**

**Search conducted in August 2020**

**Total results: 2,048**

(ab(radical* OR extrem* OR terror* OR action OR “politically motivated*” OR ideological*) AND ab(media OR technology OR internet OR online) AND ab(quantitative OR empirical OR survey OR regression OR multivariate OR correlation OR experiment* OR manipulat* OR coefficient* OR covariat* OR longitudinal OR paramet* OR predict* OR questionnaire* OR sampl* OR standard deviation* OR statistic* OR variable* OR variance)) OR (ti(radical* OR extrem* OR terror* OR action OR “politically motivated*” OR ideological*) AND ti(media OR technology OR internet OR online) AND ti(quantitative OR empirical OR survey OR regression OR multivariate OR correlation OR experiment* OR manipulat* OR coefficient* OR covariat* OR longitudinal OR paramet* OR predict* OR questionnaire* OR sampl* OR standard deviation* OR statistic* OR variable* OR variance)) OR (if(radical* OR extrem* OR terror* OR action OR “politically motivated*” OR ideological*) AND if(media OR technology OR internet OR online) AND if(quantitative OR empirical OR survey OR regression OR multivariate OR correlation OR experiment* OR manipulat* OR coefficient* OR covariat* OR longitudinal OR paramet* OR predict* OR questionnaire* OR sampl* OR standard deviation* OR statistic* OR variable* OR variance))

**Worldwide Political Science Abstracts, ProQuest [1973-]**

**Search conducted in April 2022**

**Total results: 460**

((ti(radical* OR extrem* OR terror* OR action OR "politically motivated" OR ideological*) OR ab(radical* OR extrem* OR terror* OR action OR "politically motivated" OR ideological*) OR mainsubject(radical* OR extrem* OR terror* OR action OR "politically motivated" OR ideological*)) AND (ti(media OR technology OR internet OR online) OR ab(media OR technology OR internet OR online) OR mainsubject(media OR technology OR internet OR online)) AND (ti(quantitative OR empirical OR survey OR regression OR multivariate OR correlation OR experiment* OR manipulat* OR coefficient* OR covariat* OR longitudinal OR paramet* OR predict* OR questionnaire* OR sampl* OR &"standard deviation" OR statistic* OR variable* OR variance) OR ab(quantitative OR empirical OR survey OR regression OR multivariate OR correlation OR experiment* OR manipulat*OR coefficient* OR covariat* OR longitudinal OR paramet* OR predict* OR questionnaire* OR sampl* OR "standard deviation" OR statistic* OR variable* OR variance) OR mainsubject(quantitative OR empirical OR survey OR regression OR multivariate OR correlation OR experiment* OR manipulat* OR coefficient* OR covariat* OR longitudinal OR paramet* OR predict*

OR questionnaire* OR sampl* OR "standard deviation" OR statistic* OR variable* OR variance)) AND (pd<20200901))) NOT ((ab(Radical* OR Extrem* OR Terror* OR Action OR Politically motivated* OR Ideological*) AND ab(Media OR Technology OR Internet OR Online) AND ab(Quantitative OR Empirical OR Survey OR Regression OR Multivariate OR Correlation OR Experiment* OR Manipulat* OR coefficient* OR covariat* OR longitudinal OR paramet* OR predict* OR questionnaire* OR sampl* OR standard deviation*, OR statistic* OR variable* OR variance)) OR (ti(Radical* OR Extrem* OR Terror* OR Action OR Politically motivated* OR Ideological*) AND ti(Media OR Technology OR Internet OR Online) AND ti(Quantitative OR Empirical OR Survey OR Regression OR Multivariate OR Correlation OR Experiment* OR Manipulat* OR coefficient* OR covariat* OR longitudinal OR paramet* OR predict* OR questionnaire* OR sampl* OR standard deviation*, OR statistic* OR variable* OR variance)))

**Social Sciences Citation Index (SSCI)-Clarivate, ISI Web of Science [1965-]**

**Search conducted in August 2020.**

**Total results: 54**

TS= ((radical* OR extrem* OR terror* OR action OR “politically motivated*” OR ideological*) AND (media OR technology OR internet OR online) AND (quantitative OR empirical OR survey OR regression OR multivariate OR correlation OR experiment* OR manipulat* OR coefficient* OR covariat* OR longitudinal OR paramet* OR predict* OR questionnaire* OR sampl* OR standard deviation* OR statistic* OR variable* OR variance))

**Social Sciences Citation Index (SSCI)-Clarivate, ISI Web of Science [1965-]**

**Search conducted in April 2022.**

**Total results: 9,514**

*Step 1****:*** TS= ((radical* OR extrem* OR terror* OR action OR “politically motivated*” OR ideological*) AND (media OR technology OR internet OR online) AND (quantitative OR empirical OR survey OR regression OR multivariate OR correlation OR experiment* OR

manipulat* OR coefficient* OR covariat* OR longitudinal OR paramet* OR predict* OR questionnaire* OR sampl* OR standard deviation* OR statistic* OR variable* OR variance))

*Step 2*: TS= ((radical* OR extrem* OR terror* OR action OR “politically motivated*” OR ideological*) AND (media OR technology OR internet OR online) AND (quantitative OR empirical OR survey OR regression OR multivariate OR correlation OR experiment* OR

manipulat* OR coefficient* OR covariat* OR longitudinal OR paramet* OR predict* OR questionnaire* OR sampl* OR standard deviation* OR statistic* OR variable* OR variance))

*Step 3:* TS=(radical* OR extrem* OR terror* OR action OR "politically motivated*" OR ideological*)

*Step 4:* TS=(media OR technology OR internet OR online)

*Step 5:* TS=(quantitative OR empirical OR survey OR regression OR multivariate OR correlation OR experiment* OR manipulat* OR coefficient* OR covariat* OR longitudinal OR paramet* OR predict* OR questionnaire* OR sampl* OR standard deviation* OR statistic* OR variable* OR variance)

*Step 6:* #3 AND #4 AND #5

**Criminal Justice Abstracts, Political Science Abstracts [2000-], Violence and Abuse Abstracts [1995-], Open Dissertations-EBSCO**

**Searched conducted in August 2020**

**Search conducted on the four databases jointly**

**Total results: 9,113**

(TI=Radical* OR Extrem* OR Terror* OR Action OR Politically motivated* OR Ideological* ) AND TI ( Media OR Technology OR Internet OR Online ) AND TI ( Quantitative OR Empirical OR Survey OR Regression OR Multivariate OR Correlation OR Experiment* OR Manipulat* OR coefficient* OR covariat* OR longitudinal OR paramet* OR predict* OR questionnaire* OR sampl* OR standard deviation*, OR statistic* OR variable* OR variance ) OR AB ( Radical* OR Extrem* OR Terror* OR Action OR Politically motivated* OR Ideological* ) AND AB ( Media OR Technology OR Internet OR Online ) AND AB ( Quantitative OR Empirical OR Survey OR Regression OR Multivariate OR Correlation OR Experiment* OR Manipulat* OR coefficient* OR covariat* OR longitudinal OR paramet* OR predict* OR questionnaire* OR sampl* OR standard deviation*, OR statistic* OR variable* OR variance)

**Violence and Abuse Abstracts [1995-], Open Dissertations-EBSCO**

**Search conducted in April 2022**

**Search conducted on the two databases jointly**

**Total results: 458**

*Step 1:* TI ( Radical* OR Extrem* OR Terror* OR Action OR Politically motivated* OR Ideological* ) AND TI ( Media OR Technology OR Internet OR Online ) AND TI (Quantitative OR Empirical OR Survey OR Regression OR Multivariate OR Correlation OR Experiment* OR Manipulat* OR coefficient* OR covariat* OR longitudinal OR paramet* OR predict* OR questionnaire* OR sampl* OR standard deviation*, OR statistic* OR variable* OR variance)

*Step 2:* AB ( Radical* OR Extrem* OR Terror* OR Action OR Politically motivated* OR Ideological* ) AND AB ( Media OR Technology OR Internet OR Online ) AND AB (Quantitative OR Empirical OR Survey OR Regression OR Multivariate OR Correlation OR

Experiment* OR Manipulat* OR coefficient* OR covariat* OR longitudinal OR paramet* OR predict* OR questionnaire* OR sampl* OR standard deviation*, OR statistic* OR variable* OR variance)

*Step 3:* S1 OR S2

*Step 4:* TI ( Radical* OR Extrem* OR Terror* OR Action OR Politically motivated* OR Ideological* ) AND TI ( Media OR Technology OR Internet OR Online ) AND TI (Quantitative OR Empirical OR Survey OR Regression OR Multivariate OR Correlation OR Experiment* OR Manipulat* OR coefficient* OR covariat* OR longitudinal OR paramet* OR predict* OR questionnaire* OR sampl* OR standard deviation*, OR statistic* OR variable* OR variance

) OR AB ( Radical* OR Extrem* OR Terror* OR Action OR Politically motivated* OR Ideological* ) AND AB ( Media OR Technology OR Internet OR Online ) AND (Quantitative OR Empirical OR Survey OR Regression OR Multivariate OR Correlation OR Experiment* OR Manipulat* OR coefficient* OR covariat* OR longitudinal OR paramet* OR predict* OR questionnaire* OR sampl* OR standard deviation*, OR statistic* OR variable* OR variance)

*Step 5:* TI ( radical* OR extrem* OR terror*OR action OR "politically motivated" OR ideological* ) OR AB ( radical* OR extrem* OR terror* OR action OR "politically motivated" OR ideological* ) OR SU ( radical* OR extrem* OR terror* OR action OR "politically motivated" OR ideological*)

*Step 6:* TI ( media OR technology OR internet OR online ) OR AB ( media OR technology OR internet OR online ) OR SU ( media OR technology OR internet OR online )

*Step 7:* TI (quantitative OR empirical OR survey OR regression OR multivariate OR correlation OR experiment* OR manipulat* OR coefficient* OR covariat* OR longitudinal OR paramet* OR predict* OR questionnaire* OR sampl* OR "standard deviation*" OR statistic* OR variable* OR variance ) OR AB ( quantitative OR empirical OR survey OR regression OR multivariate OR correlation OR experiment* OR manipulat* OR coefficient* OR covariat* OR longitudinal OR paramet* OR predict* OR questionnaire* OR sampl* OR "standard deviation*" OR statistic* OR variable* OR variance ) OR SU ( quantitative OR empirical OR survey OR regression OR multivariate OR correlation OR experiment* OR manipulat* OR coefficient* OR covariat* OR longitudinal OR paramet* OR predict* OR questionnaire* OR sampl* OR "standard deviation*" OR statistic* OR variable* OR variance )

*Step 8:* S5 AND S6 AND S7 (Limiters - Publication Date: 19900101-20200831)

*Step 9:* S8 NOT S4

**Criminal Justice Abstracts, Political Science Abstracts [2000-]**

**Searches conducted in April 2022**

**Searches conducted on two databases jointly**

**Total results=311**

*Step 1:* TI(Radical* OR Extrem* OR Terror* OR Action OR Politically motivated* OR

Ideological*) AND TI (Media OR Technology OR Internet OR Online) AND TI (quantitative OR empirical OR survey OR regression OR multivariate OR correlation OR experiment* OR manipulat* OR coefficient* OR covariat* OR longitudinal OR paramet* OR predict* OR questionnaire* OR sampl* OR standard deviation* OR statistic* OR variable* OR variance OR AB(Radical* OR Extrem* OR Terror* OR Action OR Politically motivated* OR Ideological*) AND AB (Media OR Technology OR Internet OR Online) AND AB quantitative OR empirical OR survey OR regression OR multivariate OR correlation OR experiment* OR manipulat* OR coefficient* OR covariat* OR longitudinal OR paramet* OR predict* OR questionnaire* OR sampl* OR standard deviation* OR statistic* OR variable* OR

variance)

*Step 2:* TI(Radical* OR Extrem* OR Terror* OR Action OR Politically motivated* OR

Ideological*) OR AB (Radical* OR Extrem* OR Terror* OR Action OR Politically motivated* OR Ideological*) OR SU (Radical* OR Extrem* OR Terror* OR Action OR Politically motivated* OR Ideological*)

*Step 3:* TI(Media OR Technology OR Internet OR Online) OR AB (Media OR Technology OR Internet OR Online) or SU (Media OR Technology OR Internet OR Online)

*Step 4:* TI (quantitative OR empirical OR survey OR regression OR multivariate OR correlation OR experiment* OR manipulat* OR coefficient* OR covariat* OR longitudinal OR paramet* OR predict* OR questionnaire* OR sampl* OR standard deviation* OR statistic* OR variable* OR variance OR AB(Radical* OR Extrem* OR Terror* OR Action OR Politically motivated* OR Ideological*) AND AB (Media OR Technology OR Internet OR Online) AND AB quantitative OR empirical OR survey OR regression OR multivariate OR correlation OR experiment* OR manipulat* OR coefficient* OR covariat* OR longitudinal OR paramet* OR predict* OR questionnaire* OR sampl* OR standard deviation* OR statistic* OR variable* OR variance) OR AB (quantitative OR empirical OR survey OR regression OR multivariate OR correlation OR experiment* OR manipulat* OR coefficient* OR covariat* OR longitudinal OR paramet* OR predict* OR questionnaire* OR sampl* OR standard deviation* OR statistic* OR variable* OR variance OR AB(Radical* OR Extrem* OR Terror* OR Action OR Politically motivated* OR Ideological*) AND AB (Media OR Technology OR Internet OR Online) AND AB quantitative OR empirical OR survey OR regression OR multivariate OR correlation OR experiment* OR manipulat* OR coefficient* OR covariat* OR longitudinal OR paramet* OR predict* OR questionnaire* OR sampl* OR standard deviation* OR statistic* OR variable* OR variance) OR SU (quantitative OR empirical OR survey OR regression OR multivariate OR correlation OR experiment* OR manipulat* OR coefficient* OR covariat* OR longitudinal OR paramet* OR predict* OR questionnaire* OR sampl* OR standard deviation* OR statistic* OR variable* OR variance OR AB(Radical* OR Extrem* OR Terror* OR Action OR Politically motivated* OR Ideological*) AND AB (Media OR Technology OR Internet OR Online) AND AB quantitative OR empirical OR survey OR regression OR multivariate OR correlation OR experiment* OR manipulat* OR coefficient* OR covariat* OR longitudinal OR paramet* OR predict* OR questionnaire* OR sampl* OR standard deviation* OR statistic* OR variable* OR variance)

*Step 5:* S2 AND S3 AND S3

*Step 6:* S5 NOT S1 (Limiters-Publication Date 20200831)

**PsychInfo, APA PsycNet**

**Searches conducted in August 2020**

**Total results: 6,250**

Abstract: radical* OR extrem* OR terror* OR action OR “politically motivated*” OR ideological* *AND* Abstract: media *OR* Abstract: technology *OR* Abstract: internet *OR* Abstract: online *AND* Abstract: quantitative *OR* Abstract: empirical *OR* Abstract: survey *OR* Abstract: regression *OR* Abstract: multivariate *OR* Abstract: correlation *OR* Abstract: experiment* *OR* Abstract: manipulat* *OR* Abstract: coefficient* *OR* Abstract: covariat* *OR* Abstract: longitudinal *OR* Abstract: paramet* *OR* Abstract: predict* *OR* Abstract: questionnaire* *OR* Abstract: sampl* *OR* Abstract: standarddeviation* *OR* Abstract: statistic* *OR* Abstract: variable* *OR* Abstract: variance *OR* Title: Radical* *OR* Title: Extrem* *OR* Title: Terror* *OR* Title: Action *OR* Title: Politicallymotivated* *OR* Title: Ideological* *AND* Title: media *OR* Title: technology *OR* Title: internet *OR* Title: online *AND* Title: quantitative *OR* Title: empirical *OR* Title: survey *OR* Title: regression *OR* Title: multivariate *OR* Title: correlation *OR* Title: experiment* *OR* Title: manipulat* *OR* Title: coefficient* *OR* Title: covariat* *OR* Title: longitudinal *OR* Title: paramet* *OR* Title: predict* *OR* Title: questionnaire* *OR* Title: sampl* *OR* Title: standard deviation* *OR* Title: statistic* *OR* Title: variable* *OR* Title: variance

**PsychInfo, APA PsycNet**

**Search conducted in April 2022**

**Total results: 2,505**

((((title: (quantitative)) OR (title: (empirical)) OR (title: (survey)) OR (title:(regression)) OR (title: (multivariate)) OR (title: (correlation)) OR (title:(experiment*)) OR (title: (manipulat*)) OR (title: (coefficient*)) OR (title:(covariat*)) OR (title: (longitudinal)) OR (title: (paramet*)) OR (title: (predict*)) OR (title: (questionnaire*)) OR (title: (sampl*)) OR (title: (standard deviation*)) OR (title: (statistic*)) OR (title: (variable*)) OR (title: (variance))) OR ((abstract: (quantitative)) OR (abstract: (empirical)) OR (abstract: (survey)) OR (abstract: (regression)) OR (abstract: (multivariate)) OR (abstract: (correlation)) OR (abstract: (experiment*)) OR (abstract: (manipulat*)) OR (abstract: (coefficient*)) OR (abstract: (covariat*)) OR (abstract: (longitudinal)) OR (abstract: (paramet*)) OR (abstract: (predict*)) OR (abstract: (questionnaire*)) OR (abstract: (sampl*)) OR (abstract: (standard deviation*)) OR (abstract: (statistic*)) OR (abstract: (variable*)) OR (abstract: (variance))) OR ((Keywords: (quantitative)) OR (Keywords: (empirical)) OR (Keywords: (survey)) OR (Keywords: (regression)) OR (Keywords: (multivariate)) OR (Keywords: (correlation)) OR (Keywords: (experiment*)) OR (Keywords: (manipulat*)) OR (Keywords: (coefficient*)) OR (Keywords: (covariat*)) OR (Keywords: (longitudinal)) OR (Keywords: (paramet*)) OR (Keywords: (predict*)) OR (Keywords: (questionnaire*)) OR (Keywords: (sampl*)) OR (Keywords: (standard deviation*)) OR (Keywords: (statistic*)) OR (Keywords: (variable*)) OR (Keywords: (variance)))) AND (((title: (media)) OR (title: (technology)) OR (title: (internet)) OR (title: (online))) OR ((abstract: (media)) OR (abstract: (technology)) OR (abstract: (internet)) OR (abstract: (online))) OR ((Keywords: (media)) OR (Keywords: (technology)) OR (Keywords: (internet)) OR (Keywords: (online)))) AND (((title: (radical*)) OR (title: (extrem*)) OR (title: (terror*)) OR (title: (action)) OR (title: ("politically motivated*")) OR (title: (ideological*))) OR ((abstract: (radical*)) OR (abstract: (extrem*)) OR (abstract: (terror*)) OR (abstract: (action)) OR (abstract: ("politically motivated*")) OR (abstract: (ideological*))) OR ((Keywords: (radical*)) OR (Keywords: (extrem*)) OR (Keywords: (terror*)) OR (Keywords: (action)) OR (Keywords: ("politically motivated*")) OR (Keywords: (ideological*))))) NOT (((abstract: (radical*) OR abstract: (extrem*) OR abstract: (terror*) OR abstract: (action) OR abstract: ("politically motivated*") OR abstract: (ideological*)) AND (abstract: (media) OR abstract: (technology) OR abstract: (internet) OR abstract: (online)) AND (abstract: (quantitative) OR abstract: (empirical) OR abstract: (survey) OR abstract: (regression) OR abstract: (multivariate) OR abstract: (correlation) OR abstract: (experiment*) OR abstract: (manipulat*) OR abstract: (coefficient*) OR abstract: (covariat*) OR abstract: (longitudinal) OR abstract: (paramet*) OR abstract: (predict*) OR abstract: (questionnaire*) OR abstract: (sampl*) OR abstract: (standard deviation*) OR abstract: (statistic*) OR abstract: (variable*) OR abstract: (variance)) OR (title: (radical*) OR title: (extrem*) OR title: (terror*) OR title: (action) OR title: ("politically motivated*") OR title: (ideological*)) AND (title: (media) OR title: (technology) OR title: (internet) OR title: (online)) AND (title: (quantitative) OR title: (empirical) OR title: (survey) OR title: (regression) OR title: (multivariate) OR title: (correlation) OR title: (experiment*) OR title: (manipulat*) OR title: (coefficient*) OR title: (covariat*) OR title: (longitudinal) OR title: (paramet*) OR title: (predict*) OR title: (questionnaire*) OR title: (sampl*) OR title: (standard deviation*) OR title: (statistic*) OR title: (variable*) OR title: (variance))) AND ((Year: [1965 TO 2020])))

**ERIC-EBSOHOST**

**Search conducted in August, 2020**

**Total results=2,962**

AB (radical* OR extrem* OR terror* OR action OR politically motivated* OR ideological* ) AND AB (media OR technology OR internet OR online ) AND AB ( Quantitative OR Empirical OR Survey OR Regression OR Multivariate OR Correlation OR Experiment* OR Manipulat* OR coefficient* OR covariat* OR longitudinal OR paramet* OR predict* OR questionnaire* OR sampl* OR standard deviation*, OR statistic* OR variable* OR variance ) OR TI ( Radical* OR Extrem* OR Terror* OR Action OR Politically motivated* OR Ideological* ) AND TI ( Media OR Technology OR Internet OR Online ) AND TI ( Quantitative OR Empirical OR Survey OR Regression OR Multivariate OR Correlation OR Experiment* OR Manipulat* OR coefficient* OR covariat* OR longitudinal OR paramet* OR predict* OR questionnaire* OR sampl* OR standard deviation*, OR statistic* OR variable* OR variance ) OR KW ( Radical* OR Extrem* OR Terror* OR Action OR Politically motivated* OR Ideological* ) AND KW ( Media OR Technology OR Internet OR Online ) AND KW ( Quantitative OR Empirical OR Survey OR Regression OR Multivariate OR Correlation OR Experiment* OR Manipulat* OR coefficient* OR covariat* OR longitudinal OR paramet* OR predict* OR questionnaire* OR sampl* OR standard deviation*, OR statistic* OR variable* OR variance)

**ERIC-EBSOHOST**

**Search conducted in April, 2022**

**Total results=1,584**

*Step 1:* TI ( Radical* OR Extrem* OR Terror* OR Action OR Politically motivated* OR Ideological* ) AND TI ( Media OR Technology OR Internet OR Online ) AND TI (Quantitative OR Empirical OR Survey OR Regression OR Multivariate OR Correlation OR Experiment* OR Manipulat* OR coefficient* OR covariat* OR longitudinal OR paramet* OR predict* OR questionnaire* OR sampl* OR standard deviation*, OR statistic* OR variable* OR variance)

*Step 2:* AB ( Radical* OR Extrem* OR Terror* OR Action OR Politically motivated* OR Ideological* ) AND AB ( Media OR Technology OR Internet OR Online ) AND AB (Quantitative OR Empirical OR Survey OR Regression OR Multivariate OR Correlation OR

Experiment* OR Manipulat* OR coefficient* OR covariat* OR longitudinal OR paramet* OR predict* OR questionnaire* OR sampl* OR standard deviation*, OR statistic* OR variable* OR variance)

*Step 3:* S1 OR S2

*Step 4:* TI ( Radical* OR Extrem* OR Terror* OR Action OR Politically motivated* OR Ideological* ) AND TI ( Media OR Technology OR Internet OR Online ) AND TI (Quantitative OR Empirical OR Survey OR Regression OR Multivariate OR Correlation OR Experiment* OR Manipulat* OR coefficient* OR covariat* OR longitudinal OR paramet* OR predict* OR questionnaire* OR sampl* OR standard deviation*, OR statistic* OR variable* OR variance

) OR AB ( Radical* OR Extrem* OR Terror* OR Action OR Politically motivated* OR Ideological* ) AND AB ( Media OR Technology OR Internet OR Online ) AND (Quantitative OR Empirical OR Survey OR Regression OR Multivariate OR Correlation OR Experiment* OR Manipulat* OR coefficient* OR covariat* OR longitudinal OR paramet* OR predict* OR questionnaire* OR sampl* OR standard deviation*, OR statistic* OR variable* OR variance)

*Step 5:* TI ( radical* OR extrem* OR terror*OR action OR "politically motivated" OR ideological* ) OR AB ( radical* OR extrem* OR terror* OR action OR "politically motivated" OR ideological* ) OR SU ( radical* OR extrem* OR terror* OR action OR "politically motivated" OR ideological*)

*Step 6:* TI ( media OR technology OR internet OR online ) OR AB ( media OR technology OR internet OR online ) OR SU ( media OR technology OR internet OR online )

*Step 7:* TI (quantitative OR empirical OR survey OR regression OR multivariate OR correlation OR experiment* OR manipulat* OR coefficient* OR covariat* OR longitudinal OR paramet* OR predict* OR questionnaire* OR sampl* OR "standard deviation*" OR statistic* OR variable* OR variance ) OR AB ( quantitative OR empirical OR survey OR regression OR multivariate OR correlation OR experiment* OR manipulat* OR coefficient* OR covariat* OR longitudinal OR paramet* OR predict* OR questionnaire* OR sampl* OR "standard deviation*" OR statistic* OR variable* OR variance ) OR SU ( quantitative OR empirical OR survey OR regression OR multivariate OR correlation OR experiment* OR manipulat* OR coefficient* OR covariat* OR longitudinal OR paramet* OR predict* OR questionnaire* OR sampl* OR "standard deviation*" OR statistic* OR variable* OR variance )

*Step 8:* S5 AND S6 AND S7 (Limiters - Publication Date: 19900101-20200831)

*Step 9:* S8 NOT S4

**PUBMED (August 26, 2020, search re-performed on 20/1/22)**

**Total results: 16, 423**

((radical*[Title/Abstract] OR extrem*[Title/Abstract] OR terror*[Title/Abstract] OR action[Title/Abstract] OR "politically motivated*"[Title/Abstract] OR ideological*[Title/Abstract]) AND (media[Title/Abstract] OR technology[Title/Abstract] OR internet[Title/Abstract] OR online[Title/Abstract])) AND (quantitative[Title/Abstract] OR empirical[Title/Abstract] OR survey[Title/Abstract] OR regression[Title/Abstract] OR multivariate[Title/Abstract] OR correlation[Title/Abstract] OR experiment*[Title/Abstract] OR manipulat*[Title/Abstract] OR coefficient*[Title/Abstract] OR covariat*[Title/Abstract] OR longitudinal[Title/Abstract] OR paramet*[Title/Abstract] OR predict*[Title/Abstract] OR questionnaire*[Title/Abstract] OR sampl*[Title/Abstract] OR standard deviation*[Title/Abstract] OR statistic*[Title/Abstract] OR variable*[Title/Abstract] OR variance[Title/Abstract])

Table 16: Coding sheet

|  | **Category** | **Data type** | **Selection/Data entry** | **Comments/ Notes** |  |  |  |
| --- | --- | --- | --- | --- | --- | --- | --- |
| **Publication information** |  |  |  |  |  |  |  |
|  | 1. Author(s) name(s) | Text |  |  |  |  |  |
|  | 2. Title | Text |  |  |  |  |  |
|  | 3. Year | Number |  |  |  |  |  |
|  | 4. Full reference | Text |  |  |  |  |  |
|  |  |  |  |  |  |  |  |
|  | Type: | Categorical |  |  |  |  |  |
|  | a. Journal article (peer reviewed) |  |  |  |  |  |  |
|  | b. Book |  |  |  |  |  |  |
|  | c. Government report |  |  |  |  |  |  |
|  | e. Police report |  |  |  |  |  |  |
|  | f. Technical report |  |  |  |  |  |  |
|  | g. Conference paper |  |  |  |  |  |  |
|  | h. Dissertation or thesis |  |  |  |  |  |  |
|  | J. Other (specify) |  |  |  |  |  |  |
|  |  |  |  |  |  |  |  |
|  | **Publication details (complete for each study reported)** |  |  |  |  |  |  |
|  | 5. Location/country of sample | Text |  |  |  |  |  |
|  | 6. Language | Text |  |  |  |  |  |
|  | 7. Date(s) of research | Number |  |  |  |  |  |
|  | a. Start: | Date |  |  |  |  |  |
|  | b. Finish: | Date |  |  |  |  |  |
|  | 8. Source of funding | Categorical |  |  |  |  |  |
|  | a. Government |  |  |  |  |  |  |
|  | b. Foreign government |  |  |  |  |  |  |
|  | c. Local university/research body |  |  |  |  |  |  |
|  | d. Foreign university/research body |  |  |  |  |  |  |
|  | e. NGO |  |  |  |  |  |  |
|  | f. Other |  |  |  |  |  |  |
|  | 9. Bodies involved (tick all applicable) |  |  |  |  |  |  |
|  | a. University/research agency |  |  |  |  |  |  |
|  | b. Health Service |  |  |  |  |  |  |
|  | c. Police/ Justice system |  |  |  |  |  |  |
|  | d. Other government departments |  |  |  |  |  |  |
|  | e. Other |  |  |  |  |  |  |
|  |  |  |  |  |  |  |  |
|  | 10. Evaluated by | Text |  |  |  |  |  |
|  | 11. Conflict context (Y/N)? | Y/N |  |  |  |  |  |
|  | 12. Ethical issues (Y/N. If yes, describe)? | Y/N |  |  |  |  |  |
|  |  |  |  |  |  |  |  |
| **Methodology** |  |  |  |  |  |  |  |
|  | 13. Type of study: | Categorical |  |  |  |  |  |
|  | a. Longitudinal |  |  |  |  |  |  |
|  | b. Cross-sectional |  |  |  |  |  |  |
|  | c. Case control |  |  |  |  |  |  |
|  |  |  |  |  |  |  |  |
|  | 14. Sampling method | Categorical |  |  |  |  |  |
|  | a. Random |  |  |  |  |  |  |
|  | b. Representative |  |  |  |  |  |  |
|  | c. Convenience |  |  |  |  |  |  |
|  | d. Snowball |  |  |  |  |  |  |
|  | e. Other |  |  |  |  |  |  |
|  |  |  |  |  |  |  |  |
|  | 15. Radicalization outcome: | Categorical |  |  |  |  |  |
|  | a. Radical attitudes  b. Radical intentions  c. Radical behaviors |  |  |  |  |  |  |
|  |  |  |  |  |  |  |  |
|  | 15a. Measure of radical attitudes: | Categorical |  |  |  |  |  |
|  | a. Support/justification of radical violence  b. Support/justification of terrorism  c. Support/justification of specific radical event(s)  d. Other  15b. Measure of radical intentions:  a. Willingness/intentions towards carrying out of radical violence  b. Willingness/intentions towards carrying out of terrorism  15c. Measure of radical behaviors:  a. Convicted of radical offences  b. Convicted of terrorism offences  c. Self-reported involvement in radical behaviors |  |  |  |  |  |  |
|  |  |  |  |  |  |  |  |
|  | 16. Measurement level/type |  |  |  |  |  |  |
|  | a. Dichotomous |  |  |  |  |  |  |
|  | b. Ordinal | # Of levels |  |  |  |  |  |
|  | c. Continuous/discrete | /Max |  |  |  |  |  |
|  |  |  |  |  |  |  |  |
|  | 17. Source of measure: | Categorical |  |  |  |  |  |
|  | a. Open source |  |  |  |  |  |  |
|  | b. Official data (e.g., police) |  |  |  |  |  |  |
|  | c. Self-reported |  |  |  |  |  |  |
|  | d. Peer/Family-reported |  |  |  |  |  |  |
|  | e. Practitioner-reported |  |  |  |  |  |  |
|  | f. Other |  |  |  |  |  |  |
|  |  |  |  |  |  |  |  |
|  | 18. Radicalizing ideology examined: | Categorical |  |  |  |  |  |
|  | a. Non-specific/mixed sample |  |  |  |  |  |  |
|  | b. Islamist or sample of Muslims only |  |  |  |  |  |  |
|  | c. Right-wing |  |  |  |  |  |  |
|  | d. Left-wing |  |  |  |  |  |  |
|  | e. Other | Text |  |  |  |  |  |
|  |  |  |  |  |  |  |  |
|  | 19. Author definition of radicalization: | Text |  |  |  |  |  |
|  |  |  |  |  |  |  |  |
|  | 20.Sample size |  |  |  |  |  |  |
|  | a. Total sample size | Numerical |  |  |  |  |  |
|  | b. Sample size of comparison group | Numerical |  |  |  |  |  |
|  |  |  |  |  |  |  |  |
|  | 21. Was attrition a problem? | Y/N |  |  |  |  |  |
|  |  |  |  |  |  |  |  |
|  | 22. Initial response rate | Numerical |  |  |  |  |  |
|  |  |  |  |  |  |  |  |
|  | 23. Sample age  a. Range  b. Mean | Numerical |  |  |  |  |  |
|  |  |  |  |  |  |  |  |
|  | 24. Sample gender (% males) | Numerical |  |  |  |  |  |
|  |  |  |  |  |  |  |  |
|  |  |  |  |  |  |  |  |
| **Risk of bias** |  |  |  |  |  |  |  |
|  | 26. Study sample description. Does the document describe the source | Y/N |  |  |  |  |  |
|  | sample in replicable detail? |  |  |  |  |  |  |
|  |  |  |  |  |  |  |  |
|  | 27. Study population criteria: Does the document list all inclusion and exclusion | Y/N |  |  |  |  |  |
|  | criteria for participation? |  |  |  |  |  |  |
|  |  |  |  |  |  |  |  |
|  | 28. Prospective study: Was the study prospective (i.e., the sample was selected | Y/N |  |  |  |  |  |
|  | prior to the onset of radicalization or involvement in radical activity)? |  |  |  |  |  |  |
|  |  |  |  |  |  |  |  |
|  | 29. Outcome descriptor: Was the criteria for fitting 'radical'/'radicalization'/ | Y/N |  |  |  |  |  |
|  | /'recruited described in replicable detail? |  |  |  |  |  |  |
|  |  |  |  |  |  |  |  |
|  | 30. Outcome validity: Was the outcome measured using a validated instrument? | Y/N |  |  |  |  |  |
|  | 31. Indicator description: Were all factors described in replicable detail? | Y/N |  |  |  |  |  |
|  |  |  |  |  |  |  |  |
|  | 32. Indicator validity: Were all measures of the factor based on a validated | Y/N |  |  |  |  |  |
|  | measure? |  |  |  |  |  |  |
|  |  |  |  |  |  |  |  |
|  | 33. Risk factor timing: Were all factors either measured before the onset of | Y/N |  |  |  |  |  |
|  | radicalization or involvement in radical activity, or measured retrospectively |  |  |  |  |  |  |
|  | to a time prior to radicalization or involvement in radical activity? |  |  |  |  |  |  |
|  |  |  |  |  |  |  |  |
|  | 34. Selective risk factor reporting: was the study free from reporting bias? | Y/N |  |  |  |  |  |
|  |  |  |  |  |  |  |  |
|  | 35. Selective analysis reporting: was the study free from analysis reporting bias? | Y/N |  |  |  |  |  |
|  |  |  |  |  |  |  |  |
|  | 36. Was there missing data? | Y/N |  |  |  |  |  |
|  |  |  |  |  |  |  |  |
| **Indicator/factor (Complete** | | | | |  |  |  |
| **for each factor reported)** | 37. Name of factor | Text |  |  |  |  |  |
|  | 38. Conceptual definition of factor | Text |  |  |  |  |  |
|  | 39.Operational definition | Text |  |  |  |  |  |
|  | 40. Origin of the factor's variable: | Categorical |  |  |  |  |  |
|  | a. Official data (government/police) |  |  |  |  |  |  |
|  | b. Self-reported |  |  |  |  |  |  |
|  | c. Peer-reported |  |  |  |  |  |  |
|  | d. Family-reported |  |  |  |  |  |  |
|  | e. Practitioner-reported (including school) |  |  |  |  |  |  |
|  | f. Other |  |  |  |  |  |  |
|  |  |  |  |  |  |  |  |
|  | 41. Measured retrospectively? | Y/N |  |  |  |  |  |
|  |  |  |  |  |  |  |  |
|  | 42. Time-invariant risk factor? (If the study design is not longitudinal and the | Y/N |  |  |  |  |  |
|  | factor is not time-invariant, the factor will be classified as a putative factor) |  |  |  |  |  |  |
|  |  |  |  |  |  |  |  |
|  |  |  |  |  |  |  |  |
|  | 43. Factor domain: | Categorical |  |  |  |  |  |
|  | a. Passive |  |  |  |  |  |  |
|  | b. Active |  |  |  |  |  |  |
|  | 43b. Secondary domain |  |  |  |  |  |  |
|  | a. Internet media |  |  |  |  |  |  |
|  | b. Other media |  |  |  |  |  |  |
|  |  |  |  |  |  |  |  |
|  | 44. Did a test of statistical significance indicate statistically significant | Categorical |  |  |  |  |  |
|  | differences between those displaying the outcome and those without the outcome/differing levels of the outcome? |  |  |  |  |  |  |
|  |  |  |  |  |  |  |  |
|  | 45. Was a standardized effect size reported? | Y/N |  |  |  |  |  |
|  | 46. Effect size measure  a. *r*  b. *b*  c. B(exp)  d. OR  e. Other | Categorical |  |  |  |  |  |
|  | 47. Effect size in Fisher's Z | Numerical |  |  |  |  |  |
|  | 48. Standard error of effect size | Numerical |  |  |  |  |  |
|  | 49. Effect size reported on page number | Numerical |  |  |  |  |  |
|  |  |  |  |  |  |  |  |
|  | 50. If no effect size reported, are data available to calculate effect size? | Y/N |  |  |  |  |  |
|  |  |  |  |  |  |  |  |
|  | 51. If yes to Q50, type of data effect size can be calculated from: | Categorical |  |  |  |  |  |
|  | a. Means and standard deviations |  |  |  |  |  |  |
|  | b. Frequencies or proportions (dichotomous) |  |  |  |  |  |  |
|  | c. Frequencies or proportions (polychotomous) |  |  |  |  |  |  |
|  | d. Unadjusted correlation coefficient |  |  |  |  |  |  |
|  | e. Multiple regression coefficients (unstandardized) |  |  |  |  |  |  |
|  | f. Multiple regression coefficients (standardized) |  |  |  |  |  |  |
|  | g. Odds ratio(s) |  |  |  |  |  |  |
|  | h. t-value or F-value |  |  |  |  |  |  |
|  | i. Chi-square (df=1) |  |  |  |  |  |  |
|  | j. Other (specify) |  |  |  |  |  |  |
|  |  |  |  |  |  |  |  |
|  | Means and Standard Deviations |  |  |  |  |  |  |
|  | 52. Radicalized/Radicalizing group mean | Numerical |  |  |  |  |  |
|  | 53.Comparison group mean | Numerical |  |  |  |  |  |
|  | 54.Radicalized/Radicalizing group standard deviation | Numerical |  |  |  |  |  |
|  | 55. Comparison group standard deviation | Numerical |  |  |  |  |  |
|  |  |  |  |  |  |  |  |
|  | Proportions or frequencies |  |  |  |  |  |  |
|  | 56. n of radicalized/radicalizing group with the risk factor(s) | Numerical |  |  |  |  |  |
|  | 57.n of comparison group with the risk factor(s) | Numerical |  |  |  |  |  |
|  | 58. Proportion of radicalized/radicalizing group with the risk factor | Numerical |  |  |  |  |  |
|  | 59.Proportion of comparison group with the risk factor | Numerical |  |  |  |  |  |
|  |  |  |  |  |  |  |  |
|  | Regression coefficients and correlations |  |  |  |  |  |  |
|  | 60.Unadjusted correlation coefficient | Numerical |  |  |  |  |  |
|  | 61.Standardized regression coefficient | Numerical |  |  |  |  |  |
|  | 62.Unstandardized regression coefficient | Numerical |  |  |  |  |  |
|  | 63.Standard deviation of predictor | Numerical |  |  |  |  |  |
|  | 64.Control variables | Numerical |  |  |  |  |  |
|  |  |  |  |  |  |  |  |
|  | Significance Tests |  |  |  |  |  |  |
|  | 65.t-value | Numerical |  |  |  |  |  |
|  | 66.F-value | Numerical |  |  |  |  |  |
|  | 67.Chi-square value (df=1) | Numerical |  |  |  |  |  |
|  |  |  |  |  |  |  |  |
|  | Calculated Effect Size |  |  |  |  |  |  |
|  | 68.Effect size | Numerical |  |  |  |  |  |
|  | 69.Standard error of effect size | Numerical |  |  |  |  |  |
|  |  |  |  |  |  |  |  |
| **Authors’ conclusion** | | | | |  |  |  |
|  | 70. What did the authors conclude about the relationship? | Categorical/Text |  |  |  |  |  |
|  | a. Risk factors increases likelihood of radicalization and/or recruitment |  |  |  |  |  |  |
|  | b. Risk factor reduces the likelihood of radicalization and/or recruitment |  |  |  |  |  |  |
|  | c. No effect of risk factor on radicalization and/or recruitment |  |  |  |  |  |  |
|  | d. Unclear/no conclusion stated by authors |  |  |  |  |  |  |

Table 17: Measurement of outcomes and indicators

| ***Study*** | **Outcome measure** | | **Factor measure(s)** | |  |
| --- | --- | --- | --- | --- | --- |
| *Azeem et al (2002)* | | Killing in the name of Islam=1 item, 1-10 scale | | Experimentally manipulated exposure to news headlines | |
| *Baier (2010)* | Right-wing extremism=9, 1-7 scale | | Exposure to violent films, television, and video games=1-7 scale | |  |
| *Berger (2016)* | Justification of suicide bombings=1 item, 1-4 scale, dichotomized | | Main source of religious guidance is television=Dichotomized | |  |
| *Bhatia & Ghanem (2017)* | Justification of 9/11, 1-5 scale | | Television usage=Dichotomous; Internet usage=Dichotomous | |  |
| *Bhui et al (2016)* | Justification of terrorism (SyFor)=16 items,1-7 scale, α=.81 | | Expressed political opinions online, binary measure | |  |
| *Brunsting & Postmes (2002)* | | Sending mail bombs=1 item, 1-7 scale | | Online activism, hacking, intentions to online activism, intentions to hacking, efficacy of online activism, efficacy of hacking=items measured on 7-point scales. | |
| *Calenda & Mosca (2007)* | Radical or non-radical behaviors, measured dichotomously | | Political use of the internet, 4 items measured dichotomously | |  |
| *Charkawi et al (2020)* | 6 items on a 5-point scale, including justification of jihad and suicide bombings | | Media bias-6 items on 5-point scale, Media usage, 6 television, radio, and print media outlets on a 5-point scale | |  |
| *Clemmow et al (2020)* | Lone actor terrorists from an open-source sample (1) compared to general population (0), measured dichotomously | | Searched online for extremist materials (Dichotomous), Virtual interactions with extremists online (Dichotomous), | |  |
| *Ellis et al (2016)* | Activism-Radicalism-Intentions Scale (ARIS)= 4 items, 1-7 scale, α=.74 | | Internet usage=1-13 hours/day, single item; Importance of online community, single item, 1-7 scale | |  |
| *Ellis et al (2019)* | Activism-Radicalism-Intentions Scale (ARIS)=5 items, 1-7 scale, α=.85 | | Online network attachment=7-item Social Comfort subscale of the Online Cognition Scale (OCS), 1-7 scale (α=.67) | |  |
| *Eyal et al (2006)* | Aggressive Political Opinion (APO)=16 items, 1-5 scale | | Violent television exposure=1-4 scale of exposure to 23 different shows; Violent video games=1-4 scale of playing of 42 different games | |  |
| *Fair & Patel (2019)* | Justification of suicide bombings, 1-3 scale | | Internet access=Single item dichotomous | |  |
| *Fair & Salva (2019)* | Justification of suicide bombings, 1-3 scale | | Internet access=Single item dichotomous | |  |
| *Frissen (2019)* | Justification of terrorism=9 items from SyFor (Bhui et al., 2014), 1-5 scale; ARIS, 4 items, 1-5 scale, α=.79 | | Searching for radical material=6 types, dichotomous, α=.88; Sources of online information=6 types, dichotomous | |  |
| *Frissen (2020)* | 4 items from SyFor (Bhui et al., 2014), 1-5 scale, α=.85 | | Searching for radical material=6 types, dichotomous (α=.88); Sources of online information=6 types, dichotomous | |  |
| *Frissen et al (2019)* | SyFor (Bhui et al., 2014), 16 items, 1-5 scale (α=.88-.90); ARIS, 4 items, 1-5 scale, α=0.89) | | Consumption of political news=1-5 scale; Passive exposure to radical content= 8 types, 1-5 scale; Active searching of radical content=8 types of ISIS and Al-Qaeda content, 1-5 scale | |  |
| *Gentzkow & Shapiro (2004)* | Justification of 9/11, 1-4 scale dichotomized | | Frequency of media usage=# of days for: read daily newspapers/watch TV/listen to the radio, dichotomized; Source of news=Al Jazeera or CNN, dichotomized; Sites where radical content was encountered=Dichotomized | |  |
| *Goede et al (2019)* | Right-wing extremism=16 items, 1-5 scale (α=.87); Islamist extremism=15 items, 1-5 scale (α=.89); Left-wing extremism=11 items, 1-5 scale (α=.80) | | Victim of cyberbullying=1 item, 1-5 scale; Frequency of internet use=1 item, ; Finding friends on the internet=1 item; Life without internet is unimaginable=1 item; Careful with personal data; Enjoy giving 'likes'; Seen violent content online=; Heavy use of online messengers; Use of online social networking; Parents know about internet activities; Television or Radio usage=; Newspaper or magazine usage; Facebook and Twitter usage; Usage of other apps and websites; Posting of violent extremist content online; Consuming violent extremist content online=; Visiting political websites | |  |
| *Gvirsman et al (2016)* | Normative beliefs about aggression toward out-group=7 items from Huesmann and Guerra’s (1997) Normative Beliefs About Aggression, 1-4 scale (α=.95-.96) | | Mediated Exposure to ethnic-political conflict and violence=6 items measuring exposure through media (e.g., videos) derived from Slone, Lobel, and Gilat (1999), 4-point scale (α=.72-.83) | |  |
| *Hawthorne (2016)* | Acceptance of Political Violence=6 items, 1-7 scale (α=.83-.88) | | Pro-attitudinal media use=; Frequency of political social media use on Facebook and Twitter=; Positive social media communication; Negative social media communication | |  |
| *Holt & Kilger (2012)* | Willingness to engage in radical acts=8 scenarios (including bombings) measured dichotomously | | Cyber technical skills=3 items, 1-6 scale (α = .856); Online piracy=2 items, 0-4 scale (α = .656); Willingness to carry out politically motivated cyber-attack=9 items | |  |
| *Holt et al (2017)* | Willingness to engage in radical acts=8 scenarios (including bombings) measured dichotomously | | Cyber technical skills=8 items, 1-5 scale (α = .866); Time online=1-5 scale; Online piracy=2 items, 1-4 scale (α = .759); Willingness to carry out politically motivated cyber-attack=5 items, 1-4 scale | |  |
| *Jones & Paris (2018)* | | Legitimacy of radical political action=5 items and 3 items, 100-point slider scale (α=.83, .82) | | Experimentally manipulated exposure to clips from action films and broadcasts of political protests | |
| *Kalmoe (2014)* | Justification of political violence=5 items, 1-5 scale (α=.69-.84) | | Experimental exposure to violent political advertisement | |  |
| *Kaltenthaler et al (2018)* | Support for ISIS=1 item, 1-4 scale | | ISIS news=Single item dichotomized | |  |
| *Kremerman et al (2012)* | 12 items from the aggressive political opinions (APO) scale (including justification of radical violence), measured on a 1-4 scale (α=.75) | | Violent television exposure=1-4 scale of exposure to 24 different shows | |  |
| *LaFree & Morris (2012)* | Support for Al-Qaeda=Dichotomized from a 1-4 scale | | Exposure to international issues through international issues through print, television media=1-7 scale (α=.70) | |  |
| *Lee (2018)* | Attitudes towards radical tactics=6 items (including use of force), 1-5 scale (*r*=.73) | | Political media=4 items, 1-4 scale (α=.77); Self-censorship online=1-4 scale on 8 social/public affairs issues; News acquisition on Facebook=1-4 scale | |  |
| *Luchsinger (2018, 2020)* | Support for ISIS=3 items, 1-7 scale (α=.74) | | Exposure to ISIS media=Exposure on 8 types of media platforms, 1-5 scale (α =.86) | |  |
| *Manzoni et al (2019)* | Right-wing extremism=18 items, 1-6 scale (α=.93); Left-wing extremism, 7 items, 1-6 scale (r=.30); Islamist extremism=21 items, 1-6 scale (α=.80) | | Extremist content consumption=3 items, 1-5 scale; Violent media consumption=3 items, 1-7 scale; Cyberbullying victim=1 item, 1-6 scale | |  |
| *Mock (2014)* | Justification of suicide bombings=1-4 scale | | Internet access=Single item dichotomous | |  |
| *Mourão et al (2016)* | Support for radical protests (including violent overthrow of government) =3 items, 1-10 scale (α=.69) | | Online networking=Use of 3 social media platforms for consuming and sharing political content | |  |
| *Mulyana (2020)* | N/R | | Frequency of social media usage; Duration of time user has been on social media, N/R | |  |
| *Nivette et al (2017)* | 4 items, 1-4 scale (α=.80) | | Violent media exposure=5 items pertaining to different mediums, 1-7 scale (α=.80) | |  |
| *Pauwels & Schils (2016) Pauwels & Hardyns (2018) Pauwels et al (2020)* | Support for violent extremism=9 items, 3 scales combined, 1-5 scale (α=.92); Violent extremism against persons=5 items, 4-point scale (α=.87) | | Active communication with extremists online=3 items, 6-point scale (α=.69); Exposure to extremist content online=5 items, 6-point scale (α=.78); Exposure to extremist content through traditional media=3 items, 6-point scale (α=.82) | |  |
| *Pedersen et al (2018)* | 3 items, 1-4 scale, dichotomized (α=.91) | | Social media to express political opinions=1 item, 1-4 scale | |  |
| *Piazza & Guler (2019)* | Support for ISIS=3 items, 1-3 scale (α=.75) | | General internet use=1 item, 1-6 scale; Political internet use (consumption)=1 item, 1-6 scale, dichotomized; Political opinion expression online= 1 item, 1-6 scale, dichotomized | |  |
| *Sahani (2018)* | Violent extremists (1) and non-violent extremists (0) from PIRUS database | | Radical social media use=dichotomous | |  |
| *Schbley (2004)* | Islamist extremism=7 items, 1-4 scale | | Media exposure=20 items, 1-4 scale, measuring influence of media on beliefs about terrorism | |  |
| *Schröder et al (2020)* | Right-wing extremism=12 items, 1-5 scale (α=.79); Left-wing extremism=11 items, 1-5 scale (α=.66); Islamist extremism=10 items, 1-5 scale (α=.80) | | Visiting websites with political content=1 item, 1-4 scale; Posting political content=1 item, 1-4 scale; Sharing political content online=1 item, 1-4 scale | |  |
| *Schumann et al (2020)* | Support for terrorism= 4 items, 1-5 scale (α=.91); Support for ISIS=2 items, 1-5 scale (α=.88) | | Medias used to receive information about news and current affair=6 items, dichotomized | |  |
| *Shortland et al (2020)* | Militant Extremist Mindset (MEM; Stankov et al., 2010) =24 items, dichotomized (α=.81). | | Experimental exposure to radical group propaganda video | |  |
| *Sirgy et al (2019)* | Justification of suicide bombings, 1-3 scale | | Internet usage=Dichotomous | |  |
| *Storm et al (2020)* | Justification of political violence=3 items, 1-5 scale | | Experienced harassment over the internet=1-6 scale, dichotomized | |  |
| *Tang et al (2020)* | Support for radical actions=8 items, 1-5 scale, α=.96 | | Political movement information acquisition on social media=6 sources, 1-5 scale (α=.80); Internet addiction=5 items from Young’s Internet Addiction Test, measured on a 1-5 scale (α=.86) | |  |
| *Wojcieszak (2010)* | Justification of extremist violence=10 items, 1-7 scale | | Participation in ideological groups online=2 items, open ended (α=.76) | |  |
| *Wolfowicz et al (FC)* | Justification of suicide bombings, 1-3 scale | | Attachment to online network; 1 item, 1-3 scale; Post violent content= 1 item, dichotomous; Viewed radical content=1 item, dichotomous; Facebook usage=1 item, 1-5 scale; Twitter usage=1 item, 1-5 scale; Use internet for political information=1 item, 1-5 scale | |  |
| *Wong et al (2019)* | Activism-Radicalism-Intentions Scale (ARIS)=8 items, 1-7 scale (α=.81-.85) | | Used newspapers, television (TV) news, radio news, social media= measured dichotomously | |  |
| *Zhu et al (2020)* | Engagement in radical behaviours=10 behaviours measured dichotomously | | Social media use=frequency of 8 activities, 1-5 scale; Online political communication=4 items, 1-5 scale; Social media network heterogeneity=7 items, 1-5 scale | |  |
